# A Prognostic Neuromodulation-Related Gene Signature Identifies Immunomodulation and Tumour-Associated Hallmarks in Glioblastoma

**DOI:** 10.3390/biomedicines13112640

**Published:** 2025-10-28

**Authors:** Min Yee Chow, Sylvia Sue Xian Liew, Mastura Monif, Muhamad Noor Alfarizal Kamarudin, Brandon Wee Siang Phon

**Affiliations:** 1 Jeffrey Cheah School of Medicine and Health Sciences, Monash University Malaysia, Bandar Sunway 47500, Malaysia; minyee.chow@monash.edu (M.Y.C.); sylvia.liew@monash.edu (S.S.X.L.); 2 Department of Neuroscience, School of Translational Medicine, Monash University, Melbourne, VIC 3004, Australia; mastura.monif@monash.edu; 3 Department of MS and Neuroimmunology, Alfred Health, Melbourne, VIC 3004, Australia

**Keywords:** glioblastoma, neuromodulators, immunomodulation, prognostic gene signature, risk score, tumour-associated hallmarks, tumour progression

## Abstract

**Background and Objective**: Neuromodulators such as neuropeptide, neurotrophic factors and neurotransmitters are increasingly reported to be involved in glioblastoma (GBM) progression. Nonetheless, the association between neuromodulation-related genes (NMRGs) and GBM prognosis remains elusive. Hence, this study aims to identify clinically significant NMRGs that can form a prognostic gene signature for GBM patients. **Methods and Results**: Differential expression analysis of transcriptomic profiles extracted from GSE147352, GSE165595, TCGA and CGGA determined 272 differentially expressed NMRGs (deNMRGs) in GBM compared to normal brain tissue. The subsequent Kaplan–Meier survival analysis and Cox proportional hazard model further identified ten common deNMRGs (*IGF2*, *RETN*, *EDNRB*, *C3AR1*, *CLCF1*, *NTRK1*, *OSMR*, *KCNN4*, *SLC18A3* and *HTR7*), forming a 10-NMRG signature. This signature stratifies GBM patients and consistently predicts poorer survival outcomes for the high-risk score group compared to the low-risk score group in the TCGA and CGGA cohorts. The gene set enrichment analysis and active-subnetwork-oriented enrichment analysis identified a connection between immunomodulation and tumour-associated hallmarks with the high-risk GBM patient group. Next, the correlation proportionality analysis identified a positive association between the signature genes with immune activators, immune suppressors and pro-motility genes. Additionally, high expressions of the 10-NMRGs were noted in the mesenchymal GBM subtype. **Conclusions**: Collectively, our analysis highlights the potential use of the 10-NMRG signature to stratify the high-risk GBM group with a strong association of immunomodulation and tumour-associated hallmarks that can contribute to the poor survival outcomes.

## 1. Introduction

The glioblastoma *IDH*-wildtype (GBM) is the most common and aggressive form of brain malignancy, accounting for about 50.9% of all malignant brain tumours [1]. Despite advances in molecular diagnostics and therapeutic efforts for GBM, the median survival of GBM patients has barely improved over the last decade, with only a relative estimate of 6.8% of patients surviving five years post-diagnosis [2]. This dismal prognosis underscores the critical need for continued research to develop novel prognostic tools for improved treatment stratification and patient outcomes.

A growing body of evidence has demonstrated that regulators of neuronal functions and synaptic transmission (neuropeptides, neurotrophic factors, neurotransmitters and their associated receptors) play vital roles in GBM tumorigenesis and progression. Notably, the activation of tachykinin NK1 receptors by the neuropeptide substance P induces cytokine production and promotes tumour growth [3]. Similarly, the specific activation of neurotrophic factors, receptors TrkB and TrkC by their ligands neurotrophin 3 (NT3) and brain-derived neurotrophic factor (BDNF) via Akt and ERK pathways, promotes the survival of brain tumour-initiating cells [4]. Furthermore, signalling by neurotransmitters such as acetylcholine [5,6] and glutamate [7,8] has been shown to regulate GBM tumour growth, proliferation, survival, migration, and invasion. Collectively, these findings highlight the critical roles of neuromodulator signalling in GBM pathogenesis, prompting the urgent need to elucidate its clinical potential to enhance diagnostic accuracy and treatment outcomes in GBM patients.

While previous studies have shown that neuromodulators contribute to GBM aggressiveness, their potentials as prognostic markers and therapeutic targets are still not well understood. Recently, the construction of a predictive prognostic assessment model with combined multiple related genes has provided a significant improvement in prediction accuracy, compared to a single gene prognostic marker. In this study, we sought to identify clinically significant neuromodulation-related genes (NMRGs) that could serve as a prognostic signature for GBM patients. The differentially expressed NMRGs (deNMRGs) were systematically screened from The Cancer Genome Atlas (TCGA), Chinese Glioma Genome Atlas (CGGA), and Gene Expression Omnibus (GEO) databases. The common prognostic deNMRGs were integrated to form a predictive model consisting of a 10-neuromodulation-related gene (10-NMRG) signature. Findings from this study showed strong associations of these genes with immunomodulation and tumour-associated hallmarks, contributing to poor survival outcomes in GBM patients.

## 2. Materials and Methods

**Figure 1:**
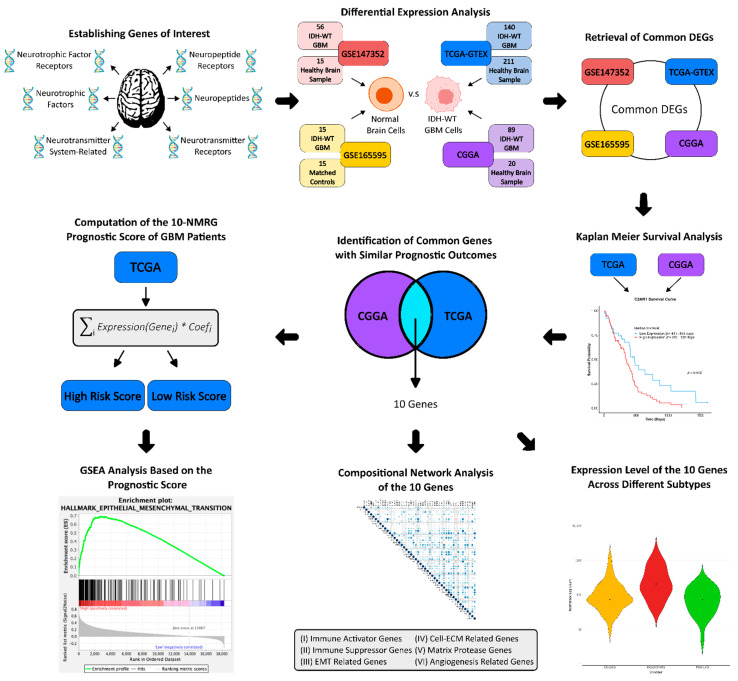
Workflow of the study. Differential expression analysis was performed across four RNA-Seq datasets (TCGA, CGGA, GSE147532, and GSE165595) to identify neuromodulation-related genes (NMRGs) dysregulated in GBM compared to normal brain tissues. NMRGs with concordant expression trends in at least two datasets were evaluated for prognostic value in TCGA and CGGA cohorts. Genes consistently associated with similar survival outcomes were used to construct the 10-NMRG signature. The prognostic performance of this signature was evaluated and validated in both cohorts. GSEA was conducted to characterise high-risk score GBM stratified by the gene signature. Compositional proportionality analysis was performed to understand the relationship between signature genes with immune-related genes and pro-motility genes, while expression patterns of the signature genes were examined across different GBM subtypes. GBM—glioblastoma; DEGs—differentially expressed genes; TCGA—The Cancer Genome Atlas; CGGA—Chinese Glioma Genome Atlas; GTEX—genotype-tissue expression; GSEA—gene set enrichment analysis.

The study workflow is summarised in Figure 1. The fifth edition of the WHO Classification of the Tumour of the Central Nervous System (CNS) released in 2021 (WHO CNS5) has refined GBM as a Grade 4 astrocytic glioma with wildtype *IDH* [9], while diffuse gliomas with *IDH* mutation are defined as lower grade glioma. Hence, for accuracy, only GBM patients with *IDH*-wildtype were included in this study. Their clinicopathological data are summarised in Table S1, Supplementary File S1.

### 2.1. Human Neuromodulation-Related Gene Set

A human neuromodulation-related gene set (n = 420) comprising (1) neuropeptides (n = 97), (2) neuropeptide receptors (n = 97) (Table S2, Supplementary File S1), (3) neurotrophic factors (n = 26), (4) neurotrophic factor receptors (n = 22) (Table S3, Supplementary File S1), (5) neurotransmitter receptors (n = 131) (Table S4, Supplementary File S1) and (6) neurotransmitter system-related genes (n = 47) (Table S5, Supplementary File S1) was established.

### 2.2. RNA-Seq Data Acquisition

Four RNA-Seq expression datasets containing *IDH1R132*-wildtype GBM patients and normal brain samples were obtained. Two RNA-Seq expression datasets with their respective datasets’ clinical information were downloaded from GEO (https://www.ncbi.nlm.nih.gov/geo/) (accessed on 2 July 2023). Huang et al. graciously provided the raw expression count for the dataset GSE147352, which was directly obtained from GEO [10]. For GSE165595, that encompasses matched GBM and brain samples [11], RNA-Seq fastq files were obtained using SRA Explorer instead, where paired-end alignments and transcript quantifications were performed in-house. STAR [12] was leveraged to align the transcripts with Ensembl’s reference human genome (GRCh38 primary assembly), while gene quantifications were performed with the help of RSEM [13], using ENCODE3’s STAR-RSEM pipeline parameters. Exact linux scripts and parameters used are provided.

Additionally, level 3 gene expression profiles of GBM patients from TCGA and healthy brain samples from TCGA (5 samples) and GTEx (206 samples from the cortex and frontal cortex region), specifically Illumina Hiseq2000 RNASeq log2 transformed HTSeq counts, were obtained from the UCSC Xena data portal (https://xenabrowser.net/datapages/) [14] (accessed on 2 July 2023). Clinical data of the GBM patients containing information about the *IDH1R132* mutation status, molecular subtypes, survival times and overall survival outcomes were obtained from cBioPortal (https://www.cbioportal.org) [15,16,17] (accessed on 2 July 2023). The utilisation of the Toil pipeline allowed for a unified processing workflow between the TCGA and GTEx datasets, with STAR being used to generate alignments and quantifications being performed using RSEM [18]. The recomputation of the raw RNA-Seq data from TCGA and GTEx by the UCSC Xena project makes the two datasets compatible, allowing for the direct expression analyses. Following the acquisition of expression data from the TCGA/GTEx cohort, RNA-Seq expression counts were also obtained from CGGA (http://www.cgga.org.cn/download.jsp) [19,20,21,22] (accessed on 3 July 2023). The Illumina HiSeq expression counts and the corresponding clinical information for batch mRNAseq_325 were obtained. Table 1 summarises the number of GBM patients and normal brain samples in each RNA-Seq cohort.

### 2.3. Differential Expression Analysis

Differential expression analysis was performed in R v4.3.2, using the package ‘DESeq2’ (v1.48.2) [23]. |Log2 fold change (LFC)| > 0.6 and Benjamini–Hochberg adjusted *p*-values (*p*adj) < 0.05 were set as the cut-offs for the screening of differentially expressed genes (DEGs). To reduce the problem of exaggerated LFCs for genes with low counts, LFC shrinkage was performed using the estimator ‘apeglm’ [24]. Additionally, gene expression between different GBM subtypes was also analysed and plotted using the ‘plotCounts’ function from DESeq2. All R scripts used for differential expression analysis and subsequent analyses are provided.

### 2.4. Survival Analysis

Survival analysis was performed on both the TCGA and CGGA cohorts using a Kaplan–Meier curve and the Cox proportional hazard model. The analyses were executed in R v4.3.2 using the ‘survival’ (v3.8-3) [25] and ‘survminer’ (v0.5.1) [26] packages. Patients were dichotomised into low- and high-expression groups, using an optimal cut-off value computed by the ‘maxstat’ (v0.7-26) package [27]. This method evaluates all possible thresholds and selects the one that provides the greatest separation of survival outcomes based on log-rank statistics, ensuring that the chosen cut-off is both statistically and clinically meaningful. The cut-off value for each gene can be found in Tables S6–S9, Supplementary File S1.

Transcript per million (TPM) values were used for the TCGA dataset, while variance-stabilised transformed (VST) values were used for the CGGA dataset. The overall survival probabilities of the query genes were evaluated using Kaplan–Meier curves (log-rank *p*-values ≤ 0.05 denotes significance), while their prognostic values were assessed using the Cox proportional hazard model by calculating the hazard ratio (HR). Hazard ratios (HR) < 1 indicated favourable prognostic markers and HR > 1 indicated adverse prognostic markers (*p* < 0.05). Genes meeting these significance thresholds in both analyses were considered prognostic. The prognostic value of each NMRG was evaluated independently in the TCGA and CGGA cohorts, and only those showing consistent associations across both datasets were retained to construct the 10-NMRG signature.

### 2.5. Computation of the 10-NMRG Signature Prognostic Index

A total of 10 NMRGs were obtained from the survival analysis to compute the prognostic index, using the formula: $\sum_{i}Expression\left({Gene}_{i}\right)*\;{Gene\;Coefficient}_{i}$. The TCGA cohort was randomly divided into a training set (n = 83) and a validation set (n = 57) to enable independent evaluation of the risk model’s performance prior to validation in the complete TCGA and CGGA cohorts. The prognostic index was used to stratify GBM patients into high- and low-risk score groups based on the log-rank test. The survival probability between the two groups was then estimated using Kaplan–Meier plots for both the training and validation sets. The predictive accuracy of the gene signature was assessed using time-dependent ROC curve analyses, via the survivalROC (v1.0.3.1) R package. The prognostic performance of the 10-NMRG signature was assessed using the multivariate Cox regression analysis in the complete TCGA and CGGA cohorts. To account for potential clinical confounders, the available covariates, including age, gender, molecular subtype, chemotherapy, radiotherapy, and MGMT methylation status, were incorporated into the analysis.

### 2.6. Gene Set Enrichment Analysis (GSEA)

GSEA was performed among TCGA GBM patients that were stratified into high- and low-risk score groups by the 10-NMRG prognostic index [28]. The median of ratios normalised counts from DESeq2 were input into the GSEA v4.3.2 software, with the Molecular Signatures Database (MSigDB), hallmark gene sets (H) and curated gene sets (C2) KEGG_MEDICUS and Reactome subset of canonical pathways, being used for the analysis [29]. The permutation type was based on phenotype, with a total of 1000 iterations. A gene set was considered significantly enriched when the nominal (NOM) *p*-value < 0.01 and the false discovery rate (FDR) *q*-value < 0.05.

### 2.7. Active-Subnetwork-Oriented Enrichment Analysis (ASOEA)

ASOEA was performed using DEGs from the high- and low-risk score groups of the TCGA GBM cohort, as stratified by the 10-NMRG signature prognostic index. This was made possible using the R package ‘pathfindR’ (v2.6.0) (https://github.com/egeulgen/pathfindR) (accessed on 4 September 2023) [30]. Enrichment analysis performed through ‘pathfindR’ integrates the differential expression gene information to produce interaction information through protein–protein interaction networks via active subnetwork identification, which are subsequently fed into pathway/gene set annotation enrichment over 10 iterations.

### 2.8. Compositional Proportionality Analysis

Proportionality analyses between the ten genes of interest and various genes involved in GBM progression were carried out using the ‘propr’ package (v5.1.7) in R v4.3.2 (https://github.com/tpq/propr) (accessed on 11 September 2023) [31] on the raw expression count for GBM patients from TCGA. To overcome compositional data analyses’ inability to compute for zeros, all zeros were replaced by a Bayesian multiplicative replacement strategy that preserves the ratios between the non-zero components [32] using the zCompositions package (v1.5.0-5; cmultRepl function) [33]. Feature coordination between the various genes was calculated using the “ρ” measure. *p*-values are not calculated for proportionality; instead, the FDR was permuted for a given cut-off. To achieve an FDR of below 5%, we selected a cut-off of Ρρ > 0.25. The proportionality matrices were illustrated using the ‘corrplot’ (v0.95) package.

## 3. Results

### 3.1. Identification of deNMRGs in GBM Patient Samples

To identify neuromodulation-related genes (NMRGs) potentially implicated in GBM pathogenesis, differential expression analyses between GBM and normal brain tissues were independently performed across four RNA-Seq datasets (TCGA, CGGA, GSE147352 and GSE165595), encompassing a total of 300 *IDH*-wildtype GBM samples. To ensure consistent dysregulation of the identified differentially expressed NMRGs (deNMRGs), only genes exhibiting concordant expression trends in at least two datasets were retained. This selection approach accounted for variations in sample size (GBM vs. normal brain) and sequencing platforms across datasets, ensuring analytical robustness without excluding biologically relevant deNMRGs associated with GBM. Among the 420 screened NMRGs, 272 deNMRGs were identified, with 86 upregulated and 186 downregulated in the GBM samples. The distributions of deNMRGs by their neuromodulation category is detailed in Table 2, with the complete deNMRGs lists provided in Tables S10 and S11, Supplementary File S1.

### 3.2. IGF2, RETN, EDNRB, C3AR1, CLCF1, NTRK1, OSMR, KCNN4, SLC18A3 and HTR7 Are the Common Prognostic Genes in TCGA and CGGA GBM Cohorts

**Figure 2:**
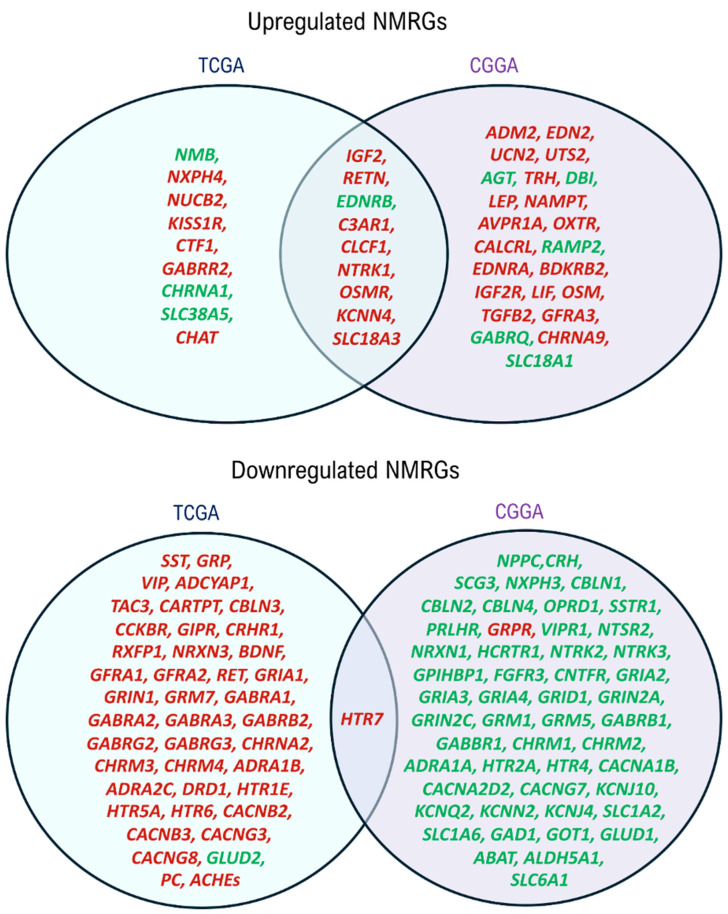
Venn Diagram showing common prognostic NMRGs between TCGA and CGGA cohorts. *IGF2*, *RETN*, *EDNRB*, *C3AR1*, *CLCF1*, *NTRK1*, *OSMR*, *KCNN4*, *SLC18A3* and *HTR7* predicted similar survival outcomes for GBM patients from the two cohorts. Genes predicting poorer prognoses are indicated in red, whereas those predicting better prognoses are denoted in green. The deNMRGs predicting for similar survival outcomes in GBM patients from both cohorts are presented in the intersection region.

**Figure 3:**
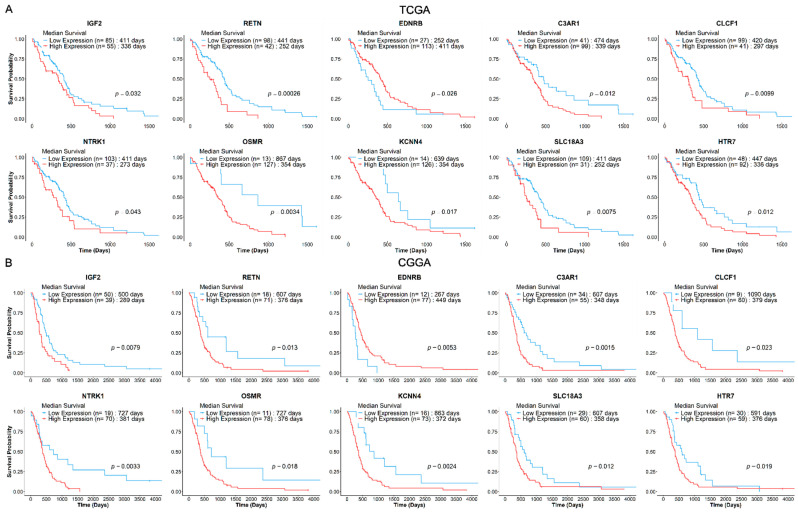
Kaplan–Meier survival curves for the 10 NMRGs from GBM (**A**) TCGA (n = 140) and (**B**) CGGA (n = 89) cohorts. GBM patients from the respective cohorts were stratified into low- (blue) and high-expression (red) groups, based on optimal cut-offs (log-rank *p* ≤ 0.05). The Kaplan–Meier survival curves showed that *IGF2*, *RETN*, *C3AR1*, *CLCF1*, *NTRK1*, *OSMR*, *KCNN4*, *SLC18A3* and *HTR7* consistently predict poorer survival, while *EDNRB* predicts a more favourable survival outcome in both cohorts. The patient group sizes and log-rank *p*-value for each curve are as shown.

**Figure 4:**
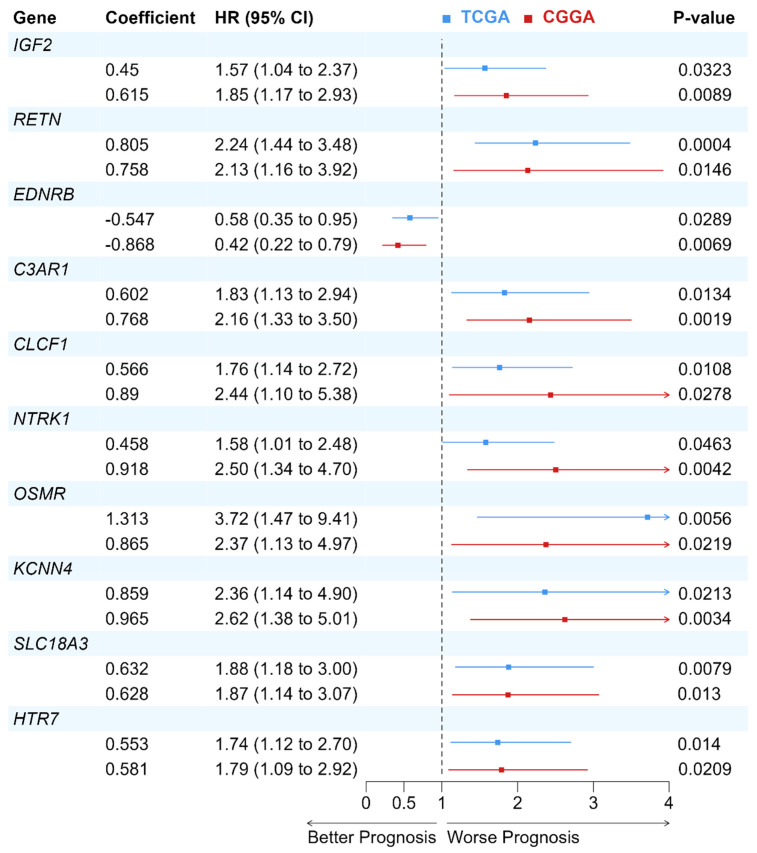
Forest plot of the Cox proportional hazard model for the 10-NMRG in GBM patients from TCGA and CGGA cohorts. The prognostic values of the 10 genes were assessed using the Cox proportional hazard model. Hazard ratio (HR) > 1 indicates a worse prognosis in GBM patients with higher gene expression, while HR < 1 implies a better prognosis in GBM patients with higher gene expression.

The prognostic values of identified deNMRGs were evaluated using Kaplan–Meier survival and univariate Cox regression analyses in the TCGA and CGGA GBM cohorts. The analysis revealed that among the 86 upregulated and 186 downregulated NMRGs, 18 upregulated and 42 downregulated genes in TCGA, along with 32 upregulated and 51 downregulated genes in CGGA, exhibited significant prognostic value for overall survival (OS) (Tables S6–S9, Supplementary File S1). In addition, comparison of prognostic deNMRGs between the TCGA and CGGA cohorts identified ten deNMRGs (*IGF2*, *RETN*, *EDNRB*, *C3AR1*, *CLCF1*, *NTRK1*, *OSMR*, *KCNN4*, *SLC18A3*, *HTR7*) that consistently predicted for similar survival outcomes in both cohorts (Figure 2). In the TCGA and CGGA cohorts, GBM with high expressions of *IGF2* (HR = 1.57; 1.85), *RETN* (2.24; 2.13), *C3AR1* (1.83; 2.16), *CLCF1* (1.76; 2.44), *NTRK1* (1.58; 2.50), *OSMR* (3.72; 2.37), *KCNN4* (2.36; 2.62), *SLC18A3* (1.88; 1.87) or *HTR7* (1.74; 1.79) had significant shorter OS, indicating predictions for a worse prognosis. Meanwhile, high *EDNRB* expression was associated with longer OS (HR = 0.579; 0.420) and better outcomes in the TCCGA and CGGA cohorts (Figure 3 and Figure 4).

### 3.3. A 10-NMRG Signature Forms a Risk Score Model That Predicts Prognosis in TCGA and CGGA GBM Cohorts

**Figure 5:**
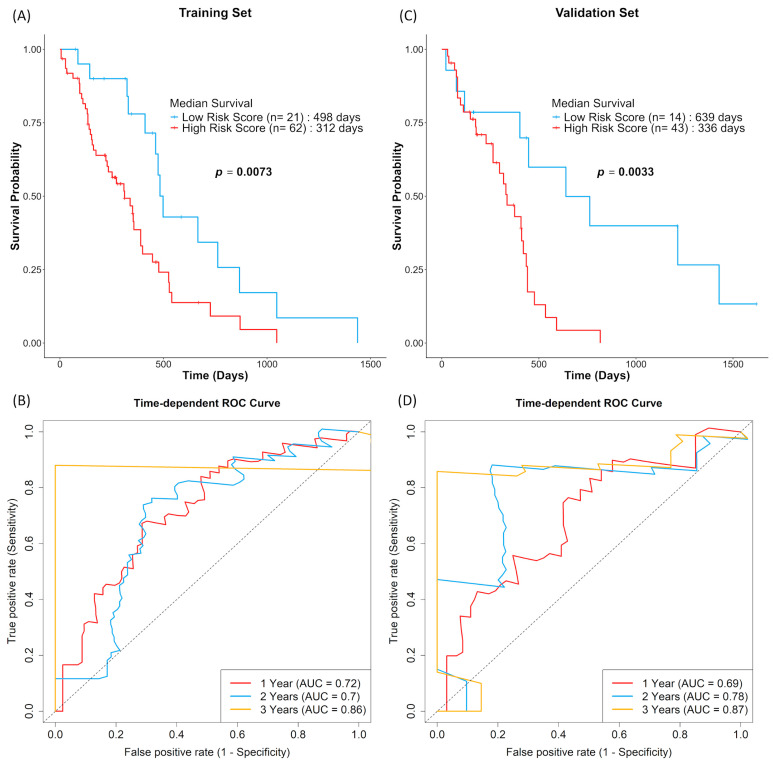
GBM patients with high 10-NMRG signature risk scores were associated with worse survival outcomes. GBM patients in the training (**A**,**B**; n = 83) and validation sets (**C**,**D**; n = 57) were stratified into low-risk score (blue) and high-risk score (red) groups based on log-rank tests. The overall survival probabilities of the 10-NMRG signature were evaluated using Kaplan–Meier curves, with the prognostic capacity evaluation of the gene signature being evaluated using time-dependent ROC curves.

A 10-NMRG signature comprising *IGF2*, *RETN*, *EDNRB*, *C3AR1*, *CLCF1*, *NTRK1*, *OSMR*, *KCNN4*, *SLC18A3* and *HTR7* was constructed. By splitting the TCGA dataset into a training (n = 83) and a validation set (n = 57), the predictive performance of this ten-gene signature in TCGA dataset was evaluated using Kaplan–Meier survival analysis. Both sets showed similar results, where high-risk score GBM patients showed decreased OS (Figure 5A,C). The prognostic performance of the 10-NMRG signature in evaluating 1-, 2- and 3-year OS rates were also favourable, where the time-dependent ROC analysis (Figure 5B,D) returned AUC values above 0.7 across both datasets.

**Figure 6:**
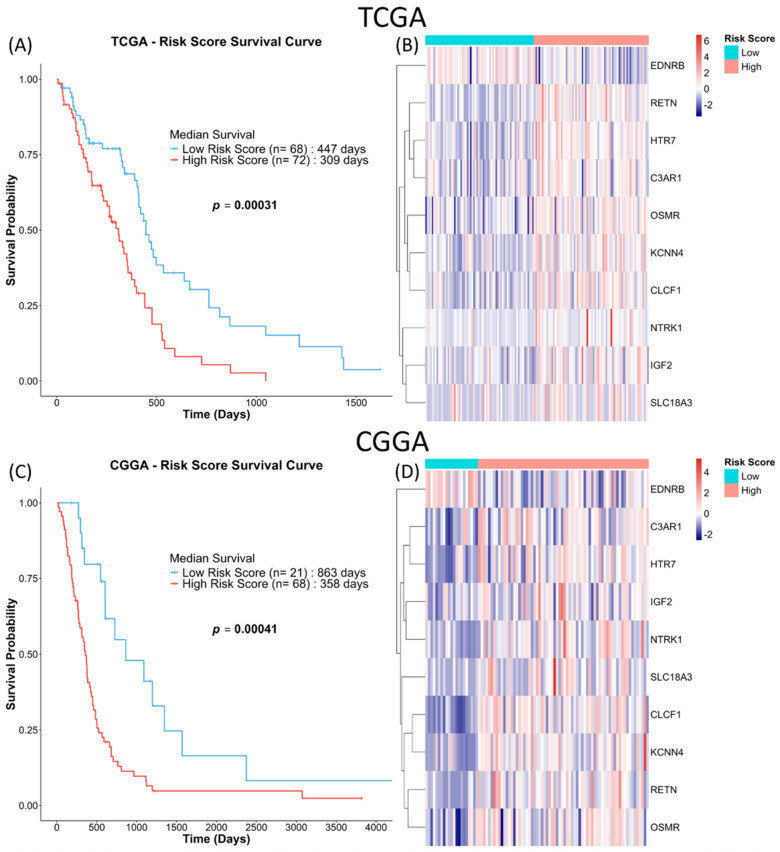
10-NMRG signature risk scores are consistently associated with worse survival outcomes. GBM patients in the full TCGA and CGGA cohorts were similarly stratified into low-risk score (blue) and high-risk score (red) groups based on the log-rank tests. Kaplan–Meier survival analysis was used to evaluate the prognosis of the patients stratified by the gene signature in TCGA (**A**) and CGGA (**C**) cohorts. (**B**,**D**) Heatmaps of the expression of the 10-NMRG in the GBM samples.

The observation was similar when the 10-NMRG signature was evaluated on the entire TCGA GBM dataset, where high-risk score GBM had approximately 4.5 months shorter OS (Figure 6A). The accuracy of the gene signature in predicting OS was further validated in the CGGA cohort, which consisted of a different population. Similarly to the TCGA cohort, the CGGA high-risk score GBM had approximately 16 months shorter OS (Figure 6C). Lastly, multivariate Cox regression analysis (Table 3) showed that the gene signature can act as an independent prognostic factor among common GBM prognostic factors (age, gender, subtypes, chemo- and radio-therapy statuses and *MGMT* methylation status) in both cohorts.

### 3.4. GBM with High 10-NMRG Signature Risk Score Is Associated with Immunomodulation and Tumour-Associated Hallmarks

To understand the molecular features of high-risk score GBM stratified by the 10-NMRG signature, GSEA was conducted to analyse the transcriptomic profiles of a GBM tumour in the TCGA cohort. GSEA using the MSigDB hallmark gene sets revealed significant gene enrichment associated with immunomodulation (TNFα signalling via NF-κB, IL6-JAK-STAT3 signalling, inflammatory response, complement, IL2-STAT5 signalling) and tumour-associated (epithelial–mesenchymal transition, hypoxia, KRAS signalling activation, apical junction, early response to oestrogen, angiogenesis) hallmarks in high-risk score GBM. Furthermore, GSEA using KEGG MEDICUS and Reactome pathway gene sets also identified enrichment of the pro-inflammatory immune response (IL6, IL2 and hormone-like cytokines-family-mediated JAK-STAT signalling pathways, TNF receptor superfamily TNFSF members mediating non-canonical NF-κB pathway and complement cascade), anti-inflammatory immune response (IL4 and IL13 signalling), integrins-mediated cell–matrix interaction and cytoskeleton remodelling (ITGA/B-RHOGAP or RHOGEF- modulated RHOA signalling, ITGA/B-FAK-modulated RAC and CDC42 signalling and ITGA/B-talin-vinculin signalling) and extracellular matrix (ECM) remodelling (degradation of ECM, activation of matrix metalloproteinase (MMPs) and collagen degradation) gene sets in high-risk score GBM (Table 4; Figures S1–S3, Supplementary File S2).

Next, DEGs were identified in high-risk against low-risk score GBM from TCGA cohort. Using pathfindR, ASOEA was conducted to identify gene subsets, which form functional protein interactions in mediating immunomodulation and tumour-associated pathways in high-risk score GBM. Significant upregulation of interleukin, C-C motif chemokine ligand (CCL), C-X-C motif chemokine ligand (CXCL) and complement system genes were observed in the high-risk group (Table S12, Supplementary File S1). These genes were significantly enriched in immunomodulation-related GO biological process terms, including positive regulation of cytokine, IL-6, IL-2 and TNF production, positive regulation of NF-kB signalling and neutrophil chemotaxis (Table 5). In addition, the high-risk score GBM also showed significantly higher expressions of integrins, EMT transcription factors, ECM components and matrix proteases genes, which regulate GO biological processes related to cell adhesion, cell migration, cell polarity, cytoskeletal dynamics, ECM remodelling and angiogenesis (Table 5; Table S12, Supplementary File S1).

Moreover, KEGG and Reactome pathway enrichment analyses further supported that upregulated genes in the high-risk GBM formed functional networks modulating three key categories: (i) immunomodulatory pathways (e.g., JAK-STAT, chemokine and TNF signalling pathway), (ii) oncogenic signalling pathways (e.g., oestrogen, Rap1, Ras signalling pathways) and (iii) cell–matrix interactions-related processes (e.g., ECM-receptor interaction, cell surface, laminin interactions, focal adhesion, regulation of actin cytoskeleton, degradation of ECM) (Table 5; Table S12, Supplementary File S1).

### 3.5. C3AR1, CLCF1, OSMR, KCNN4 and HTR7 Are Positively Correlated with Immune Activator and Immune Suppressor Genes in GBM

**Figure 7:**
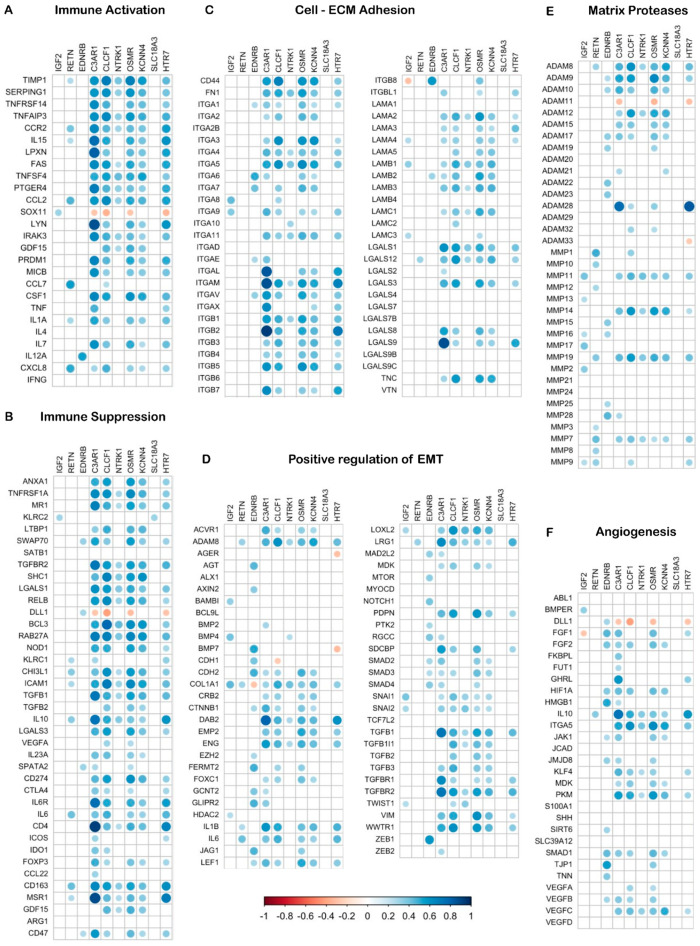
Compositional proportionality analysis reveals close association between the 10-NMRGs and cancer hallmark-related genes. The heatmap displays proportional relationships between the 10-NMRGs and immune response, EMT, cell–matrix adhesion, EC remodelling and angiogenesis genes. Dot size and colour intensity reflect proportionality strength (blue = direct; red = inverse), while blanks indicate non-significant relationships.

Next, compositional proportionality analysis revealed relationships between the 10-NMRGs and genes involved in immune responses, EMT, cell–ECM interactions, ECM dynamics, and angiogenesis in GBM. Among the 10-NMRGs, *C3AR1*, *CLCF1*, *OSMR*, *KCNN4* and *HTR7* showed strong positive proportionality with many immune activator (Figure 7A) and suppressor genes (Figure 7B), while moderate positive correlations were observed with the cell–ECM adhesion genes, especially integrins (Figure 7C). In contrast, *C3AR1*, *CLCF1*, *OSMR* and *KCNN4* showed weak positive proportionality with EMT regulators, matrix proteases and angiogenesis genes (Figure 7D–F).

### 3.6. GBM Mesenchymal Subtype Showed High Expressions of RETN, C3AR1, CLCF1, NTRK1, OSMR, KCNN4, and HTR7 Compared to Classical and Proneural Subtypes

**Figure 8:**
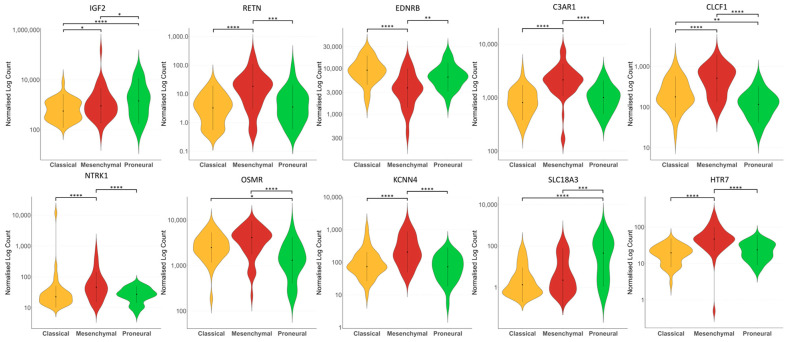
Expression of 10-NMRG in different GBM subtypes. The violin plots showed the different expression levels of *IGF2*, *RETN*, *C3AR1*, *EDNRB*, *CLCF1*, *NTRK1*, *OSMR*, *KCNN4*, *SLC18A3* and *HTR7* in classical, mesenchymal and proneural subtypes of GBM from TCGA cohort. * *p* < 0.05; ** *p* < 0.01; *** *p* < 0.001; **** *p* < 0.0001.

Comparison of the 10-NMRGs expression patterns across GBM subtypes revealed significant differences in their expression levels among the proneural, classical and mesenchymal subtypes. Notably, the mesenchymal subtype showed significantly higher expressions of *RETN*, *C3AR1*, *CLCF1*, *NTRK1*, *KCNN4* and *HTR7* compared to proneural and classical subtypes (all *p* < 0.0001, except RETN mesenchymal vs. proneural *p* < 0.001), while OSMR is significantly elevated in mesenchymal compared to the proneural subtype (*p* < 0.0001). Meanwhile, EDNRB was significantly downregulated in the mesenchymal versus the proneural (*p* < 0.01) and classical (*p* < 0.0001) subtypes (Figure 8).

## 4. Discussion

GBM tumours co-opt normal neuromodulatory pathways by aberrantly expressing neuromodulators, such as neuropeptides, neurotrophic factors, neurotransmitters and their cognate receptors, to drive progression [34,35]. While several neuromodulators are known to regulate GBM behaviour, the roles of many other neuromodulators remain unclear. In this study, systematic analysis of GBM gene expression profiles and clinical data identified a 10-NMRG signature that stratifies high-risk tumours and predicts poorer survival among GBM patients, through associations with immunomodulation and tumour-associated hallmarks.

In both the TCGA and CGGA GBM cohorts, high expressions of *IGF2*, *RETN*, *C3AR1*, *CLCF1*, *NTRK1*, *OSMR*, *KCNN4*, *SLC18A3* and *HTR7* predicted poorer prognoses, while *EDNRB* was correlated with a better outcome (Figure 3 and Figure 4). The resulting 10-NMRG signature effectively stratified the TCGA GBM patients into low- and high-risk score groups with distinct survival outcomes. This predictive model was validated in the CGGA cohort, demonstrating its robustness across Caucasian and Asian populations (Figure 6). When analysed in relation to different ages, sex, subtypes, treatment received and MGMT methylation status, the 10-NMRG signature was able to predict survival independently in both cohorts, signifying its potential as a feature-agnostic signature (Table 3).

Among the ten genes, *RETN* [36,37], *C3AR1* [38,39], *CLCF1* [40], *NTRK1* [41,42], *OSMR* [43], *KCNN4* [44,45] and *HTR7* [46] have established roles in cancer pathogenesis through regulating tumour growth, motility, angiogenesis, chemoresistance and immune evasion. In GBM, preclinical studies have demonstrated the oncogenic roles of *IGF2* [47], *EDNRB* [48,49], *C3AR1* [50,51], *CLCF1* [52], *NTRK1* [42,53], *OSMR* [54,55,56,57], *KCNN4* [58,59] and *SLC18A3* [6] in promoting proliferation, migration, invasion, EMT, chemoresistance, stemness and survival (Table 6). The roles of the remaining genes in GBM pathogenesis remain unreported. GSEA revealed that GBM tumours in the high-risk score group are associated with immunomodulation (TNFα signalling via NF-kB, IL6-JAK-STAT3 signalling, inflammatory response, complement, IL2-STAT5 signalling) and tumour-associated hallmarks (EMT, KRAS signalling activation, early response to oestrogen, angiogenesis) (Table 4; Figure S1, Supplementary File S2). These findings suggested the plausible molecular mechanisms linking the 10-NMRG high-risk score group to poorer prognosis in GBM.

The GBM tumour microenvironment is shaped by dynamic tumour–immune interactions that promote tumour progression through the complex interplay of inflammation and immune suppression [61,62,63]. The release of pro-inflammatory chemokines and cytokines, such as IL-1, IL-2, IL-6, CCL-2, CCL-8, TNF-α and TGF-β, facilitate the recruitment of brain-resident immune cells to the tumour site [64]. These factors not only promote GBM growth but also activate JAK-STAT and NF-κB pathways, which upregulate immune checkpoint molecules and immunosuppressive cytokines within the tumour microenvironment [64,65]. Cytokines including IL-6, IL-10, IL-11 and IL-24 can suppress immune effector cells, such as T cells, natural killer cells and dendritic cells, impairing the host’s anti-tumour response while facilitating the immune evasion of GBM cells [61,62,63]. Consistent with the existing literature, our analysis revealed that the high-risk score group expressed significantly higher levels of interleukins, chemokines and complement system genes involved in modulating pro-inflammatory (IL-2, IL-6, TNFα signalling pathways) and anti-inflammatory (IL-4, IL-6 and IL-13 signalling pathways) immune response, complement cascades activation, JAK-STAT and NF-κB pathways (Table 4 and Table 5; Table S12, Supplementary File S1).

Moreover, a strong positive correlation was observed between the expression of *C3AR1*, *CLCF1*, *OSMR*, *KCNN4* and *HTR7* genes with the immune activator (e.g., *IL1A*, *IL15*, *CCL2*) and suppressor (e.g., *TGFB1*, *IL6*, *IL10*) genes (Figure 7A,B), suggesting a possible co-expression and/or co-regulation of these five genes with the immune-related genes in GBM. To date, the understanding of the roles of NMRGs in immunomodulation remains limited, with preliminary preclinical findings suggesting the involvement of EDNRB [49] and C3AR1 [51] in modulating immune escape, and immune cells infiltration to tumour microenvironment in GBM. The strong association of heightened immune and inflammatory responses within the tumour site in the high-risk score GBM, shown in our analysis, suggests that dysregulation in host anti-tumour immune response might contribute to the poor prognosis predicted by the 10-NMRG signature.

In addition, high-risk score GBM also expressed significantly higher levels of integrins, EMT transcription factors, ECM components and matrix protease genes. ASOEA revealed that these genes are involved in regulating integrins-mediated cell–matrix interaction, RHO family GTPases- and FAK-signalling-induced cytoskeleton reorganisation, ECM remodelling and angiogenesis (Table 5; Table S12, Supplementary File S1), all of which are crucial for tumour cell migration and invasion. Among them, *C3AR1*, *CLCF1*, *OSMR*, *KCNN4* and *HTR7* were also positively correlated with several genes related to cell–matrix adhesion (e.g., *FN1*, *ITGA3*, *ITGA5*, *ITGB1*), EMT (e.g., *TGFB1*, *TGFBR2*, *SNAI1*, *SNAI2*, *VIM*) and matrix remodelling (e.g., *ADAM8*, *ADAM9*, *MMP14*, *MMP19*) in GBM (Figure 7C–E). Altogether, the current findings suggest a possible connection between high expressions of the 10-NMRGs with increased modulation of the cell–matrix interaction, cytoskeleton reorganisation and ECM degradation in the high-risk score GBM, through the modulation of the listed genes. This may further enhance the invasion potential and aggressiveness of high-risk score GBM, leading to shorter OS in the patients.

The poor prognosis of the mesenchymal subtype GBM is known to be associated with their mesenchymal differentiation, angiogenesis, pro-inflammatory and immune suppression gene signatures [66,67]. While most of the genes in the 10-NMRG signature (*RETN*, *C3AR1*, *CLCF1*, *NTRK1*, *OSMR*, *KCNN4* and *HTR7*) are individually linked with poor prognosis, they also exhibited a relatively higher expression in the mesenchymal subtype compared to the classical and proneural subtypes (Figure 8). It is noteworthy that 50% of the high-risk score patients were classified as a mesenchymal subtype. Furthermore, three-quarters of these mesenchymal subtype GBM patients were also stratified as high-risk, based on the 10-NMRG signature. While the current findings suggest a link between 10-NMRG signature and mesenchymal subtype, a strong association between increased inflammatory, immune response and tumour-promoting hallmarks are observed in the high-risk score GBM. Notably, hyperactivation of immune-related pathways, including JAK-STAT signalling, NF-kB signalling and complement system, are all implicated in mediating EMT, tumour angiogenesis and production of MMPs in creating a proteolytic environment that favours GBM invasion [64,65,68]. This dual interplay of immunomodulation suggests a multifaceted microenvironment in the high-risk score GBM that facilitates tumour immune evasion and tumour invasion. Together, these factors collectively contribute to a more aggressive phenotype in GBM, which results in poor survival in the high-risk score group.

The strong association between high-risk score GBM with immunomodulation and tumour-associated hallmarks has provided insight into the clinically relevant mechanisms that contribute to the poor prognosis predicted by the 10-NMRG signature. Beyond their established neuromodulatory roles in neurons and glial cells [69,70,71], the current findings indicated that neuromodulators, including neuropeptides, neurotrophic factors, and neurotransmitters, could potentially drive GBM progression by altering immune response and activating oncogenic pathways. These findings also grant future preclinical research with elucidating the biological functions of the clinically significant neuromodulators in GBM pathogenesis, especially their roles in modulating tumour–immune dynamics in facilitating GBM progression and invasion.

## 5. Conclusions

This study established a clinically significant 10-NMRG signature comprising neuropeptides (*IGF2*, *RETN*), neuropeptide receptors (*EDNRB*, *C3AR1*), neurotrophic factor (*CLCF1*), neurotrophic factor receptor (*NTRK1*, *OSMR*), neurotransmitter receptor (*KCNN4*, *HTR7*) and neurotransmitter system-related (*SLC18A3*) genes that identifies high-risk GBM with immunomodulation and tumour-associated hallmarks. The risk-scoring model effectively stratifies high-risk patients across Caucasian and Asian populations, suggesting potential broader clinical application for treatment stratification and survival prediction. Moving forward, further research is needed to clarify their clinical significance and develop targeted therapies, especially for high-risk patients who may benefit from intensified monitoring or treatment.

## Figures and Tables

**Table 1:** Number of samples in each RNA-Seq cohort.

Dataset	Normal Brain	GBM	Paired/Unpaired
TCGA-GTEx	211	140	Unpaired
CGGA	20	89	Unpaired
GSE147352	15	56	Unpaired
GSE165595	15	15	Paired

**Table 2:** Summary of differentially expressed NMRGs identified in GBM patient samples vs. normal brain tissues.

Genes	Input Gene List	Number of DEGs	Number of Upregulated Genes	Number of Downregulated Genes
Neuropeptides	97	50	25	25
Neuropeptide receptors	97	60	22	38
Neurotrophic factors	26	14	9	5
Neurotrophic factor receptors	22	16	9	7
Neurotransmitter receptors	131	99	12	87
Neurotransmitter system-related	47	33	9	24

**Table 3:** Multivariate Cox Regression Analysis of 10-NMRG signature and other common GBM prognostic clinical factors.

Variable	Characteristics	Univariable Cox			Multivariable Cox		
		HR	95% CI	*p*-Value	HR	95% CI	*p*-Value
TCGA							
Risk Score	High vs. Low	2.15	1.41–3.27	<0.001	2.86	1.36–5.98	0.005
Age	Continuous	1.03	1.01–1.05	0.003	1.02	0.99–1.05	0.196
Gender	Male vs. Female	0.85	0.57–1.28	0.444	1.14	0.55–2.36	0.716
Radiation	Yes vs. No	0.21	0.13–0.34	<0.001	0.14	0.06–0.36	<0.001
Chemotherapy	Yes vs. No	0.40	0.26–0.62	<0.001	1.88	0.72–4.87	0.196
MGMT	Methylated vs. Unmethylated	0.67	0.41–1.10	0.112	0.63	0.30–1.31	0.219
Subtype	Mesenchymal vs. Classical	1.12	0.66–1.92	0.675	0.92	0.44–1.92	0.280
	Proneural vs. Classical	1.37	0.76–2.46	0.292	1.02	0.4–2.6	0.96
CGGA							
Risk Score	High vs. Low	2.77	1.54–4.99	<0.001	2.41	1.27–4.56	0.007
Age	Continuous	1.02	1.00–1.04	0.069	1.02	1.00–1.04	0.047
Gender	Male vs. Female	1.27	0.79–2.06	0.321	1.48	0.85–2.56	0.164
Radiation	Yes vs. No	0.86	0.47–1.58	0.634	0.83	0.49–1.41	0.490
Chemotherapy	Yes vs. No	0.33	0.19–0.57	<0.001	0.57	0.29–1.12	0.100
MGMT	Methylated vs. Unmethylated	0.80	0.50–1.28	0.347	0.44	0.25–0.78	0.005

**Table 4:** Enriched GSEA terms identified in high 10-NMRG risk score GBM using MSigDB hallmark, KEGG_MEDICUS and Reactome gene sets.

Hallmarks/Pathways	NES	NOM *p*-Val	FDR *q*-Val
**MSigDB Hallmark**			
TNFA_SIGNALING_VIA_NFKB	2.03	<0.0001	0.0053
EPITHELIAL_MESENCHYMAL_TRANSITION	2.05	0.0020	0.0076
COAGULATION	1.99	<0.0001	0.0076
HYPOXIA	1.97	0.0020	0.0077
IL6_JAK_STAT3_SIGNALING	1.91	0.0079	0.0093
INFLAMMATORY_RESPONSE	1.94	0.0039	0.0095
KRAS_SIGNALING_UP	1.86	0.0019	0.0128
APICAL_JUNCTION	1.87	<0.0001	0.0138
ESTROGEN_RESPONSE_EARLY	1.81	<0.0001	0.0186
COMPLEMENT	1.81	0.0039	0.0203
IL2_STAT5_SIGNALING	1.79	0.0098	0.0213
ANGIOGENESIS	1.71	0.0154	0.0435
**KEGG_MEDICUS**			
IL6_FAMILY_TO_JAK_STAT_SIGNALING_PATHWAY	1.97	0.0020	0.0177
ITGA_B_RHOGAP_RHOA_SIGNALING_PATHWAY	1.95	0.0020	0.0182
ITGA_B_RHOGEF_RHOA_SIGNALING_PATHWAY	1.97	0.0020	0.0231
ITGA_B_FAK_RAC_SIGNALING_PATHWAY	1.90	0.0040	0.0281
IL2_FAMILY_TO_JAK_STAT_SIGNALING_PATHWAY	1.87	0.0038	0.0299
ITGA_B_FAK_CDC42_SIGNALING_PATHWAY	1.91	0.0040	0.0309
HORMONE_LIKE_CYTOKINE_TO_JAK_STAT_SIGNALING_PATHWAY	1.87	0.0060	0.0334
ITGA_B_RHOG_RAC_SIGNALING_PATHWAY	1.87	0.0079	0.0338
ITGA_B_TALIN_VINCULIN_SIGNALING_PATHWAY	1.98	0.0040	0.0461
**Reactome**			
CELL_SURFACE_INTERACTIONS_AT_THE_VASCULAR_WALL	1.98	0.0019	0.0343
TNF_RECEPTOR_SUPERFAMILY_TNFSF_MEMBERS_ MEDIATING_NON_CANONICAL_NF_KB_PATHWAY	1.98	0.0020	0.0379
DEGRADATION_OF_THE_EXTRACELLULAR_MATRIX	1.96	<0.0001	0.0388
ACTIVATION_OF_MATRIX_METALLOPROTEINASES	1.99	<0.0001	0.0391
COMPLEMENT_CASCADE	1.95	0.0020	0.0415
COLLAGEN_DEGRADATION	1.94	<0.0001	0.0450
NEGATIVE_REGULATION_OF_TCF_DEPENDENT_ SIGNALING_BY_WNT_LIGAND_ANTAGONISTS	1.92	<0.0001	0.0467
ANTIMICROBIAL_PEPTIDES	1.93	<0.0001	0.0472
INTERLEUKIN_4_AND_INTERLEUKIN_13_SIGNALING	1.99	0.0020	0.0473

Abbreviations: NES, normalised enrichment score; NOM *p*-val, normalised *p* value; FDR *q*-val, false discovery rate (corrected by Benjamini–Hochberg procedure) *q* value.

**Table 5:** Enriched ASOEA terms mapped by the differentially expressed genes identified in high 10-NMRG risk score GBMs.

Term ID	GO Biological Process	Highest *p* Value
**Immunomodulation-related**		
GO:0042110	T cell activation	3.16 × 10^−2^
GO:0050853	B cell receptor signalling pathway	2.02 × 10^−5^
GO:0007159	Leukocyte cell–cell adhesion	1.44 × 10^−2^
GO:0030217	T cell differentiation	6.30 × 10^−5^
GO:0001819	Positive regulation of cytokine production	7.93 × 10^−5^
GO:0002250	Adaptive immune response	9.31 × 10^−6^
GO:0043123	Positive regulation of I-kappaB kinase/NF-kappaB signalling	2.99 × 10^−5^
GO:0050901	Leukocyte tethering or rolling	2.10 × 10^−2^
GO:0032755	Positive regulation of interleukin-6 production	2.77 × 10^−3^
GO:0032760	Positive regulation of tumour necrosis factor production	2.18 × 10^−4^
GO:0019221	Cytokine-mediated signalling pathway	9.43 × 10^−4^
GO:0051092	Positive regulation of NF-kappaB transcription factor activity	2.55 × 10^−3^
GO:0031295	T cell costimulation	1.54 × 10^−2^
GO:0030593	Neutrophil chemotaxis	1.55 × 10^−2^
GO:0032743	Positive regulation of interleukin-2 production	4.76 × 10^−2^
GO:0045087	Innate immune response	2.19 × 10^−2^
**EMT/cell-ECM adhesion/matrix remodelling/angiogenesis-related**		
GO:0007229	Integrin-mediated signalling pathway	1.98 × 10^−9^
GO:0034113	Heterotypic cell–cell adhesion	4.90 × 10^−6^
GO:0071260	Cellular response to mechanical stimulus	7.10 × 10^−5^
GO:0030335	Positive regulation of cell migration	5.27 × 10^−6^
GO:0030199	Collagen fibril organisation	5.02 × 10^−4^
GO:0034446	Substrate adhesion-dependent cell spreading	2.28 × 10^−6^
GO:0007155	Cell adhesion	1.32 × 10^−4^
GO:0030574	Collagen catabolic process	3.62 × 10^−5^
GO:0045109	Intermediate filament organisation	1.61 × 10^−5^
GO:0070372	Regulation of ERK1 and ERK2 cascade	2.67 × 10^−5^
GO:0070374	Positive regulation of ERK1 and ERK2 cascade	2.81 × 10^−4^
GO:0022617	Extracellular matrix disassembly	1.81 × 10^−4^
GO:0014065	Phosphatidylinositol 3-kinase signalling	4.16 × 10^−4^
GO:0014068	Positive regulation of phosphatidylinositol 3-kinase signalling	1.20 × 10^−3^
GO:0045766	Positive regulation of angiogenesis	1.41 × 10^−5^
GO:0010575	Positive regulation of vascular endothelial growth factor production	1.20 × 10^−3^
**Term ID**	**KEGG Pathway**	**Highest** ***p* Value**
**Immunomodulation-related**		
hsa04630	JAK-STAT signalling pathway	9.61 × 10^−9^
hsa04613	Neutrophil extracellular trap formation	1.50 × 10^−15^
hsa04060	Cytokine–cytokine receptor interaction	1.93 × 10^−10^
hsa04659	Th17 cell differentiation	2.68 × 10^−4^
hsa04610	Complement and coagulation cascades	8.87 × 10^−10^
hsa04064	NF-kappa B signalling pathway	2.37 × 10^−10^
hsa04062	Chemokine signalling pathway	4.68 × 10^−3^
hsa04750	Inflammatory mediator regulation of TRP channels	1.99 × 10^−5^
hsa04670	Leukocyte transendothelial migration	7.53 × 10^−5^
hsa04668	TNF signalling pathway	1.01 × 10^−6^
hsa04662	B cell receptor signalling pathway	3.62 × 10^−6^
hsa04658	Th1 and Th2 cell differentiation	8.28 × 10^−3^
hsa04660	T cell receptor signalling pathway	6.01 × 10^−6^
**EMT/cell-ECM adhesion/matrix remodelling/angiogenesis-related**		
hsa04512	ECM-receptor interaction	1.18 × 10^−14^
hsa04510	Focal adhesion	3.01 × 10^−13^
hsa04915	Oestrogen signalling pathway	1.88 × 10^−9^
hsa04014	Ras signalling pathway	7.27 × 10^−6^
hsa05205	Proteoglycans in cancer	2.79 × 10^−8^
hsa04810	Regulation of actin cytoskeleton	5.78 × 10^−8^
hsa04010	MAPK signalling pathway	5.05 × 10^−7^
hsa04015	Rap1 signalling pathway	1.17 × 10^−3^
hsa04520	Adherens junction	2.77 × 10^−5^
**Term ID**	**Reactome Pathway**	**Highest** ***p* Value**
**Immunomodulation-related**		
R-HSA-173623	Classical antibody-mediated complement activation	1.14 × 10^−21^
R-HSA-166663	Initial triggering of complement	5.71 × 10^−22^
R-HSA-977606	Regulation of complement cascade	1.06 × 10^−21^
R-HSA-166786	Creation of C4 and C2 activators	1.22 × 10^−20^
R-HSA-166658	Complement cascade	7.87 × 10^−21^
R-HSA-5690714	CD22-mediated BCR regulation	1.51 × 10^−15^
R-HSA-983695	Antigen activates B cell receptor (BCR) leading to generation of second messengers	3.11 × 10^−15^
R-HSA-983705	Signalling by the B cell receptor (BCR)	3.17 × 10^−11^
R-HSA-5668541	TNFR2 non-canonical NF-kB pathway	5.49 × 10^−12^
R-HSA-451927	Interleukin-2 family signalling	4.84 × 10^−7^
R-HSA-512988	Interleukin-3, Interleukin-5 and GM-CSF signalling	4.84 × 10^−7^
R-HSA-6785807	Interleukin-4 and Interleukin-13 signalling	2.83 × 10^−8^
R-HSA-5676594	TNF receptor superfamily (TNFSF) members mediating non-canonical NF-kB pathway	1.16 × 10^−9^
R-HSA-1266695	Interleukin-7 signalling	2.04 × 10^−2^
**EMT/cell-ECM adhesion/matrix remodelling/angiogenesis-related**		
R-HSA-1474244	Extracellular matrix organisation	8.58 × 10^−21^
R-HSA-1474290	Collagen formation	6.02 × 10^−17^
R-HSA-202733	Cell surface interactions at the vascular wall	1.58 × 10^−13^
R-HSA-2219530	Constitutive signalling by aberrant PI3K in cancer	4.06 × 10^−11^
R-HSA-2022090	Assembly of collagen fibrils and other multimeric structures	5.64 × 10^−12^
R-HSA-2219528	PI3K/AKT signalling in cancer	8.74 × 10^−10^
R-HSA-6811558	PI5P, PP2A and IER3 regulate PI3K/AKT signalling	9.63 × 10^−10^
R-HSA-3000157	Laminin interactions	1.56 × 10^−14^
R-HSA-199418	Negative regulation of the PI3K/AKT network	1.85 × 10^−9^
R-HSA-9013149	RAC1 GTPase cycle	3.79 × 10^−5^
R-HSA-8874081	MET activates PTK2 signalling	8.36 × 10^−11^
R-HSA-216083	Integrin cell surface interactions	1.19 × 10^−10^
R-HSA-1474228	Degradation of the extracellular matrix	8.44 × 10^−10^
R-HSA-1257604	PIP3 activates AKT signalling	1.67 × 10^−5^

**Table 6:** Known oncogenic roles of prognostic genes in GBM.

Prognostic Genes	Oncogenic Roles in GBM	References
*IGF2*	↑ mRNA expression in GBM tumours → associated with shorter survival. Promote growth in GBM-derived neurosphere and tumour lacking *EGFR* amplification via IGF2-PIK3R3 signalling axis.	[47]
*RETN*	No known oncogenic functions in GBM.	-
*EDNRB*	↑ expression in glioma tissues and cells (C6, U251, U81). Maintain GSCs migration, undifferentiation and survival via EDN3-EDNRB signalling. Promotes proliferation and immune escape in glioma cells (U87).	[48,49]
*C3AR1*	↑ mRNA expression in *IDH*-wildtype GBM → associated with shorter survival. Hypoxia-induced C3a/C3AR1 signalling drives tumour growth and supports glioma cells (U3082MG) self-renewal → ↑ aggressiveness.	[50,51]
*CLCF1*	BRD4-activated CLCF1 plays multiple roles in cell proliferation, cell cycle progression, survival, migration and invasion capabilities of GBM cells (U87 and U251).	[52]
*NTRK1*	*NTRK1* fusion genes (e.g., *NFASC-NTRK1* and *BCAN-NTRK1*) are highly expressed in GBM → ↑ NTRK1-pathway activity. *NFASC-NTRK1* fusion gene confers tumorigenic functions → ↑ cell proliferation in vitro and tumour formation in vivo. Possible roles in GBM tumour initiation or maintenance in fusion-positive GBMs.	[42,53]
*OSMR*	OSMR-mediated feed forward signalling mechanism with EGFRvIII and STAT3 to drive cell proliferation and tumour growth in BTSCs and GBM cells in vitro and in vivo. Induced migration and invasion via OSM-STAT3 signalling → ↑ mesenchymal markers such as fibronectin and YKL-4 in GBM cells (LN-18, LN229, GBM primary culture G1)—associated with mesenchymal subtype GBM. ↑ regulation of cell invasion, angiogenesis, proliferation, growth and mesenchymal transition via ANXA2-OSMR signalling axis in GBM cell lines (MGG23, U87MG) and tumour-bearing mice. Confers resistance to IR via regulation of oxidative phosphorylation in GBM BTSCs.	[54,55,56,57]
*KCNN4*	KCNN4 (KCa3.1) channel activity → responsible for GBM tumour cell migration, invasion and infiltration in vivo. Contributes to TMZ resistance.	[58,59]
*SLC18A3*	Co-expression of functional choline transporters, choline acetyltransferase, and vesicular acetylcholine transporters (SLC18A3) required for ACh synthesis and release in GBM cells and xenograft cell lines (U251 and PDX14). ↑ invasive capability of GBM cells via AChR signalling-induced MMP-9 activity.	[6]
*HTR7*	Expression of functional 5-HT7 receptors in human glioblastoma cell lines. Oncogenic roles remain elusive.	[60]

Abbreviations: *EGFR*—Epidermal growth factor receptor; PIK3R3—Phosphoinositide-3-kinase regulatory subunit 3; GSCs—GBM stem cells; EDN3—Endothelin 3; BRD4—Bromodomain-containing protein 4; NFASC—Neurofascin; BCAN—Brevican; STAT3—Signal transducer and activator of transcription 3; OSM—Oncostatin M; YKL-40—Chitinase-3-like-1; ANXA2—Annexin A2; IR—Ionising radiation; BTSCs—Brain tumour-initiating stem cells; TMZ—Temozolomide, ACh—Acetylcholine; MMP-9—Matrix Metallopeptidase 9; ↑—Increase or increased.

## Data Availability

All alignment scripts and R scripts used in this paper can be accessed through the following link: https://github.com/drgellerbing/nmrg (accessed on 11 November 2023).

## Supplementary Materials

The following supporting information can be downloaded at: https://www.mdpi.com/article/10.3390/biomedicines13112640/s1. Supplementary File S1—Table S1: Demographic and clinical characteristics of GBM patients from TCGA and CGGA database; Table S2: List of neuropeptide and neuropeptide receptor genes included in differential expression analysis; Table S3: List of neurotrophic factor and neurotrophic factor receptor genes included in differential expression analysis; Table S4: List of neurotransmitter receptor genes included in differential expression analysis; Table S5: List of neurotransmitter system-related genes included in differential expression analysis; Table S6: List of upregulated NMRGs acting as a prognostic marker for GBM patients from TCGA cohort; Table S7: List of downregulated NMRGs acting as a prognostic marker for GBM patients from TCGA cohort; Table S8: List of upregulated NMRGs acting as a prognostic marker for GBM patients from CGGA cohort; Table S9: List of downregulated NMRGs acting as a prognostic marker for GBM patients from CGGA cohort; Table S10: List of upregulated NMRGs identified in GBM samples from TCGA, CGGA, GSE147352 and GSE165595 database; Table S11: List of downregulated NMRGs identified in GBM samples from TCGA, CGGA, GSE147352 and GSE165595 database; Table S12: Differentially expressed genes (DEGs) enriched in GO Biological Process, KEGG Pathway and Reactome Pathway terms in GBM with high 10-NMRGs risk score; Supplementary File S2—Figure S1: GSEA (MSigDB hallmark) of the 10-NMRG high-risk score group in GBM TCGA cohort; Figure S2: GSEA (KEGG_MEDICUS pathway) of the 10-NMRG high-risk score group in GBM TCGA cohort; Figure S3: GSEA (Reactome pathway) of the 10-NMRG high-risk score in GBM TCGA cohort.

## Abbreviations

The following abbreviations are used in this manuscript:

NMRGs	Neuromodulation-Related Genes
deNMRGs	Differentially Expressed NMRG
GBM	Glioblastoma, *IDH* Wildtype
TCGA	The Cancer Genome Atlas
CGGA	Chinese Glioma Genome Atlas
GEO	Gene Expression Omnibus
10-NMRG	10-Neuromodulation-Related Gene
GSEA	Gene Set Enrichment Analysis
ASOEA	Active Subnetwork-Oriented Enrichment Analysis
ECM	Extracellular Matrix
CCL	C-C Motif Chemokine Ligand
CXCL	C-X-C Motif Chemokine Ligand
GO	Gene Ontology
EMT	Epithelial-to-Mesenchymal Transition

## References

[1] Ostrom Q.T., Price M., Neff C., Cioffi G., Waite K.A., Kruchko C., Barnholtz-Sloan J.S. (2023). CBTRUS Statistical Report: Primary Brain and Other Central Nervous System Tumors Diagnosed in the United States in 2016—2020. Neuro-Oncology.

[2] Ostrom Q.T., Price M., Neff C., Cioffi G., Waite K.A., Kruchko C., Barnholtz-Sloan J.S. (2022). CBTRUS statistical report: Primary brain and other central nervous system tumors diagnosed in the United States in 2015–2019. Neuro-Oncology.

[3] Palma C., Nardelli F., Manzini S., Maggi C. (1999). Substance P activates responses correlated with tumour growth in human glioma cell lines bearing tachykinin NK1 receptors. Br. J. Cancer.

[4] Lawn S., Krishna N., Pisklakova A., Qu X., Fenstermacher D.A., Fournier M., Vrionis F.D., Tran N., Chan J.A., Kenchappa R.S. (2015). Neurotrophin signaling via TrkB and TrkC receptors promotes the growth of brain tumor-initiating cells. J. Biol. Chem..

[5] Pucci S., Fasoli F., Moretti M., Benfante R., Di Lascio S., Viani P., Daga A., Gordon T.J., McIntosh M., Zoli M. (2021). Choline and nicotine increase glioblastoma cell proliferation by binding and activating α7-and α9-containing nicotinic receptors. Pharmacol. Res..

[6] Thompson E.G., Sontheimer H. (2019). Acetylcholine receptor activation as a modulator of glioblastoma invasion. Cells.

[7] Piao Y., Lu L., De Groot J. (2009). AMPA receptors promote perivascular glioma invasion via β1 integrin–dependent adhesion to the extracellular matrix. Neuro-Oncology.

[8] Venkatesh H.S., Morishita W., Geraghty A.C., Silverbush D., Gillespie S.M., Arzt M., Tam L.T., Espenel C., Ponnuswami A., Ni L. (2019). Electrical and synaptic integration of glioma into neural circuits. Nature.

[9] Louis D.N., Perry A., Wesseling P., Brat D.J., Cree I.A., Figarella-Branger D., Hawkins C., Ng H., Pfister S.M., Reifenberger G. (2021). The 2021 WHO classification of tumors of the central nervous system: A summary. Neuro-Oncology.

[10] Huang T., Yang Y., Song X., Wan X., Wu B., Sastry N., Horbinski C.M., Zeng C., Tiek D., Goenka A. (2021). PRMT6 methylation of RCC1 regulates mitosis, tumorigenicity, and radiation response of glioblastoma stem cells. Mol. Cell.

[11] Xu L., Chen Y., Huang Y., Sandanaraj E., Yu J.S., Lin R.Y.-T., Dakle P., Ke X.-Y., Chong Y.K., Koh L. (2021). Topography of transcriptionally active chromatin in glioblastoma. Sci. Adv..

[12] Dobin A., Davis C.A., Schlesinger F., Drenkow J., Zaleski C., Jha S., Batut P., Chaisson M., Gingeras T.R. (2013). STAR: Ultrafast universal RNA-seq aligner. Bioinformatics.

[13] Li B., Dewey C.N. (2011). RSEM: Accurate transcript quantification from RNA-Seq data with or without a reference genome. BMC Bioinform..

[14] Goldman M.J., Craft B., Hastie M., Repečka K., McDade F., Kamath A., Banerjee A., Luo Y., Rogers D., Brooks A.N. (2020). Visualizing and interpreting cancer genomics data via the Xena platform. Nat. Biotechnol..

[15] Cerami E., Gao J., Dogrusoz U., Gross B.E., Sumer S.O., Aksoy B.A., Jacobsen A., Byrne C.J., Heuer M.L., Larsson E. (2012). The cBio cancer genomics portal: An open platform for exploring multidimensional cancer genomics data. Cancer Discov..

[16] Gao J., Aksoy B.A., Dogrusoz U., Dresdner G., Gross B., Sumer S.O., Sun Y., Jacobsen A., Sinha R., Larsson E. (2013). Integrative analysis of complex cancer genomics and clinical profiles using the cBioPortal. Sci. Signal..

[17] de Bruijn I., Kundra R., Mastrogiacomo B., Tran T.N., Sikina L., Mazor T., Li X., Ochoa A., Zhao G., Lai B. (2023). Analysis and Visualization of Longitudinal Genomic and Clinical Data from the AACR Project GENIE Biopharma Collaborative in cBioPortal. Cancer Res..

[18] Vivian J., Rao A.A., Nothaft F.A., Ketchum C., Armstrong J., Novak A., Pfeil J., Narkizian J., Deran A.D., Musselman-Brown A. (2017). Toil enables reproducible, open source, big biomedical data analyses. Nat. Biotechnol..

[19] Liu X., Li Y., Qian Z., Sun Z., Xu K., Wang K., Liu S., Fan X., Li S., Zhang Z. (2018). A radiomic signature as a non-invasive predictor of progression-free survival in patients with lower-grade gliomas. NeuroImage Clin..

[20] Wang Y., Qian T., You G., Peng X., Chen C., You Y., Yao K., Wu C., Ma J., Sha Z. (2015). Localizing seizure-susceptible brain regions associated with low-grade gliomas using voxel-based lesion-symptom mapping. Neuro-Oncology.

[21] Zhang K., Liu X., Li G., Chang X., Li S., Chen J., Zhao Z., Wang J., Jiang T., Chai R. (2022). Clinical management and survival outcomes of patients with different molecular subtypes of diffuse gliomas in China (2011–2017): A multicenter retrospective study from CGGA. Cancer Biol. Med..

[22] Zhao Z., Zhang K.-N., Wang Q., Li G., Zeng F., Zhang Y., Wu F., Chai R., Wang Z., Zhang C. (2021). Chinese Glioma Genome Atlas (CGGA): A comprehensive resource with functional genomic data from Chinese glioma patients. Genom. Proteom. Bioinform..

[23] Love M.I., Huber W., Anders S. (2014). Moderated estimation of fold change and dispersion for RNA-seq data with DESeq2. Genome Biol..

[24] Zhu A., Ibrahim J.G., Love M.I. (2019). Heavy-tailed prior distributions for sequence count data: Removing the noise and preserving large differences. Bioinformatics.

[25] Therneau T.M., Grambsch P.M., Therneau T.M., Grambsch P.M. (2000). The Cox Model.

[26] Kassambara A., Kosinski M., Biecek P., Fabian S. (2021). Survminer: Drawing Survival Curves Using’ggplot2′.

[27] Lausen B., Hothorn T., Bretz F., Schumacher M. (2004). Assessment of optimal selected prognostic factors. Biom. J. J. Math. Methods Biosci..

[28] Subramanian A., Tamayo P., Mootha V.K., Mukherjee S., Ebert B.L., Gillette M.A., Paulovich A., Pomeroy S.L., Golub T.R., Lander E.S. (2005). Gene set enrichment analysis: A knowledge-based approach for interpreting genome-wide expression profiles. Proc. Natl. Acad. Sci. USA.

[29] Liberzon A., Birger C., Thorvaldsdóttir H., Ghandi M., Mesirov J.P., Tamayo P. (2015). The molecular signatures database hallmark gene set collection. Cell Syst..

[30] Ulgen E., Ozisik O., Sezerman O.U. (2019). pathfindR: An R package for comprehensive identification of enriched pathways in omics data through active subnetworks. Front. Genet..

[31] Quinn T.P., Richardson M.F., Lovell D., Crowley T.M. (2017). propr: An R-package for identifying proportionally abundant features using compositional data analysis. Sci. Rep..

[32] Martín-Fernández J.A., Palarea-Albaladejo J., Olea R.A. (2011). Dealing with zeros. Compos. Data Anal. Theory Appl..

[33] Palarea-Albaladejo J., Martín-Fernández J.A. (2015). zCompositions—R package for multivariate imputation of left-censored data under a compositional approach. Chemom. Intell. Lab. Syst..

[34] Monje M. (2025). The neuroscience of brain cancers. Neuron.

[35] Winkler F., Venkatesh H.S., Amit M., Batchelor T., Demir I.E., Deneen B., Gutmann D.H., Hervey-Jumper S., Kuner T., Mabbott D. (2023). Cancer neuroscience: State of the field, emerging directions. Cell.

[36] Deb A., Deshmukh B., Ramteke P., Bhati F.K., Bhat M.K. (2021). Resistin: A journey from metabolism to cancer. Transl. Oncol..

[37] Sudan S.K., Deshmukh S.K., Poosarla T., Holliday N.P., Dyess D.L., Singh A.P., Singh S. (2020). Resistin: An inflammatory cytokine with multi-faceted roles in cancer. Biochim. Et Biophys. Acta (BBA)-Rev. Cancer.

[38] Huang J., Zhou L., Deng K. (2023). Prognostic marker C3AR1 is associated with ovarian cancer cell proliferation and immunosuppression in the tumor microenvironment. J. Ovarian Res..

[39] Shu C., Zha H., Long H., Wang X., Yang F., Gao J., Hu C., Zhou L., Guo B., Zhu B. (2020). C3a-C3aR signaling promotes breast cancer lung metastasis via modulating carcinoma associated fibroblasts. J. Exp. Clin. Cancer Res..

[40] Sims N.A. (2015). Cardiotrophin-like cytokine factor 1 (CLCF1) and neuropoietin (NP) signalling and their roles in development, adulthood, cancer and degenerative disorders. Cytokine Growth Factor Rev..

[41] Cocco E., Scaltriti M., Drilon A. (2018). NTRK fusion-positive cancers and TRK inhibitor therapy. Nat. Rev. Clin. Oncol..

[42] Kim J., Lee Y., Cho H.-J., Lee Y.-E., An J., Cho G.-H., Ko Y.-H., Joo K.M., Nam D.-H. (2014). NTRK1 fusion in glioblastoma multiforme. PLoS ONE.

[43] Masjedi A., Hajizadeh F., Dargani F.B., Beyzai B., Aksoun M., Hojjat-Farsangi M., Zekiy A., Jadidi-Niaragh F. (2021). Oncostatin M: A mysterious cytokine in cancers. Int. Immunopharmacol..

[44] Li Q.-T., Feng Y.-M., Ke Z.-H., Qiu M.-J., He X.-X., Wang M.-M., Li Y.-N., Xu J., Shi L.-L., Xiong Z.-F. (2020). KCNN4 promotes invasion and metastasis through the MAPK/ERK pathway in hepatocellular carcinoma. J. Investig. Med..

[45] Xu P., Mo X., Xia R., Jiang L., Zhang C., Xu H., Sun Q., Zhou G., Zhang Y., Wang Y. (2021). KCNN4 promotes the progression of lung adenocarcinoma by activating the AKT and ERK signaling pathways. Cancer Biomark..

[46] Karmakar S., Lal G. (2021). Role of serotonin receptor signaling in cancer cells and anti-tumor immunity. Theranostics.

[47] Soroceanu L., Kharbanda S., Chen R., Soriano R.H., Aldape K., Misra A., Zha J., Forrest W.F., Nigro J.M., Modrusan Z. (2007). Identification of IGF2 signaling through phosphoinositide-3-kinase regulatory subunit 3 as a growth-promoting axis in glioblastoma. Proc. Natl. Acad. Sci. USA.

[48] Liu Y., Ye F., Yamada K., Tso J.L., Zhang Y., Nguyen D.H., Dong Q., Soto H., Choe J., Dembo A. (2011). Autocrine endothelin-3/endothelin receptor B signaling maintains cellular and molecular properties of glioblastoma stem cells. Mol. Cancer Res..

[49] Pan D.-s., Feng S.-z., Cao P., Li J.-j. (2018). Endothelin B receptor promotes the proliferation and immune escape of malignant gliomas. Artif. Cells Nanomed. Biotechnol..

[50] Rosberg R., Smolag K.I., Sjolund J., Johansson E., Bergelin C., Wahlden J., Pantazopoulou V., Ceberg C., Pietras K., Blom A.M. (2024). Hypoxia-induced Complement Component 3 Promotes Aggressive Tumor Growth in the Glioblastoma Microenvironment. JCI Insight.

[51] Ah-Pine F., Malaterre-Septembre A., Bedoui Y., Khettab M., Neal J.W., Freppel S., Gasque P. (2023). Complement activation and up-regulated expression of anaphylatoxin C3a/C3aR in glioblastoma: Deciphering the links with TGF-β and VEGF. Cancers.

[52] Shen S.-H., Guo J.-F., Huang J., Zhang Q., Cui Y. (2022). Bromodomain-containing protein 4 activates cardiotrophin-like cytokine factor 1, an unfavorable prognostic biomarker, and promotes glioblastoma in vitro. Ann. Transl. Med..

[53] Torre M., Vasudevaraja V., Serrano J., DeLorenzo M., Malinowski S., Blandin A.-F., Pages M., Ligon A.H., Dong F., Meredith D.M. (2020). Molecular and clinicopathologic features of gliomas harboring NTRK fusions. Acta Neuropathol. Commun..

[54] Matsumoto Y., Ichikawa T., Kurozumi K., Otani Y., Fujimura A., Fujii K., Tomita Y., Hattori Y., Uneda A., Tsuboi N. (2020). Annexin A2–STAT3–Oncostatin M receptor axis drives phenotypic and mesenchymal changes in glioblastoma. Acta Neuropathol. Commun..

[55] Natesh K., Bhosale D., Desai A., Chandrika G., Pujari R., Jagtap J., Chugh A., Ranade D., Shastry P. (2015). Oncostatin-M differentially regulates mesenchymal and proneural signature genes in gliomas via STAT3 signaling. Neoplasia.

[56] Sharanek A., Burban A., Laaper M., Heckel E., Joyal J.-S., Soleimani V.D., Jahani-Asl A. (2020). OSMR controls glioma stem cell respiration and confers resistance of glioblastoma to ionizing radiation. Nat. Commun..

[57] Jahani-Asl A., Yin H., Soleimani V.D., Haque T., Luchman H.A., Chang N.C., Sincennes M.-C., Puram S.V., Scott A.M., Lorimer I.A.J. (2016). Control of glioblastoma tumorigenesis by feed-forward cytokine signaling. Nat. Neurosci..

[58] D’Alessandro G., Catalano M., Sciaccaluga M., Chece G., Cipriani R., Rosito M., Grimaldi A., Lauro C., Cantore G., Santoro A. (2013). KCa3.1 channels are involved in the infiltrative behavior of glioblastoma in vivo. Cell Death Dis..

[59] Alessandro G., Grimaldi A., Chece G., Porzia A., Esposito V., Santoro A., Salvati M., Mainiero F., Ragozzino D., Di Angelantonio S. (2016). KCa3.1 channel inhibition sensitizes malignant gliomas to temozolomide treatment. Oncotarget.

[60] Mahé C., Bernhard M., Bobirnac I., Keser C., Loetscher E., Feuerbach D., Dev K.K., Schoeffter P. (2004). Functional expression of the serotonin 5-HT7 receptor in human glioblastoma cell lines. Br. J. Pharmacol..

[61] Bikfalvi A., da Costa C.A., Avril T., Barnier J.-V., Bauchet L., Brisson L., Cartron P.F., Castel H., Chevet E., Chneiweiss H. (2023). Challenges in glioblastoma research: Focus on the tumor microenvironment. Trends Cancer.

[62] DeCordova S., Shastri A., Tsolaki A.G., Yasmin H., Klein L., Singh S.K., Kishore U. (2020). Molecular heterogeneity and immunosuppressive microenvironment in glioblastoma. Front. Immunol..

[63] Gieryng A., Pszczolkowska D., Walentynowicz K.A., Rajan W.D., Kaminska B. (2017). Immune microenvironment of gliomas. Lab. Investig..

[64] Piperi C., Papavassiliou K.A., Papavassiliou A.G. (2019). Pivotal role of STAT3 in shaping glioblastoma immune microenvironment. Cells.

[65] Atkinson G.P., Nozell S.E., Benveniste E.N. (2010). NF-κB and STAT3 signaling in glioma: Targets for future therapies. Expert Rev. Neurother..

[66] Verhaak R.G., Hoadley K.A., Purdom E., Wang V., Qi Y., Wilkerson M.D., Miller C.R., Ding L., Golub T., Mesirov J.P. (2010). Integrated genomic analysis identifies clinically relevant subtypes of glioblastoma characterized by abnormalities in PDGFRA, IDH1, EGFR, and NF1. Cancer Cell.

[67] Wang Q., Hu B., Hu X., Kim H., Squatrito M., Scarpace L., DeCarvalho A.C., Lyu S., Li P., Li Y. (2017). Tumor evolution of glioma-intrinsic gene expression subtypes associates with immunological changes in the microenvironment. Cancer Cell.

[68] Bouwens Van Der Vlis T., Kros J., Mustafa D., van Wijck R., Ackermans L., van Hagen P., van der Spek P. (2018). The complement system in glioblastoma multiforme. Acta Neuropathol. Commun..

[69] Ayala-Lopez N., Watts S.W. (2021). Physiology and pharmacology of neurotransmitter transporters. Compr. Physiol..

[70] Colucci-D’Amato L., Speranza L., Volpicelli F. (2020). Neurotrophic factor BDNF, physiological functions and therapeutic potential in depression, neurodegeneration and brain cancer. Int. J. Mol. Sci..

[71] Van Den Pol A.N. (2012). Neuropeptide transmission in brain circuits. Neuron.

