# Supplementary material for: A Prognostic Neuromodulation-Related Gene Signature Identifies Immunomodulation and Tumour-Associated Hallmarks in Glioblastoma
Source: Biomedicines. 2025 Oct 28;13(11):2640. doi: 10.3390/biomedicines13112640 (PMC12650030; doi:10.3390/biomedicines13112640)

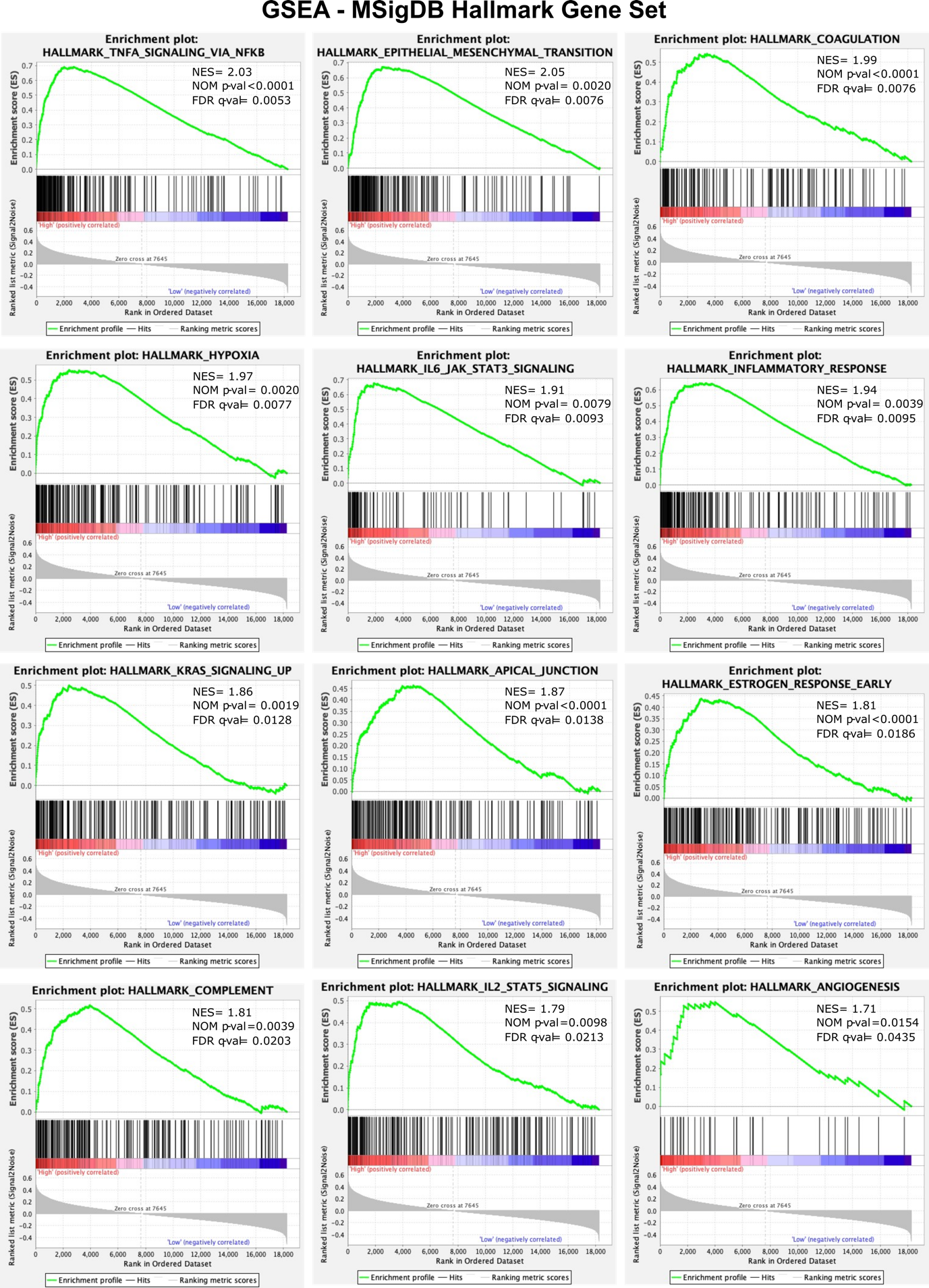


**Figure S1.** Gene set enrichment analysis (GSEA) using MSigDB Hallmark gene sets in high 10-NMRG risk score GBMs. GSEA was performed among TCGA GBM patients (n = 140) that were stratified into high- and low-risk score by the 10-NMRG signature. GSEA revealed significant enrichment of gene sets for immunomodulation hallmarks (TNF-α signalling via NF-κB, IL6-JAK-STAT3 signalling, inflammatory response, complement, IL2-STAT5 signalling) and tumour-associated hallmarks (epithelial mesenchymal transition, hypoxia, KRAS signalling activation, apical junction, early response to oestrogen, angiogenesis) in high-risk score GBM patients. Abbreviations – NES: normalised enrichment score; NOM pval: normalised *p*-value; FDR *q*-val: false discovery rate (corrected by Benjamini-Hochberg procedure) *q* value.

**Figure S2.** Gene set enrichment analysis (GSEA) using KEGG_MEDICUS pathway gene sets in high 10-NMRG risk score GBMs. GSEA was performed among TCGA GBM patients (n = 140) that were stratified into high- and low-risk score by the 10-NMRG signature. GSEA revealed significant enrichment of gene sets involved in modulating immune-related pathways (IL6, IL2 and hormone-like cytokines family mediated JAK-STAT signalling pathways) and integrins-mediated pathways (ITGA/B-RHOGAP or RHOGEF-modulated RHOA signalling, ITGA/B-FAK-modulated RAC and CDC42 signalling and ITGA/B-talin-vinculin signalling) in high-risk score GBM patients. Abbreviations – NES: normalised enrichment score; NOM pval: normalised *p*-value; FDR *q*-val: false discovery rate (corrected by Benjamini-Hochberg procedure) *q* value.
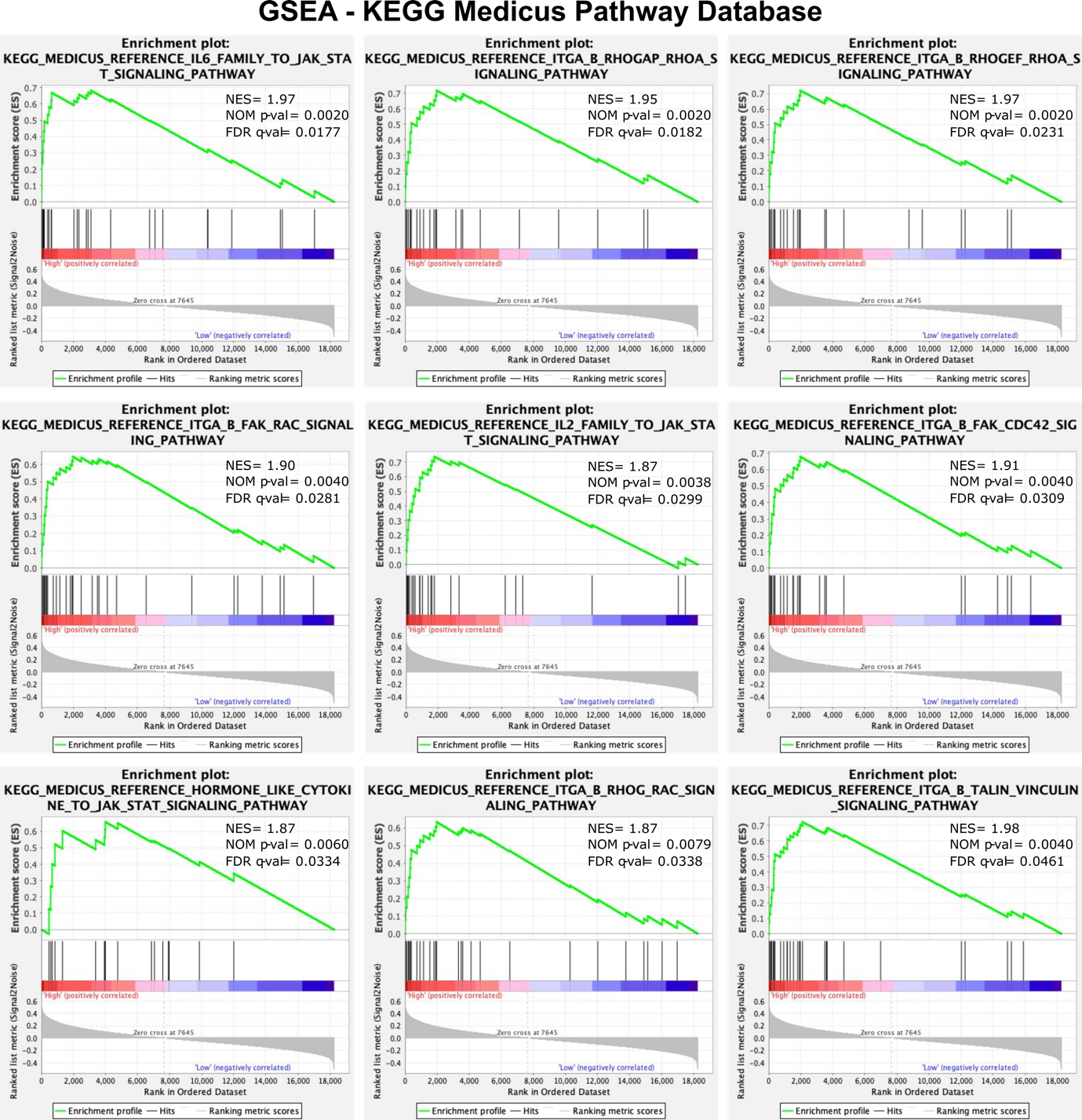


**Figure S3.** Gene set enrichment analysis (GSEA) using Reactome pathway gene sets in high 10-NMRG risk score GBMs. GSEA was performed among TCGA GBM patients (n = 140) that were stratified into high- and low-risk score by the 10-NMRG signature. GSEA revealed significant enrichment of gene sets involved in modulating immune-related pathways (TNF receptor superfamily TNFSF members mediating non canonical NF-κB pathway, complement cascade, IL4 and IL13 signalling) and extracellular matrix (ECM) remodelling (degradation of ECM, activation of matrix metalloproteinase and collagen degradation) in high-risk score GBM patients. Abbreviations – NES: normalised enrichment score; NOM pval: normalised *p*-value; FDR *q*-val: false discovery rate (corrected by Benjamini-Hochberg procedure) *q* value.
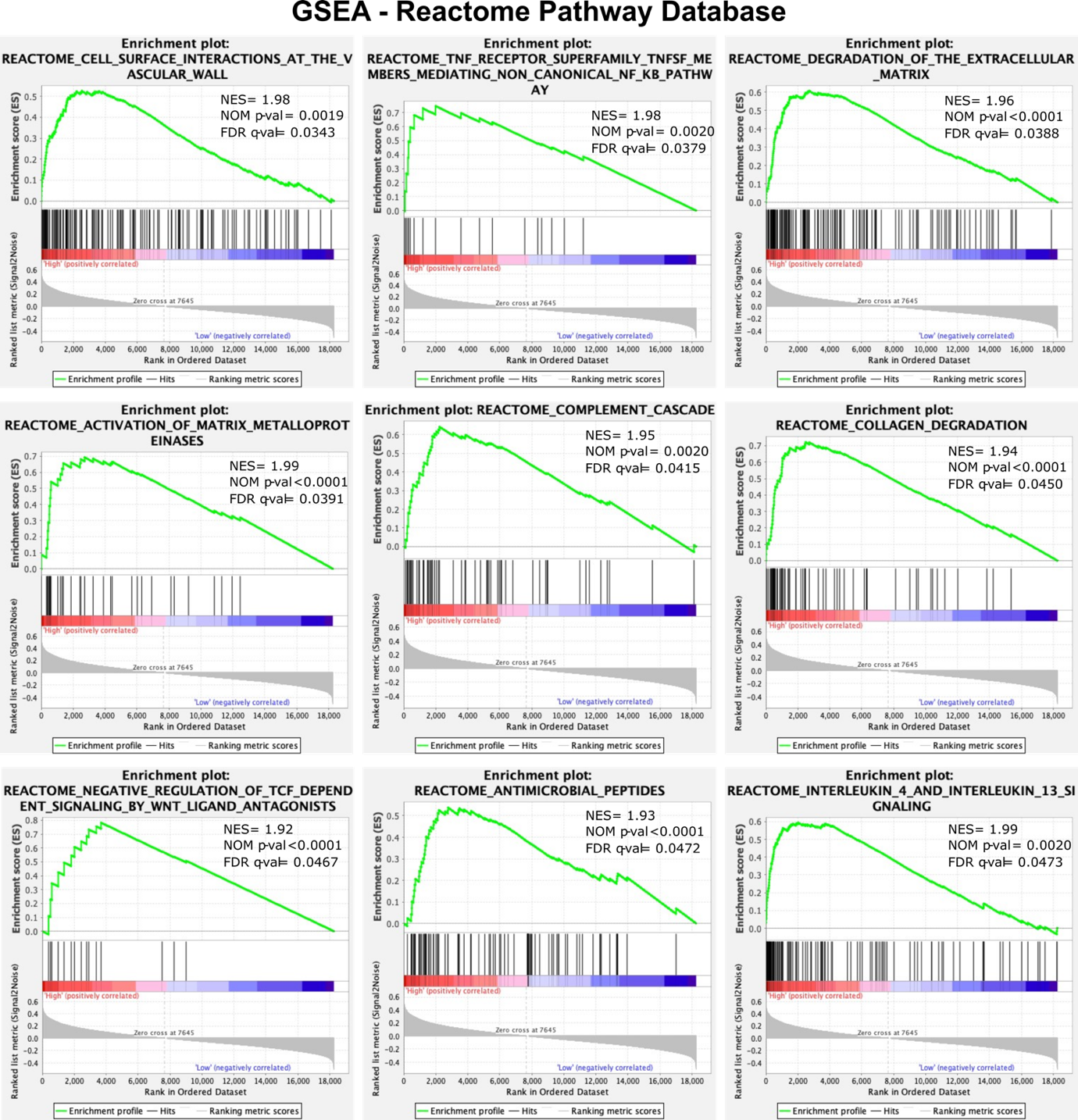

Supplement: Supplementary file 1 [file biomedicines-13-02640-s001.zip › Supplementary files/Supplementary File 2.docx]
