# Supplementary material for: A Prognostic Neuromodulation-Related Gene Signature Identifies Immunomodulation and Tumour-Associated Hallmarks in Glioblastoma
Source: Biomedicines. 2025 Oct 28;13(11):2640. doi: 10.3390/biomedicines13112640 (PMC12650030; doi:10.3390/biomedicines13112640)
Supplement: Supplementary file 1 [file biomedicines-13-02640-s001.zip › Supplementary files/Supplementary File 1.docx]

## Supplementary tables

**Table S1. Demographic and clinical characteristics of GBM patients from TCGA and CGGA databases.**

| **Database  (number of patient)** | | **TCGA (n=140)** | **CGGA (n=89)** |
| --- | --- | --- | --- |
| **Age** | |  |  |
|  | Mean (years, ± SD) | 61.51 ± 12.07 | 50.9 ± 11.4 |
| **Gender** | |  |  |
|  | Male | 90 | 57 |
|  | Female | 50 | 32 |
| **Tumour type** | |  |  |
|  | Primary | 140 | 74 |
|  | Recurrent | 0 | 15 |
|  | Unknown | 0 | 0 |
| **Subtypes** | |  |  |
|  | Classical | 38 | 0 |
|  | Mesenchymal | 47 | 0 |
|  | Neural | 26 | 0 |
|  | Proneural | 28 | 0 |
|  | Unknown | 1 | 89 |
| **Radiotherapy** | |  |  |
|  | Yes | 105 | 69 |
|  | No | 3 | 16 |
|  | Unknown | 32 | 4 |
| **Chemotherapy** | |  |  |
|  | Chemotherapy with TMZ | 87 | 63 |
|  | Chemotherapy (others) | 5 | 0 |
|  | No | 16 | 22 |
|  | Unknown | 32 | 4 |
| **Vital status** | |  |  |
|  | Living | 30 | 11 |
|  | Deceased | 109 | 78 |
|  | Unknown | 1 | 0 |

*Abbreviations: TCGA, The Cancer Genome Atlas; CGGA, Chinese Glioma Genome Atlas; SD, standard deviation; TMZ, temozolomide.*

**Table S2. List of neuropeptide and neuropeptide receptor genes included in differential expression analysis.**

| **Neuropeptide** | | | **Receptor** | |
| --- | --- | --- | --- | --- |
| **Family  name** | **Gene  symbol** | **Active ligands** | **IUPHAR name** | **Gene symbol** |
| Opioid | *PNOC* | nociceptin/orphanin FQ | NOP receptor | *OPRL1* |
|  | *PENK* | leu-enkephalin, met-enkephalin , amidorphin,  adrenorphin, peptide B, peptide E, peptide F | μ receptor | *OPRM1* |
|  | *PDYN* | dynorphin A, dynorphin B, 𝛼-neoendorphin,  𝛽-neoendorphin, dynorphin-32, leu-morphin | δ receptor | *OPRD1* |
|  | *POMC* | 𝛽-endorphin, 𝛼-endorphin, 𝛾-endorphin, | κ receptor | *OPRK1* |
|  |  | 𝛼-melanocyte-stimulating hormone (𝛼-MSH),  𝛾-melanocyte-stimulating hormone (𝛾-MSH),  𝛽-melanocyte-stimulating hormone (𝛽-MSH),  adrenocorticotropic hormone (ACTH) | MC_1_R | *MC1R* |
|  |  |  | MC_2_R | *MC2R* |
|  |  |  | MC_3_R | *MC3R* |
|  |  |  | MC_4_R | *MC4R* |
|  |  |  | MC_5_R | *MC5R* |
| Vasopressin & oxytocin | *AVP* | vasopressin | V_1A_ receptor | *AVPR1A* |
|  |  |  | V_1B_ receptor | *AVPR1B* |
|  | *OXT* | oxytocin | V_2_ receptor | *AVPR2* |
|  |  |  | OT receptor | *OXTR* |
| CCK/gastrin | *CCK* | cholecystokinin (CCK) -4, -8, -33, -39, -58 | CCK_1_ receptor | *CCKAR* |
|  | *GAST* | gastrin-14, gastrin-17, gastrin-34, gastrin-71 | CCK _2_ receptor | *CCKBR* |
| Somatostatin (SRIF) | *SST* | SRIF-14, | SST_1_ receptor | *SSTR1* |
|  |  | SRIF-28 | SST_2_ receptor | *SSTR2* |
|  | *CORT* | cortistatin-14, | SST_3_ receptor | *SSTR3* |
|  |  | cortistatin-17, | SST_4_ receptor | *SSTR4* |
|  |  | cortistatin-29 | SST_5_ receptor | *SSTR5* |
| F-amide | *RFRP* | RF-related peptide-1, RF-related peptide-2, RF-related peptide-3 | NPFF1 receptor | *NPFFR1* |
|  | *NPFF* | neuropeptide AF, neuropeptide FF,  neuropeptide SF | NPFF2 receptor | *NPFFR2* |
| Y-amide | *NPY* | neuropeptide Y (NPY) | Y_1_ receptor | *NPY1R* |
|  | *PPY* | pancreatic polypeptide (PPY) | Y_2_ receptor | *NPY2R* |
|  | *PYY* | peptide YY (PYY), PYY-(3-36) | Y_4_ receptor | *NPY4R* |
|  |  |  | Y_5_ receptor | *NPY5R* |
|  | *PRLH* | prolactin-releasing peptide-20 (PrRP-20),  prolactin-releasing peptide-31 (PrRP-31) | PrRP receptor | *PRLHR* |
| Neuropeptide B | *NPB* | neuropeptide B-23, neuropeptide B-29 | NPBW1 receptor | *NPBWR1* |
| Neuropeptide W | *NPW* | neuropeptide W-23, neuropeptide W-30 | NPBW2 receptor | *NPBWR2* |
| Neuropeptide S | *NPS* | neuropeptide S | NPS receptor | *NPSR1* |
| Neurexophilins (NXPH) | *NXPH1* | neurexophilin-1 |  |  |
|  | *NXPH2* | neurexophilin-2 |  |  |
|  | *NXPH3* | neurexophilin-3 |  |  |
|  | *NXPH4* | neurexophilin-4 | 𝛼-neurexins | *NRXN1* |
| Cerebellins (CBLN) | *CBLN1* | cerebellin-1 | 𝛽-neurexins | *NRXN2* |
|  | *CBLN2* | cerebellin-2 |  | *NRXN3* |
|  | *CBLN3* | cerebellin-3 |  |  |
|  | *CBLN4* | cerebellin-4 |  |  |
| Natriuretic  factor | *NPPA* | atrial natriuretic peptide | NPRA | *NPR1* |
|  | *NPPB* | brain natriuretic peptide (BNP) | NPRB | *NPR2* |
|  | *NPPC* | C-type natriuretic peptide (CNP) | NPRC | *NPR3* |
| Gonadotrophin-releasing hormone (GnRH) | *GNRH1* | GnRH1 | GnRH_1_ receptor | *GNRHR* |
|  | *GNRH2* | GnRH2 | GnRH _2_ receptor | *GNRHR2* |
| corticotropin- releasing hormone (CRH)-related | *CRH* | corticotropin-releasing hormone (CRH) | CRH receptor type I CRH receptor type II | *CRHR1 CRHR2* |
|  | *UCN* | urocortin I |  |  |
|  | *UCN2* | urocortin II |  |  |
|  | *UCN3* | urocortin III |  |  |
|  | *UTS2* | urotensin-2 | UT receptor | *UTS2R* |
|  | *UTS2B* | urotensin-2-related peptide,urotensin-2B |  |  |
| Calcitonin | *CALCA* | Calcitonin, katacalcin | calcitonin receptor | *CALCR* |
|  |  |  | calcitonin receptor-like  receptor | *CALCRL* |
|  | *CALCB* | calcitonin gene related peptide I (a-CGRP) | CGRP receptors composed of 3 subunits:  receptor activating-modifying protein (RAMP), calcitonin-like receptor (CLR) & receptor component protein (RCP) | *RAMP1 RAMP2 RAMP3 CLR CRCP* |
|  |  | calcitonin gene related peptide II (ß-CGRP) |  |  |
|  | *IAPP* | islet amyloid polypeptide (IAPP) / amylin |  |  |
|  | *ADM* | adrenomedullin (AM),  proadrenomedullin (PAMP) |  |  |
|  | *ADM2* | Adrenomedullin-2, intermedin-long (IMDL),  intermedin-short (IMDS) |  |  |
| Bombesin-like  peptide | *NMB* | Neuromedin B | BB_1_ receptor | *NMBR* |
|  | *GRP* | Gastrin releasing peptide (GRP): GRP(14-27) & GRP(18-27), Neuromedin C | BB_2_ receptor | *GRPR* |
| Neuromedins | *NMS* | Neuromedin S | NMU1 receptor | *NMUR1* |
|  | *NMU* | Neuromedin U | NMU2 receptor | *NMUR2* |
| Endothelin | *EDN1* | Endothelin 1 (ET-1) | ET_A_ receptor | *EDNRA* |
|  | *EDN2* | Endothelin 2 (ET-2) | ET_B_ receptor | *EDNRB* |
|  | *EDN3* | Endothelin 3 (ET-3) |  |  |
| Motilin | *MLN* | motilin, motillin-associated peptide | motilin receptor | *MLNR* |
|  | *GHRL* | ghrelin, obestatin | ghrelin receptor | *GHSR* |
| Galanin | *GAL* | galanin, galanin message associated peptide (GMAP) | GAL_1_ receptor | *GALR1* |
|  | *GALP* | galanin-like peptide (GALP) | GAL_2_ receptor | *GALR2* |
| Spexin | *SPX* | spexin | GAL_3_ receptor | *GALR3* |
| Kisspeptin | *KISS1* | kisspeptin (KP) -10, -13, -14, -54 | kisspeptin  receptor | *KISS1R* |
| Kinin / Tensin | *TAC1* | Substance P, neurokinin A, neuropeptide K, neuropeptide-𝛾 | NK_1_ receptor | *TACR1* |
|  |  |  | NK _2_ receptor | *TACR2* |
|  | *TAC3* | neuromedin K, neurokinin B | NK_3_ receptor | *TACR3* |
|  | *KNG1* | Bradykinin, kallidin,  LMW-K-kinin, HMW-K-kinin | B_1_ receptor | *BDKRB1* |
|  |  |  | B_2_ receptor | *BDKRB2* |
|  | *AGT* | angiotensin I-IV, angiotensin-(1–7) | AT_1_ receptor | *AGTR1* |
|  |  |  | AT_2_ receptor | *AGTR2* |
|  | *NTS* | neurotensin (NT), neuromedin N | NTS_1_ receptor | *NTSR1* |
|  |  |  | NTS_2_ receptor | *NTSR2* |
| Insulin | *INS* | insulin | insulin receptor | *INSR* |
|  | *IGF1* | Insulin-like growth factor 1 (IGF-1) | IGF1 receptor | *IGF1R* |
|  | *IGF2* | Insulin-like growth factor 2 (IGF-2) | IGF2 receptor | *IGF2R* |
|  | *RLN1* | relaxin-1 | RXFP1 | *RXFP1* |
|  | *RLN2* | relaxin-2 | RXFP2 | *RXFP2* |
|  | *RLN3* | relaxin-3 | RXFP3 | *RXFP3* |
|  |  |  | RXFP4 | *RXFP4* |
| Glucagon/ secretin | *GCG* | Glucagon,  glucagon-like peptide (GLP) | - | *-* |
|  | *SCT* | secretin | secretin  receptor | *SCRT* |
|  | *VIP* | vasoactive intestinal peptide 1 (VIP-1) | VIP receptor 1 | *VIPR1* |
|  |  | vasoactive intestinal peptide 2 (VIP-2) | VIP receptor 2 | *VIPR2* |
|  | *ADCYAP1* | pituitary adenylate cyclase-activating peptide (PACAP): PACAP-38, PACAP-27, PRP-48 | PACAP  receptors | *ADCYAP1R1* |
|  | *GHRH* | growth hormone-releasing hormone (GHRH) | GHRH receptor | *GHRHR* |
|  | *GIP* | gastric inhibitory peptide (GIP) | GIP receptor | *GIPR* |
| Adipose  neuropeptides | *LEP* | leptin | leptin receptor | *LEPR* |
|  | *ADIPOQ* | adiponectin | adiponectin receptor 1 | *ADIPOR1* |
|  |  |  | adiponectin receptor2 | *ADIPOR2* |
|  |  |  | T-cadherin | *CDH13* |
|  | *NAMPT* | visfatin-1 | insulin receptor | *INSR* |
|  | *RETN* | resistin | Toll-like receptor 4 (TLR4), receptor tyrosine kinase-like orphan receptor 1 (ROR1), adenylyl cyclase-associated protein 1 (CAP1) | *-* |
|  | *NUCB2* | nesfatin-1 | - | *-* |
|  | *UBL5* | beacon | - | *-* |
| Granins | *CHGA* | chromogranin A | - | *-* |
|  | *CHGB* | chromogranin B | - | *-* |
|  | *SCG2* | Secretogranin II (chromogranin C) | - | *-* |
|  | *SCG3* | Secretogranin III | - | *-* |
|  | *SCG5* | Secretogranin V | - | *-* |
|  | *VGF* | VGF (NGF-inducible protein) | complement 3a receptor | *C3AR1* |
|  |  |  | complement C1q  receptor | *C1QBP* |
| No  family | *PROK1* | prokineticin 1 | PKR_1_ | *PROKR1* |
|  | *PROK2* | prokineticin 2 | PKR_2_ | *PROKR2* |
|  | *TRH* | thyrotropin-releasing hormone (TRH) | thyrotropin-releasing  hormone receptor (TRHR) | *TRHR* |
|  | *PTHLH* | parathyroid hormone-like hormone: PTHrP-(1-36), PTHrP-(38-94), PTHrP-(107-139) | type I PTHrP  receptor | *PTH1R* |
|  | *PMCH* | melanin-concentrating hormone (MCH) | MCH_1_ receptor | *MCHR1* |
|  |  |  | MCH_2_ receptor | *MCHR2* |
|  | *HCRT* | hypocretin-1 (orexin A),  hypocretin-2 (orexin B) | OX_1_ receptor | *HCRTR1* |
|  |  |  | OX_2_ receptor | *HCRTR2* |
|  | *CARTPT* | cocaine- and amphetamine-regulated transcript (CART): CART-(1-39), CART-(42-89) | - | *-* |
|  | *AGRP* | agouti-related protein homolog (AGRP) | MC_3_R | *MC3R* |
|  |  |  | MC_4_R | *MC4R* |
|  |  |  | MC_5_R | *MC5R* |
|  | *PRL* | prolactin | prolactin receptor  (PRLR) | *PRLR* |
|  | *APLN* | apelin-13, apelin-17, apelin-36 | apelin receptor | *APLNR* |
|  | *DBI* | diazepam-binding inhibitory peptide (DBI) | GABA receptor | *-* |
|  | *SMIM20* | phoenixin-14, -20 | GPR173 | *GPR173* |

**Table S3.** **List of neurotrophic factor and neurotrophic factor receptor genes included in differential expression analysis.**

| **Family  name** | **Ligand** | | **Receptor** | |
| --- | --- | --- | --- | --- |
|  | **Gene  symbol** | **Active form** | **IUPHAR name** | **Gene symbol** |
| Neurotrophins | *NGF* | Nerve growth factor | NGF receptor,  p75NTR | *NGFR* |
|  | *BDNF* | Brain derived neurotrophic factor | Neurotrophic receptor tyrosine kinase 1 (trkA) | *NTRK1* |
|  | *NTF3* | Neurotrophin-3 | Neurotrophic receptor tyrosine kinase 2 (trkB) | *NTRK2* |
|  | *NTF4* | Neurotrophin-4 | Neurotrophic receptor tyrosine kinase 3 (trkC) | *NTRK3* |
| Glial cell-line derived neurotrophic factor (GDNF) | *GDNF* | Glial cell derived neurotrophic factor (GDNF) | GDNF family receptor 𝛼1 | *GFRA1* |
|  | *NRTN* | Neurturin | GDNF family receptor 𝛼2 | *GFRA2* |
|  | *ARTN* | Artemin | GDNF family receptor 𝛼3 | *GFRA3* |
|  | *PSPN* | Persephin | GDNF family receptor 𝛼4 | *GFRA4* |
| IL-6  receptor | *CNTF* | Ciliary neurotrophic factor | CNTF receptor | *CNTFR* |
|  | *LIF* | Leukaemia inhibitory factor | LIF receptor | *LIFR* |
|  | *CTF1* | Cardiotrophin 1 | OSM receptor | *OSMR* |
|  | *OSM* | Oncostatin M | IL6 receptor | *IL6R* |
|  | *CLCF1* | Cardiotrophin like cytokine factor 1 | IL6 receptor, 𝛽 subunit | *IL6ST* |
|  | *IL6* | Interleukin 6 |  |  |
| Fibroblast growth factor (FGF) | *FGF1* | Fibroblast growth factor 1 (acidic) | FGF receptor 1 | *FGFR1* |
|  | *FGF2* | Fibroblast growth factor 2 (basic) | FGF receptor 2 | *FGFR2* |
|  | *FGF5* | Fibroblast growth factor 5 | FGF receptor 3 | *FGFR3* |
|  | *FGF8* | Fibroblast growth factor 8 | FGF receptor 4 | *FGFR4* |
|  | *FGF9* | Fibroblast growth factor 9 |  |  |
| Transforming growth factor (TGF) | *TGFB2* | Transforming growth factor beta 2 | TGFB receptor 1 | *TGFBR1* |
|  | *TGFB3* | Transforming growth factor beta 3 | TGFB receptor 2 | *TGFBR2* |
| Hepatocyte growth factor (HGF) | *HGF* | Hepatocyte growth factor | MET receptor | *MET* |
| Platelet derived  growth factor (PDGF) | *PDGFA* | Platelet derived growth factor A | PDGF receptor 𝛼 | *PDGFRA* |
|  | *PDGFB* | Platelet derived growth factor B |  |  |
|  | *PDGFC* | Platelet derived growth factor C | PDGF receptor 𝛽 | *PDGFRB* |
|  | *PDGFD* | Platelet derived growth factor D |  |  |

**Table S4. List of neurotransmitter receptor genes included in differential expression analysis.**

| **Neurotransmitter** | **Receptor** | | |
| --- | --- | --- | --- |
|  | **Mammalian receptor** | **Receptor subunit** | **Gene symbol** |
| Glutamate (Glu) | 𝛼-amino-3-hydroxy-5-methyl-4-isoxazolepropionic acid (AMPA) receptor | glutamate ionotrophic receptor AMPA type subunit 1 | *GRIA1* |
|  |  | glutamate ionotrophic receptor AMPA type subunit 2 | *GRIA2* |
|  |  | glutamate ionotrophic receptor AMPA type subunit 3 | *GRIA3* |
|  |  | glutamate ionotrophic receptor AMPA type subunit 4 | *GRIA4* |
|  | Delta  receptor | glutamate ionotrophic receptor delta type subunit 1 | *GRID1* |
|  |  | glutamate ionotrophic receptor delta type subunit 2 | *GRID2* |
|  | Kainate  receptor | glutamate ionotrophic receptor kainate type subunit 1 | *GRIK1* |
|  |  | glutamate ionotrophic receptor kainate type subunit 2 | *GRIK2* |
|  |  | glutamate ionotrophic receptor kainate type subunit 3 | *GRIK3* |
|  |  | glutamate ionotrophic receptor kainate type subunit 4 | *GRIK4* |
|  |  | glutamate ionotrophic receptor kainate type subunit 5 | *GRIK5* |
|  | *N*-methyl-D-aspartate (NMDA) receptor | glutamate ionotrophic receptor NMDA type subunit 1 | *GRIN1* |
|  |  | glutamate ionotrophic receptor NMDA type subunit 2A | *GRIN2A* |
|  |  | glutamate ionotrophic receptor NMDA type subunit 2B | *GRIN2B* |
|  |  | glutamate ionotrophic receptor NMDA type subunit 2C | *GRIN2C* |
|  |  | glutamate ionotrophic receptor NMDA type subunit 2D | *GRIN2D* |
|  |  | glutamate ionotrophic receptor NMDA type subunit 3A | *GRIN3A* |
|  |  | glutamate ionotrophic receptor NMDA type subunit 3B | *GRIN3B* |
|  | Metabotropic  receptor | glutamate metabotropic receptor 1 | *GRM1* |
|  |  | glutamate metabotropic receptor 2 | *GRM2* |
|  |  | glutamate metabotropic receptor 3 | *GRM3* |
|  |  | glutamate metabotropic receptor 4 | *GRM4* |
|  |  | glutamate metabotropic receptor 5 | *GRM5* |
|  |  | glutamate metabotropic receptor 6 | *GRM6* |
|  |  | glutamate metabotropic receptor 7 | *GRM7* |
|  |  | glutamate metabotropic receptor 8 | *GRM8* |
| gamma- aminobutyric acid | GABA type A  (GABAA) receptor | GABA type A receptor subunit alpha 1 | *GABRA1* |
|  |  | GABA type A receptor subunit alpha 2 | *GABRA2* |
|  |  | GABA type A receptor subunit alpha 3 | *GABRA3* |
|  |  | GABA type A receptor subunit alpha 4 | *GABRA4* |
|  |  | GABA type A receptor subunit alpha 5 | *GABRA5* |
|  |  | GABA type A receptor subunit alpha 6 | *GABRA6* |
|  |  | GABA type A receptor subunit beta 1 | *GABRB1* |
|  |  | GABA type A receptor subunit beta 2 | *GABRB2* |
|  |  | GABA type A receptor subunit beta 3 | *GABRB3* |
|  |  | GABA type A receptor subunit gamma 1 | *GABRG1* |
|  |  | GABA type A receptor subunit gamma 2 | *GABRG2* |
|  |  | GABA type A receptor subunit gamma 3 | *GABRG3* |
|  |  | GABA type A receptor subunit delta | *GABRD* |
|  |  | GABA type A receptor subunit epsilon | *GABRE* |
|  |  | GABA type A receptor subunit pi | *GABRP* |
|  |  | GABA type A receptor subunit theta | *GABRQ* |
|  |  | GABA type A receptor subunit rho 1 | *GABRR1* |
|  |  | GABA type A receptor subunit rho 2 | *GABRR2* |
|  |  | GABA type A receptor subunit rho 3 | *GABRR3* |
|  | GABA type B  (GABAB) receptor | GABA type B receptor subunit 1 | *GABBR1* |
|  |  | GABA type B receptor subunit 2 | *GABBR2* |
| Acetylcholine  (ACh) | Nicotinic acetylcholine receptor  (nAChR) | Cholinergic receptor nicotinic subunit alpha 1 | *CHRNA1* |
|  |  | Cholinergic receptor nicotinic subunit alpha 2 | *CHRNA2* |
|  |  | Cholinergic receptor nicotinic subunit alpha 3 | *CHRNA3* |
|  |  | Cholinergic receptor nicotinic subunit alpha 4 | *CHRNA4* |
|  |  | Cholinergic receptor nicotinic subunit alpha 5 | *CHRNA5* |
|  |  | Cholinergic receptor nicotinic subunit alpha 6 | *CHRNA6* |
|  |  | Cholinergic receptor nicotinic subunit alpha 7 | *CHRNA7* |
|  |  | Cholinergic receptor nicotinic subunit alpha 9 | *CHRNA9* |
|  |  | Cholinergic receptor nicotinic subunit alpha 10 | *CHRNA10* |
|  |  | Cholinergic receptor nicotinic subunit beta 1 | *CHRNB1* |
|  |  | Cholinergic receptor nicotinic subunit beta 2 | *CHRNB2* |
|  |  | Cholinergic receptor nicotinic subunit beta 3 | *CHRNB3* |
|  |  | Cholinergic receptor nicotinic subunit beta 4 | *CHRNB4* |
|  | Muscarinic acetylcholine receptor (mAChR) | Cholinergic receptor muscarinic 1 | *CHRM1* |
|  |  | Cholinergic receptor muscarinic 2 | *CHRM2* |
|  |  | Cholinergic receptor muscarinic 3 | *CHRM3* |
|  |  | Cholinergic receptor muscarinic 4 | *CHRM4* |
|  |  | Cholinergic receptor muscarinic 5 | *CHRM5* |
| Noradrenaline / norepinephrine (NA/NE) | Alpha-1  adrenergic  receptor | Adrenoceptor alpha 1A | *ADRA1A* |
|  |  | Adrenoceptor alpha 1B | *ADRA1B* |
|  |  | Adrenoceptor alpha 1D | *ADRA1D* |
|  | Alpha-2  adrenergic  receptor | Adrenoceptor alpha 2A | *ADRA2A* |
|  |  | Adrenoceptor alpha 2B | *ADRA2B* |
|  |  | Adrenoceptor alpha 2C | *ADRA2C* |
|  | Beta adrenergic receptor | Adrenoceptor beta 1 | *ADRB1* |
|  |  | Adrenoceptor beta 2 | *ADRB2* |
|  |  | Adrenoceptor beta 3 | *ADRB3* |
| Dopamine  (DA) | D1-like receptor | Dopamine receptor D1 | *DRD1* |
|  |  | Dopamine receptor D5 | *DRD5* |
|  | D2-like receptor | Dopamine receptor D2 | *DRD2* |
|  |  | Dopamine receptor D3 | *DRD3* |
|  |  | Dopamine receptor D4 | *DRD4* |
|  | Trace amine-associated  receptor (TAAR) | Trace amine-associated receptor 1 | *TAAR1* |
|  |  | Trace amine-associated receptor 2 | *TAAR2* |
|  |  | Trace amine-associated receptor 5 | *TAAR5* |
|  |  | Trace amine-associated receptor 6 | *TAAR6* |
|  |  | Trace amine-associated receptor 8 | *TAAR8* |
|  |  | Trace amine-associated receptor 9 | *TAAR9* |
| Serotonin  (5-hydroxytryptamine, 5-HT) | 5-HT1 receptor | 5-hydroxytryptamine receptor 1A | *HTR1A* |
|  |  | 5-hydroxytryptamine receptor 1B | *HTR1B* |
|  |  | 5-hydroxytryptamine receptor 1D | *HTR1D* |
|  |  | 5-hydroxytryptamine receptor 1E | *HTR1E* |
|  |  | 5-hydroxytryptamine receptor 1F | *HTR1F* |
|  | 5-HT2 receptor | 5-hydroxytryptamine receptor 2A | *HTR2A* |
|  |  | 5-hydroxytryptamine receptor 2B | *HTR2B* |
|  |  | 5-hydroxytryptamine receptor 2C | *HTR2C* |
|  | 5-HT3 receptor | 5-hydroxytryptamine receptor 3A | *HTR3A* |
|  |  | 5-hydroxytryptamine receptor 3B | *HTR3B* |
|  |  | 5-hydroxytryptamine receptor 3C | *HTR3C* |
|  |  | 5-hydroxytryptamine receptor 3D | *HTR3D* |
|  |  | 5-hydroxytryptamine receptor 3E | *HTR3E* |
|  | 5-HT4 receptor | 5-hydroxytryptamine receptor 4 | *HTR4* |
|  | 5-HT5 receptor | 5-hydroxytryptamine receptor 5A | *HTR5A* |
|  | 5-HT6 receptor | 5-hydroxytryptamine receptor 6 | *HTR6* |
|  | 5-HT7 receptor | 5-hydroxytryptamine receptor 7 | *HTR7* |
| Voltage-gated  channels | Voltage-gated  calcium channels (CaV) | Calcium voltage-gated channel subunit alpha 1B | *CACNA1B* |
|  |  | Calcium voltage-gated channel auxilliary subunit alpha2 delta1 | *CACNA2D1* |
|  |  | Calcium voltage-gated channel auxilliary subunit alpha2 delta2 | *CACNA2D2* |
|  |  | Calcium voltage-gated channel auxilliary subunit beta 1 | *CACNB1* |
|  |  | Calcium voltage-gated channel auxilliary subunit beta 2 | *CACNB2* |
|  |  | Calcium voltage-gated channel auxilliary subunit beta 3 | *CACNB3* |
|  |  | Calcium voltage-gated channel auxilliary subunit beta 4 | *CACNB4* |
|  |  | Calcium voltage-gated channel auxilliary subunit gamma 2 | *CACNG2* |
|  |  | Calcium voltage-gated channel auxilliary subunit gamma 3 | *CACNG3* |
|  |  | Calcium voltage-gated channel auxilliary subunit gamma 5 | *CACNG5* |
|  |  | Calcium voltage-gated channel auxilliary subunit gamma 7 | *CACNG7* |
|  |  | Calcium voltage-gated channel auxilliary subunit gamma 8 | *CACNG8* |
|  | Voltage-gated  potassium channels (KV) | Potassium voltage-gated channel subfamily A member 2 | *KCNA2* |
|  |  | Potassium voltage-gated channel subfamily C member 1 | *KCNC1* |
|  |  | Potassium voltage-gated channel subfamily D member 2 | *KCND2* |
|  |  | Potassium inwardly rectifying channel subfamily J member 4 | *KCNJ4* |
|  |  | Potassium inwardly rectifying channel subfamily J member 9 | *KCNJ9* |
|  |  | Potassium inwardly rectifying channel subfamily J member 10 | *KCNJ10* |
|  |  | Potassium voltage-gated channel subfamily Q member 2 | *KCNQ2* |
|  |  | Potassium calcium-activated channel subfamily N member 2 | *KCNN2* |
|  |  | Potassium calcium-activated channel subfamily N member 3 | *KCNN3* |
|  |  | Potassium calcium-activated channel subfamily N member 4 | *KCNN4* |
|  | Voltage-gated  sodium channels | Sodium voltage-gated channel alpha subnunit 1 | *SCN1A* |
|  |  | Sodium voltage-gated channel alpha subnunit 2 | *SCN2A* |
|  |  | Sodium voltage-gated channel alpha subnunit 3 | *SCN3A* |
|  |  | Sodium voltage-gated channel beta subnunit 1 | *SCN1B* |
|  |  | Sodium voltage-gated channel beta subnunit 2 | *SCN2B* |
|  |  | Sodium voltage-gated channel beta subnunit 3 | *SCN3B* |
|  |  | Sodium voltage-gated channel beta subnunit 4 | *SCN4B* |

**Table S5. List of neurotransmitter system-related genes included in differential expression analysis.**

| **Neurotransmitter System** | **Proteins involved in neurotransmitters  synthesis, release, metabolism and reuptake** | |
| --- | --- | --- |
|  | **Enzymes/Transporter** | **Gene symbol** |
| Glutamate  (Glu) | Excitatory amino acid transporter 1 | *SLC1A3* |
|  | Excitatory amino acid transporter 2 | *SLC1A2* |
|  | Excitatory amino acid transporter 3 | *SLC1A1* |
|  | Excitatory amino acid transporter 4 | *SLC1A6* |
|  | Excitatory amino acid transporter 5 | *SLC1A7* |
|  | Sodium-coupled neutral amino acid transporter 3 | *SLC38A3* |
|  | Sodium-coupled neutral amino acid transporter 5 | *SLC38A5* |
|  | Sodium-coupled neutral amino acid transporter 1 | *SLC38A1* |
|  | Putative sodium-coupled neutral amino acid transporter 7 | *SLC38A7* |
|  | Putative sodium-coupled neutral amino acid transporter 8 | *SLC38A8* |
|  | Glutamic acid decarboxylase 1 | *GAD1* |
|  | Glutamic acid decarboxylase 2 | *GAD2* |
|  | Glutaminase | *GLS* |
|  | Glutamic-oxaloacetic transaminase 1 | *GOT1* |
|  | Malate dehydrogenase 1 | *MDH1* |
|  | Malate dehydrogenase 1B | *MDH1B* |
|  | Malate dehydrogenase 2 | *MDH2* |
|  | Glutamate dehydrogenase 1 | *GLUD1* |
|  | Glutamate dehydrogenase 2 | *GLUD2* |
|  | Glutamine synthetase | *LGSN* |
|  | Pyruvate carboxylase | *PC* |
|  | Pyruvate dehydrogenase E1 subunit beta | *PDHB* |
|  | Pyruvate dehydrogenase complex component X | *PDHX* |
|  | Pyruvate dehydrogenase E1 subunit alpha 1 | *PDHA1* |
| gamma- aminobutyric acid | GABA transporter 1, solute carrier family 6 member 1 | *SLC6A1* |
|  | GABA transporter 2, solute carrier family 6 member 13 | *SLC6A13* |
|  | GABA transporter 3, solute carrier family 6 member 11 | *SLC6A11* |
|  | 4-aminobutyrate aminotransferase | *ABAT* |
|  | Aldehyde dehydrogenase 5 family member A1 | *ALDH5A1* |
| Acetylcholine  (ACh) | Vesicular acetylcholine transporter | *SLC18A3* |
|  | High affinity choline transporter 1 | *SLC5A7* |
|  | Choline acetyltransferase | *CHAT* |
|  | Acetylcholinesterase | *ACHE* |
| Noradrenaline / norepinephrine (NA/NE) | Norepinephrine transporter (NET) | *SLC6A2* |
|  | Vesicular monoamine transporter 1 (VMAT1) | *SLC18A1* |
|  | Vesicular monoamine transporter 2 (VMAT2) | *SLC18A2* |
|  | Dopamine beta-hydroxylase | *DBH* |
|  | Phenylethanolamine N-methyltransferase | *PNMT* |
|  | Tyrosine hydroxylase | *TH* |
| Dopamine  (DA) | Dopamine transporter (DAT) | *SLC6A3* |
|  | Dopa decarboxylase | *DDC* |
|  | Catechol-O-methyltransferase | *COMT* |
|  | Monoamine oxidase A | *MAOA* |
|  | Monoamine oxidase B | *MAOB* |
| Serotonin  (5-hydroxytryptamine,  5-HT) | Serotonin transporter | *SLC6A4* |
|  | Tryptophan hydroxylase 1 | *TPH1* |
|  | Tryptophan hydroxylase 2 | *TPH2* |

**Table S6. List of upregulated NMRGs acting as a prognostic marker for GBM patients from TCGA cohort.**

| **Gene** | **Optimal  cut-off value** | **Coefficient** | **Hazard Ratio** | **95% CI** | **P value** |
| --- | --- | --- | --- | --- | --- |
| ***Neuropeptides*** | | | | | |
| *NMB* | 6.409 | -0.508 | 0.602 | 0.40 ~ 0.90 | 0.0145 |
| *NXPH4* | 2.483 | 0.653 | 1.922 | 1.25 ~ 2.96 | 0.0030 |
| *IGF2** | 3.197 | 0.450 | 1.568 | 1.04 ~ 2.37 | 0.0323 |
| *RETN** | 0.264 | 0.805 | 2.236 | 1.44 ~ 3.48 | 0.0004 |
| *NUCB2* | 4.93 | 0.559 | 1.748 | 1.01 ~ 3.01 | 0.0442 |
| *EDNRB** | 4.725 | -0.547 | 0.579 | 0.35 ~ 0.95 | 0.0289 |
| ***Neuropeptide receptors*** | | | | | |
| *C3AR1** | 4.041 | 0.602 | 1.827 | 1.13 ~ 2.94 | 0.0134 |
| *KISS1R* | -1.056 | 0.418 | 1.519 | 1.01 ~ 2.28 | 0.0438 |
| ***Neurotrophic factors*** | | | | | |
| *CTF1* | 1.043 | 0.956 | 2.600 | 1.2 ~ 5.65 | 0.0159 |
| *CLCF1** | 3.311 | 0.566 | 1.762 | 1.14 ~ 2.72 | 0.0108 |
| ***Neurotrophic factor receptors*** | | | | | |
| *NTRK1** | -0.413 | 0.458 | 1.580 | 1.01 ~ 2.48 | 0.0463 |
| *OSMR** | 2.339 | 1.313 | 3.716 | 1.47 ~ 9.41 | 0.0056 |
| ***Neurotransmitter receptors*** | | | | | |
| *GABRR2* | -1.181 | 0.665 | 1.944 | 1.06 ~ 3.56 | 0.0311 |
| *CHRNA1* | -0.320 | -0.423 | 0.655 | 0.43 ~ 1.00 | 0.0478 |
| *KCNN4** | -0.576 | 0.859 | 2.360 | 1.14 ~ 4.90 | 0.0213 |
| ***Neurotransmitter system-related*** | | | | | |
| *SLC38A5* | 5.486 | -0.455 | 0.635 | 0.42 ~ 0.96 | 0.0320 |
| *CHAT* | -3.626 | 0.552 | 1.736 | 1.13 ~ 2.66 | 0.0116 |
| *SLC18A3** | -1.181 | 0.632 | 1.881 | 1.18 ~ 3.00 | 0.0079 |

*Abbreviations: *, gene(s) predicting for similar survival outcome in GBM patients from TCGA and CGGA cohorts.*

**Table S7. List of downregulated NMRGs acting as a prognostic marker for GBM patients from TCGA cohort.**

| **Gene** | **Optimal  cut-off value** | **Coefficient** | **Hazard Ratio** | **95% CI** | **P value** |
| --- | --- | --- | --- | --- | --- |
| ***Neuropeptides*** | | | | | |
| *SST* | 2.690 | 0.622 | 1.862 | 1.20 ~ 2.89 | 0.006 |
| *GRP* | -0.357 | 0.655 | 1.925 | 1.21 ~ 3.06 | 0.005 |
| *VIP* | -1.732 | 0.445 | 1.561 | 1.03 ~ 2.36 | 0.034 |
| *ADCYAP1* | 1.221 | 0.606 | 1.833 | 0.99 ~ 3.38 | 0.053 |
| *TAC3* | 0.369 | 0.427 | 1.533 | 1.01 ~ 2.32 | 0.044 |
| *CHGA** | 1.868 | 0.785 | 2.193 | 1.42 ~ 3.37 | 0.000 |
| *CHGB** | 2.376 | 0.492 | 1.636 | 1.08 ~ 2.49 | 0.021 |
| *SCG5** | 5.473 | 0.933 | 2.542 | 1.17 ~ 5.53 | 0.019 |
| *CARTPT* | -4.293 | 0.446 | 1.562 | 1.03 ~ 2.37 | 0.035 |
| *CBLN3* | -0.834 | 0.751 | 2.120 | 1.06 ~ 4.24 | 0.034 |
| ***Neuropeptide receptors*** | | | | | |
| *CCKBR* | -0.576 | 0.506 | 1.658 | 1.08 ~ 2.54 | 0.020 |
| *GIPR* | -2.826 | 0.574 | 1.775 | 1.12 ~ 2.81 | 0.014 |
| *CRHR1* | 0.927 | 0.515 | 1.674 | 1.01 ~ 2.76 | 0.044 |
| *RXFP1* | -2.826 | 0.437 | 1.548 | 1.02 ~ 2.34 | 0.038 |
| *NRXN2** | 6.145 | 0.516 | 1.676 | 1.09 ~ 2.57 | 0.018 |
| *NRXN3* | 0.312 | 0.925 | 2.522 | 1.64 ~ 3.88 | 0.000 |
| *CDH13** | 1.527 | 0.731 | 2.077 | 1.23 ~ 3.50 | 0.006 |
| ***Neurotrophic factors*** | | | | | |
| *BDNF* | 0.537 | 0.564 | 1.757 | 1.11 ~ 2.78 | 0.016 |
| *FGF9** | -1.685 | 0.444 | 1.558 | 1.02 ~ 2.37 | 0.039 |
| ***Neurotrophic factor receptors*** | | | | | |
| *GFRA1* | -2.727 | 0.604 | 1.829 | 1.14 ~ 2.95 | 0.013 |
| *GFRA2* | 2.278 | 0.522 | 1.685 | 1.04 ~ 2.73 | 0.034 |
| *RET* | 1.537 | 0.543 | 1.720 | 1.06 ~ 2.80 | 0.029 |
| ***Neurotransmitter receptors*** | | | | | |
| *GRIA1* | 3.814 | 0.569 | 1.767 | 1.16 ~ 2.69 | 0.008 |
| *GRIN1* | 0.800 | 0.458 | 1.581 | 1.05 ~ 2.37 | 0.027 |
| *GRM4** | -3.816 | 0.476 | 1.610 | 1.04 ~ 2.50 | 0.034 |
| *GRM7* | -3.171 | 0.621 | 1.860 | 1.16 ~ 2.99 | 0.011 |
| *GRM8** | 1.112 | 0.428 | 1.534 | 1.01 ~ 2.32 | 0.043 |
| *GABRA1* | 0.537 | 0.475 | 1.608 | 1.06 ~ 2.43 | 0.025 |
| *GABRA2* | 0.139 | 0.649 | 1.914 | 1.23 ~ 2.97 | 0.004 |
| *GABRA3* | -1.831 | 0.639 | 1.894 | 1.07 ~ 3.35 | 0.028 |
| *GABRA4** | 0.775 | 0.653 | 1.920 | 1.06 ~ 3.47 | 0.030 |
| *GABRB2* | -2.388 | 0.516 | 1.676 | 1.06 ~ 2.65 | 0.027 |
| *GABRB3** | 1.971 | 0.501 | 1.651 | 1.07 ~ 2.54 | 0.023 |
| *GABRG2* | 0.487 | 0.512 | 1.668 | 1.10 ~ 2.53 | 0.016 |
| *GABRG3* | -3.308 | 0.669 | 1.953 | 1.28 ~ 2.99 | 0.002 |
| *GABRD** | 0.357 | 0.967 | 2.630 | 1.59 ~ 4.35 | 0.000 |
| *CHRNA2* | -3.047 | 0.510 | 1.665 | 1.09 ~ 2.55 | 0.019 |
| *CHRNB2** | 1.683 | 0.602 | 1.825 | 1.17 ~ 2.84 | 0.008 |
| *CHRM3* | -2.548 | 1.008 | 2.739 | 1.25 ~ 6.02 | 0.012 |
| *CHRM4* | -2.114 | 0.824 | 2.279 | 1.24 ~ 4.20 | 0.008 |
| *ADRA1B* | -0.320 | 0.651 | 1.918 | 1.18 ~ 3.11 | 0.008 |
| *ADRA2C* | -0.997 | 0.502 | 1.652 | 1.09 ~ 2.51 | 0.018 |
| *DRD1* | -3.308 | 0.654 | 1.923 | 1.25 ~ 2.96 | 0.003 |
| *HTR1E* | -6.506 | 0.551 | 1.735 | 1.14 ~ 2.65 | 0.011 |
| *HTR5A* | -3.458 | 0.457 | 1.579 | 1.05 ~ 2.38 | 0.028 |
| *HTR6* | -3.458 | 0.610 | 1.840 | 1.19 ~ 2.85 | 0.006 |
| *HTR7** | -1.884 | 0.553 | 1.738 | 1.12 ~ 2.70 | 0.014 |
| *CACNB2* | 0.596 | 0.554 | 1.740 | 1.07 ~ 2.84 | 0.027 |
| *CACNB3* | 2.614 | 0.854 | 2.348 | 1.13 ~ 4.88 | 0.022 |
| *CACNG3* | -1.248 | 0.488 | 1.629 | 1.09 ~ 2.45 | 0.018 |
| *CACNG8* | -2.178 | 0.752 | 2.122 | 1.06 ~ 4.24 | 0.033 |
| *CACNG2** | -3.308 | 0.419 | 1.521 | 1.01 ~ 2.28 | 0.042 |
| *KCNA2** | -0.338 | 0.672 | 1.957 | 1.04 ~ 3.70 | 0.039 |
| *KCNC1** | 0.576 | 0.544 | 1.723 | 1.11 ~ 2.67 | 0.015 |
| *KCNJ9** | 1.215 | 0.448 | 1.566 | 1.03 ~ 2.37 | 0.034 |
| *SCN2A** | 1.481 | 0.485 | 1.624 | 1.04 ~ 2.53 | 0.032 |
| *SCN3B** | 1.064 | 0.723 | 2.061 | 1.33 ~ 3.20 | 0.001 |
| *SCN1B** | 3.637 | 0.689 | 1.992 | 1.22 ~ 3.26 | 0.006 |
| *SCN2B** | -0.513 | 0.783 | 2.189 | 1.28 ~ 3.75 | 0.004 |
| ***Neurotransmitter system-related*** | | | | | |
| *SLC1A1** | 2.886 | 0.522 | 1.686 | 1.03 ~ 2.76 | 0.038 |
| *GLUD2* | -0.711 | -0.472 | 0.624 | 0.41 ~ 0.95 | 0.026 |
| *PC* | 4.930 | 0.492 | 1.635 | 1.06 ~ 2.52 | 0.026 |
| *SLC6A13** | 0.964 | 0.601 | 1.825 | 1.07 ~ 3.11 | 0.027 |
| *ACHE* | 2.832 | 0.599 | 1.819 | 1.19 ~ 2.77 | 0.005 |

*Abbreviations: *, gene(s) predicting for similar survival outcome in GBM patients from TCGA and CGGA cohorts; ^†^, gene(s) predicting for contradicting survival outcome in GBM patients from TCGA and CGGA cohorts.*

**Table S8. List of upregulated NMRGs acting as a prognostic marker for GBM patients from CGGA cohort.**

| **Gene** | **Optimal  cut-off value** | **Coefficient** | **Hazard Ratio** | **95% CI** | **P value** |
| --- | --- | --- | --- | --- | --- |
| ***Neuropeptides*** | | | | | |
| *ADM2* | 6.018 | 0.549 | 1.731 | 1.10 ~ 2.72 | 0.0172 |
| *EDN2* | 4.887 | 0.613 | 1.846 | 1.07 ~ 3.17 | 0.0266 |
| *UCN2* | 5.411 | 0.579 | 1.784 | 1.12 ~ 2.84 | 0.0144 |
| *UTS2* | 5.679 | 0.515 | 1.673 | 1.06 ~ 2.65 | 0.0281 |
| *AGT* | 10.125 | -1.105 | 0.331 | 0.16 ~ 0.71 | 0.0042 |
| *IGF2** | 9.401 | 0.615 | 1.851 | 1.17 ~ 2.93 | 0.0089 |
| *TRH* | 6.352 | 0.486 | 1.625 | 1.04 ~ 2.54 | 0.0325 |
| *DBI* | 11.526 | -0.576 | 0.562 | 0.33 ~ 0.97 | 0.0389 |
| *LEP* | 4.896 | 0.795 | 2.214 | 1.31 ~ 3.75 | 0.0031 |
| *NAMPT* | 10.620 | 0.895 | 2.448 | 1.12 ~ 5.35 | 0.0247 |
| *RETN** | 4.948 | 0.758 | 2.134 | 1.16 ~ 3.92 | 0.0146 |
| *AVPR1A* | 5.676 | 0.665 | 1.944 | 1.19 ~ 3.18 | 0.0080 |
| *OXTR* | 9.466 | 0.683 | 1.979 | 1.17 ~ 3.35 | 0.0110 |
| *CALCRL* | 8.822 | 0.555 | 1.742 | 1.05 ~ 2.88 | 0.0306 |
| *RAMP2* | 6.945 | -0.932 | 0.394 | 0.18 ~ 0.87 | 0.0206 |
| *EDNRA* | 8.917 | 0.587 | 1.798 | 1.10 ~ 2.95 | 0.0198 |
| *EDNRB** | 9.809 | -0.868 | 0.420 | 0.22 ~ 0.79 | 0.0069 |
| ***Neuropeptide receptors*** | | | | | |
| *BDKRB2* | 6.581 | 0.558 | 1.747 | 1.05 ~ 2.91 | 0.0320 |
| *C3AR1** | 9.035 | 0.768 | 2.156 | 1.33 ~ 3.50 | 0.0019 |
| *IGF2R* | 11.339 | 0.896 | 2.449 | 1.44 ~ 4.16 | 0.0009 |
| ***Neurotrophic factors*** | | | | | |
| *LIF* | 6.562 | 1.194 | 3.302 | 1.62 ~ 6.73 | 0.0010 |
| *CLCF1** | 6.123 | 0.890 | 2.435 | 1.10 ~ 5.38 | 0.0278 |
| *OSM* | 6.296 | 0.564 | 1.758 | 1.08 ~ 2.86 | 0.0228 |
| *TGFB2* | 9.704 | 0.796 | 2.216 | 1.18 ~ 4.17 | 0.0135 |
| ***Neurotrophic factor receptors*** | | | | | |
| *NTRK1** | 5.414 | 0.918 | 2.504 | 1.34 ~ 4.70 | 0.0042 |
| *GFRA3* | 6.283 | 0.859 | 2.361 | 1.20 ~ 4.64 | 0.0127 |
| *OSMR** | 9.382 | 0.865 | 2.374 | 1.13 ~ 4.97 | 0.0219 |
| ***Neurotransmitter receptors*** | | | | | |
| *GABRQ* | 5.028 | -0.937 | 0.392 | 0.21 ~ 0.74 | 0.0037 |
| *CHRNA9* | 5.318 | 1.522 | 4.580 | 1.65 ~ 12.72 | 0.0035 |
| *KCNN4** | 5.956 | 0.965 | 2.624 | 1.38 ~ 5.01 | 0.0034 |
| ***Neurotransmitter system-related*** | | | | | |
| *SLC18A3** | 4.906 | 0.628 | 1.873 | 1.14 ~ 3.07 | 0.0130 |
| *SLC18A1* | 5.083 | -0.804 | 0.448 | 0.27 ~ 0.74 | 0.0018 |

*Abbreviations: *, gene(s) predicting for similar survival outcome in GBM patients from TCGA and CGGA cohorts.*

**Table S9. List of downregulated NMRGs acting as a prognostic marker for GBM patients from CGGA cohort.**

| **Gene** | **Optimal  cut-off value** | **Coefficient** | **Hazard Ratio** | **95% CI** | **P value** |
| --- | --- | --- | --- | --- | --- |
| ***Neuropeptides*** | | | | | |
| *NPPC* | 5.247 | -0.654 | 0.520 | 0.32 ~ 0.83 | 0.007 |
| *CRH* | 5.337 | -0.731 | 0.481 | 0.29 ~ 0.80 | 0.005 |
| *CHGA** | 8.128 | -0.717 | 0.488 | 0.26 ~ 0.90 | 0.022 |
| *CHGB** | 9.364 | -0.886 | 0.412 | 0.21 ~ 0.80 | 0.009 |
| *SCG3* | 9.137 | -0.759 | 0.468 | 0.28 ~ 0.77 | 0.003 |
| *SCG5** | 10.280 | -0.874 | 0.417 | 0.24 ~ 0.74 | 0.003 |
| *NXPH3* | 9.659 | -0.975 | 0.377 | 0.18 ~ 0.77 | 0.007 |
| *CBLN1* | 6.288 | -0.580 | 0.560 | 0.31 ~ 1.00 | 0.051 |
| *CBLN2* | 6.420 | -0.802 | 0.448 | 0.23 ~ 0.87 | 0.018 |
| *CBLN4* | 7.163 | -0.650 | 0.522 | 0.28 ~ 0.97 | 0.040 |
| ***Neuropeptide receptors*** | | | | | |
| *OPRD1* | 5.053 | -0.564 | 0.569 | 0.36 ~ 0.89 | 0.014 |
| *SSTR1* | 5.475 | -0.599 | 0.550 | 0.35 ~ 0.87 | 0.012 |
| *PRLHR* | 4.882 | -0.495 | 0.610 | 0.39 ~ 0.96 | 0.032 |
| *GRPR* | 5.511 | 0.507 | 1.660 | 1.02 ~ 2.69 | 0.040 |
| *VIPR1* | 5.697 | -0.688 | 0.503 | 0.28 ~ 0.89 | 0.019 |
| *NTSR2* | 5.261 | -1.282 | 0.278 | 0.15 ~ 0.52 | 0.000 |
| *NRXN1* | 8.381 | -0.959 | 0.383 | 0.23 ~ 0.64 | 0.000 |
| *HCRTR1* | 4.943 | -0.861 | 0.423 | 0.22 ~ 0.80 | 0.008 |
| *NRXN2** | 12.258 | -0.922 | 0.398 | 0.19 ~ 0.81 | 0.011 |
| *CDH13** | 9.289 | -0.558 | 0.572 | 0.34 ~ 0.96 | 0.036 |
| ***Neurotrophic factors*** | | | | | |
| *FGF9** | 6.041 | -0.538 | 0.584 | 0.37 ~ 0.92 | 0.021 |
| ***Neurotrophic factor receptors*** | | | | | |
| *NTRK2* | 13.041 | -0.500 | 0.607 | 0.38 ~ 0.97 | 0.037 |
| *NTRK3* | 9.747 | -0.583 | 0.558 | 0.34 ~ 0.91 | 0.020 |
| *GPIHBP1* | 7.753 | -0.753 | 0.471 | 0.24 ~ 0.92 | 0.027 |
| *FGFR3* | 11.837 | -0.757 | 0.469 | 0.25 ~ 0.88 | 0.018 |
| *CNTFR* | 7.243 | -0.857 | 0.425 | 0.24 ~ 0.75 | 0.003 |
| ***Neurotransmitter receptors*** | | | | | |
| *GRIA2* | 9.144 | -0.671 | 0.511 | 0.32 ~ 0.82 | 0.006 |
| *GRIA3* | 9.981 | -0.834 | 0.435 | 0.27 ~ 0.69 | 0.000 |
| *GRIA4* | 8.790 | -0.850 | 0.427 | 0.26 ~ 0.69 | 0.001 |
| *GRID1* | 9.512 | -1.411 | 0.244 | 0.10 ~ 0.62 | 0.003 |
| *GRIN2A* | 7.748 | -0.613 | 0.542 | 0.32 ~ 0.92 | 0.022 |
| *GRIN2C* | 6.882 | -0.880 | 0.415 | 0.26 ~ 0.67 | 0.000 |
| *GRM1* | 5.582 | -0.550 | 0.577 | 0.34 ~ 0.96 | 0.036 |
| *GRM4** | 5.047 | -0.616 | 0.540 | 0.31 ~ 0.93 | 0.025 |
| *GRM5* | 7.118 | -0.704 | 0.495 | 0.25 ~ 0.99 | 0.048 |
| *GRM8** | 6.100 | -0.784 | 0.456 | 0.23 ~ 0.90 | 0.024 |
| *GABRA4** | 6.693 | -0.611 | 0.543 | 0.31 ~ 0.94 | 0.030 |
| *GABRB1* | 6.352 | -0.655 | 0.519 | 0.33 ~ 0.83 | 0.006 |
| *GABRB3** | 7.825 | -0.710 | 0.492 | 0.30 ~ 0.80 | 0.004 |
| *GABRD** | 6.084 | -0.805 | 0.447 | 0.27 ~ 0.75 | 0.002 |
| *GABBR1* | 9.888 | -0.649 | 0.522 | 0.32 ~ 0.84 | 0.008 |
| *CHRNB2** | 7.488 | -0.588 | 0.556 | 0.33 ~ 0.92 | 0.023 |
| *CHRM1* | 5.296 | -0.649 | 0.522 | 0.33 ~ 0.84 | 0.007 |
| *CHRM2* | 4.904 | -0.593 | 0.553 | 0.33 ~ 0.92 | 0.022 |
| *ADRA1A* | 6.391 | -0.708 | 0.493 | 0.31 ~ 0.78 | 0.002 |
| *HTR2A* | 6.789 | -0.628 | 0.533 | 0.30 ~ 0.96 | 0.037 |
| *HTR4* | 5.077 | -1.034 | 0.356 | 0.22 ~ 0.59 | 0.000 |
| *HTR7** | 5.747 | 0.581 | 1.787 | 1.09 ~ 2.92 | 0.021 |
| *CACNA1B* | 5.601 | -0.652 | 0.521 | 0.33 ~ 0.83 | 0.006 |
| *CACNA2D2* | 6.316 | -0.543 | 0.581 | 0.37 ~ 0.91 | 0.019 |
| *CACNG7* | 10.141 | -0.712 | 0.490 | 0.31 ~ 0.78 | 0.003 |
| *CACNG2** | 5.906 | -0.644 | 0.525 | 0.30 ~ 0.93 | 0.026 |
| *KCNA2** | 6.585 | -0.966 | 0.381 | 0.22 ~ 0.67 | 0.001 |
| *KCNC1** | 8.805 | -0.935 | 0.393 | 0.20 ~ 0.76 | 0.005 |
| *KCNJ10* | 12.300 | -0.856 | 0.425 | 0.20 ~ 0.89 | 0.023 |
| *KCNJ9** | 8.542 | -0.537 | 0.584 | 0.34 ~ 1.00 | 0.052 |
| *KCNQ2* | 7.763 | -0.739 | 0.478 | 0.29 ~ 0.78 | 0.003 |
| *KCNN2* | 6.710 | -0.624 | 0.536 | 0.33 ~ 0.87 | 0.012 |
| *KCNJ4* | 5.170 | -1.045 | 0.352 | 0.19 ~ 0.65 | 0.001 |
| *SCN2A** | 7.558 | -0.624 | 0.536 | 0.29 ~ 0.99 | 0.048 |
| *SCN3B** | 7.630 | -0.557 | 0.573 | 0.36 ~ 0.90 | 0.016 |
| *SCN1B** | 8.908 | -0.658 | 0.518 | 0.3 ~ 0.90 | 0.020 |
| *SCN2B** | 8.074 | -0.729 | 0.483 | 0.28 ~ 0.84 | 0.010 |
| ***Neurotransmitter system-related*** | | | | | |
| *SLC1A2* | 11.911 | -0.803 | 0.448 | 0.28 ~ 0.71 | 0.001 |
| *SLC1A1** | 8.440 | -0.739 | 0.478 | 0.27 ~ 0.85 | 0.012 |
| *SLC1A6* | 5.100 | -0.469 | 0.625 | 0.39 ~ 1.00 | 0.051 |
| *GAD1* | 10.143 | -0.903 | 0.405 | 0.20 ~ 0.82 | 0.012 |
| *GOT1* | 9.775 | -0.835 | 0.434 | 0.21 ~ 0.89 | 0.024 |
| *GLUD1* | 11.832 | -0.681 | 0.506 | 0.29 ~ 0.89 | 0.019 |
| *ABAT* | 11.321 | -0.773 | 0.462 | 0.28 ~ 0.75 | 0.002 |
| *ALDH5A1* | 9.033 | -0.965 | 0.381 | 0.21 ~ 0.70 | 0.002 |
| *SLC6A1* | 11.301 | -0.712 | 0.491 | 0.28 ~ 0.86 | 0.013 |
| *SLC6A13** | 5.316 | -0.948 | 0.388 | 0.19 ~ 0.77 | 0.007 |

*Abbreviations: *, gene(s) predicting for similar survival outcome in GBM patients from TCGA and CGGA cohorts; ^†^, gene(s) predicting for contradicting survival outcome in GBM patients from TCGA and CGGA cohorts.*

**Table S10. List of upregulated NMRGs identified in GBM samples from TCGA, CGGA, GSE147352 and GSE165595 database.**

| **Gene** | **TCGA** | **CGGA** | **GSE147352** | **GSE165595** |
| --- | --- | --- | --- | --- |
| ***Neuropeptides*** | | | | |
| *PPY* | ↑ | ↑ |  |  |
| *CALCB* |  | ↑ |  | ↑ |
| *ADM* | ↑ | ↑ | ↑ | ↑ |
| *ADM2* | ↑ | ↑ | ↑ |  |
| *NMB* | ↑ | ↑ | ↑ | ↑ |
| *EDN2* | ↑ | ↑ |  |  |
| *UCN2* | ↑ | ↑ | ↑ |  |
| *UTS2* | ↑ | ↓ | ↑ | ↑ |
| *AGT* | ↑ |  | ↑ |  |
| *NTS* | ↑ | ↑ |  |  |
| *GHRL* | ↑ | ↓ | ↑ |  |
| *GAL* | ↑ | ↑ | ↑ |  |
| *NPW* | ↑ | ↑ | ↑ |  |
| *NXPH4* | ↑ | ↑ | ↑ |  |
| *IGF2* | ↑ | ↑ | ↑ |  |
| *TRH* | ↑ | ↑ | ↑ |  |
| *PMCH* | ↑ | ↓ | ↑ |  |
| *APLN* | ↑ | ↑ | ↑ | ↑ |
| *KISS1* | ↑ | ↑ |  |  |
| *DBI* | ↑ | ↑ | ↑ | ↑ |
| *PROK1* | ↑ |  | ↑ |  |
| *LEP* | ↓ | ↑ | ↑ |  |
| *NAMPT* | ↑ | ↑ | ↑ | ↑ |
| *RETN* | ↑ | ↑ |  |  |
| *NUCB2* | ↑ | ↑ |  |  |
| ***Neuropeptide receptors*** | | | | |
| *MC5R* | ↑ | ↑ |  |  |
| *AVPR1A* | ↑ | ↑ | ↑ |  |
| *OXTR* | ↑ | ↑ | ↑ |  |
| *CCKAR* | ↑ | ↑ |  |  |
| *CALCR* |  | ↑ | ↑ |  |
| *CALCRL* | ↑ | ↑ | ↑ |  |
| *RAMP1* | ↑ |  | ↑ |  |
| *RAMP2* | ↑ |  | ↑ |  |
| *RAMP3* | ↑ |  |  | ↑ |
| *NPR1* | ↑ | ↑ | ↑ | ↑ |
| *EDNRA* | ↑ | ↑ | ↑ | ↑ |
| *EDNRB* | ↑ |  | ↑ |  |
| *BDKRB2* | ↑ | ↑ | ↑ |  |
| *AGTR1* | ↑ | ↑ | ↑ |  |
| *C3AR1* | ↑ |  | ↑ |  |
| *GALR1* | ↑ | ↑ | ↑ | ↑ |
| *NPSR1* | ↑ | ↑ |  |  |
| *IGF2R* | ↑ | ↑ | ↑ |  |
| *TRHR* |  | ↑ |  | ↑ |
| *APLNR* | ↑ |  | ↑ |  |
| *KISS1R* |  | ↑ | ↑ |  |
| *PROKR1* | ↑ | ↑ |  |  |
| ***Neurotrophic factors*** | | | | |
| *NTF4* | ↑ | ↑ |  |  |
| *LIF* | ↑ | ↑ | ↑ | ↑ |
| *CTF1* | ↑ |  | ↑ |  |
| *CLCF1* | ↑ | ↑ | ↑ |  |
| *OSM* | ↑ | ↑ | ↑ | ↑ |
| *TGFB2* | ↑ | ↑ | ↑ | ↑ |
| *PDGFA* | ↑ |  | ↑ |  |
| *PDGFC* | ↑ | ↑ | ↑ | ↑ |
| *PDGFD* | ↑ | ↑ | ↑ | ↑ |
| ***Neurotrophic factor receptors*** | | | | |
| *NGFR* | ↑ | ↑ | ↑ | ↑ |
| *NTRK1* | ↑ | ↑ | ↑ |  |
| *GFRA3* | ↑ | ↑ | ↑ |  |
| *FGFR1* |  | ↑ | ↑ | ↑ |
| *LIFR* | ↑ |  | ↑ |  |
| *OSMR* | ↑ | ↑ | ↑ | ↑ |
| *TGFBR1* | ↑ | ↑ | ↑ | ↑ |
| *TGFBR2* | ↑ | ↑ | ↑ |  |
| *PDGFRA* | ↑ |  | ↑ | ↑ |
| ***Neurotransmitter receptors*** | | | | |
| *GRID2* | ↑ |  |  | ↑ |
| *GRIK3* | ↑ | ↑ | ↑ | ↑ |
| *GABRQ* | ↑ | ↑ | ↑ |  |
| *GABRR1* | ↑ | ↑ | ↑ |  |
| *GABRR2* |  | ↑ | ↑ | ↑ |
| *GABRR3* | ↑ | ↑ | ↑ |  |
| *CHRNA1* | ↑ | ↑ | ↑ | ↑ |
| *CHRNA3* | ↑ | ↑ | ↑ |  |
| *CHRNA5* | ↑ | ↓ | ↑ |  |
| *CHRNA9* | ↑ | ↑ | ↑ | ↑ |
| *CHRNB4* | ↑ | ↑ |  |  |
| *KCNN4* | ↑ | ↑ | ↑ | ↑ |
| ***Neurotransmitter system-related*** | | | | |
| *SLC38A5* | ↑ |  | ↑ | ↑ |
| *LGSN* | ↑ | ↑ | ↑ |  |
| *CHAT* | ↑ | ↑ | ↑ |  |
| *SLC18A3* | ↑ | ↑ | ↑ |  |
| *SLC6A2* | ↑ | ↑ |  |  |
| *SLC18A1* | ↑ |  | ↑ |  |
| *COMT* | ↑ |  | ↑ |  |
| *MAOB* | ↑ | ↑ |  | ↑ |
| *TPH1* | ↑ | ↑ | ↑ |  |

*Abbreviations:* ↑*, upregulated genes.*

**Table S11. List of downregulated NMRGs identified in GBM samples from TCGA, CGGA, GSE147352 and GSE165595 database.**

| **Gene** | **TCGA** | **CGGA** | **GSE147352** | **GSE165595** |
| --- | --- | --- | --- | --- |
| **Neuropeptides** | | | | |
| *POMC* | ↓ |  | ↓ |  |
| *CCK* | ↓ | ↓ | ↓ | ↓ |
| *SST* | ↓ | ↓ | ↓ | ↓ |
| *CORT* | ↓ |  | ↓ | ↓ |
| *NPY* | ↓ | ↓ | ↓ |  |
| *NPPC* | ↓ | ↓ | ↓ |  |
| *GRP* | ↓ |  | ↓ |  |
| *VIP* | ↓ | ↓ | ↓ | ↓ |
| *ADCYAP1* | ↓ | ↓ | ↓ | ↓ |
| *CRH* | ↓ | ↓ | ↓ |  |
| *UCN* | ↓ |  | ↓ |  |
| *TAC1* | ↓ |  | ↓ | ↓ |
| *TAC3* | ↓ |  | ↓ |  |
| *KNG1* | ↓ |  | ↓ |  |
| *CHGA* | ↓ | ↓ | ↓ | ↓ |
| *CHGB* | ↓ | ↓ | ↓ | ↓ |
| *SCG3* | ↓ | ↓ | ↓ |  |
| *SCG5* | ↓ | ↓ | ↓ | ↓ |
| *NXPH2* | ↓ | ↓ | ↓ | ↓ |
| *NXPH3* | ↓ |  | ↓ | ↓ |
| *CARTPT* | ↓ | ↓ | ↓ | ↓ |
| *CBLN1* | ↓ |  | ↓ |  |
| *CBLN2* | ↓ | ↓ | ↓ | ↓ |
| *CBLN3* | ↓ | ↓ |  |  |
| *CBLN4* | ↓ | ↓ | ↓ | ↓ |
| **Neuropeptide receptors** | | | | |
| *OPRD1* | ↓ | ↓ | ↓ | ↓ |
| *OPRK1* | ↓ | ↓ | ↓ | ↓ |
| *OPRM1* | ↓ |  | ↓ | ↓ |
| *MC4R* | ↓ | ↓ | ↓ | ↓ |
| *OPRL1* | ↓ | ↓ | ↓ | ↓ |
| *AVPR1B* | ↓ |  | ↓ |  |
| *CCKBR* | ↓ | ↓ | ↓ | ↓ |
| *SSTR1* | ↓ | ↓ | ↓ | ↓ |
| *SSTR2* | ↓ | ↓ | ↓ | ↓ |
| *SSTR3* | ↓ | ↓ | ↓ | ↓ |
| *SSTR4* | ↓ |  | ↓ | ↓ |
| *NPFFR1* | ↓ | ↓ | ↓ | ↓ |
| *NPFFR2* | ↓ |  | ↓ | ↓ |
| *NPY1R* | ↓ | ↓ | ↓ | ↓ |
| *NPY5R* | ↓ |  | ↓ | ↓ |
| *PRLHR* | ↓ | ↓ | ↓ | ↓ |
| *GRPR* | ↑ | ↑ | ↓ | ↓ |
| *VIPR1* | ↓ | ↓ | ↓ | ↓ |
| *GIPR* | ↓ | ↓ | ↑ |  |
| *CRHR1* | ↓ | ↓ | ↓ | ↓ |
| *CRHR2* | ↓ |  | ↓ | ↓ |
| *UTS2R* | ↓ |  | ↓ | ↓ |
| *TACR3* | ↓ | ↓ |  | ↓ |
| *NTSR2* | ↓ | ↓ | ↓ |  |
| *NPBWR2* | ↓ |  | ↓ | ↓ |
| *NRXN1* | ↓ | ↓ | ↓ |  |
| *IGF1R* | ↓ |  | ↓ |  |
| *RXFP1* | ↓ | ↓ | ↓ | ↓ |
| *RXFP3* | ↓ |  | ↓ | ↓ |
| *PTH1R* | ↓ | ↓ | ↓ | ↓ |
| *MCHR2* | ↓ | ↓ | ↓ | ↓ |
| *HCRTR1* | ↓ | ↓ | ↓ | ↓ |
| *HCRTR2* | ↓ |  | ↓ | ↓ |
| *PROKR2* | ↓ |  | ↓ | ↓ |
| *NRXN2* | ↓ |  | ↓ |  |
| *NRXN3* | ↓ | ↓ | ↓ | ↓ |
| *ADIPOR2* |  | ↓ |  | ↓ |
| *CDH13* | ↓ | ↓ | ↓ |  |
| **Neurotrophic factors** | | | | |
| *NGF* | ↓ |  | ↓ | ↓ |
| *BDNF* | ↓ | ↓ | ↓ |  |
| *FGF5* |  |  | ↓ | ↓ |
| *FGF8* | ↓ |  | ↓ |  |
| *FGF9* | ↓ | ↓ | ↓ | ↓ |
| **Neurotrophic factor receptors** | | | | |
| *NTRK2* | ↓ |  | ↓ |  |
| *NTRK3* | ↓ |  | ↓ |  |
| *GFRA1* | ↓ |  | ↓ |  |
| *GFRA2* | ↓ |  | ↓ | ↓ |
| *FGFR2* | ↓ | ↓ | ↓ | ↓ |
| *FGFR3* | ↓ | ↓ | ↓ |  |
| *CNTFR* | ↓ | ↓ | ↓ |  |
| **Neurotransmitter receptors** | | | | |
| *GRIA1* | ↓ |  | ↓ | ↓ |
| *GRIA2* | ↓ | ↓ | ↓ |  |
| *GRIA3* | ↓ |  | ↓ |  |
| *GRIA4* | ↓ | ↓ | ↓ |  |
| *GRID1* | ↓ | ↓ | ↓ |  |
| *GRIK2* | ↓ | ↓ | ↓ |  |
| *GRIN1* | ↓ | ↓ | ↓ | ↓ |
| *GRIN2A* | ↓ | ↓ | ↓ | ↓ |
| *GRIN2B* | ↓ | ↓ | ↓ | ↓ |
| *GRIN2C* | ↓ | ↓ | ↓ | ↓ |
| *GRIN3A* | ↓ | ↓ | ↓ | ↓ |
| *GRM1* | ↓ | ↓ | ↓ | ↓ |
| *GRM2* | ↓ | ↓ | ↓ | ↓ |
| *GRM3* | ↓ | ↓ | ↓ | ↓ |
| *GRM4* | ↓ | ↓ | ↓ | ↓ |
| *GRM5* | ↓ | ↓ | ↓ | ↓ |
| *GRM7* | ↓ | ↓ | ↓ | ↓ |
| *GRM8* | ↓ |  | ↓ | ↓ |
| *GABRA1* | ↓ | ↓ | ↓ | ↓ |
| *GABRA2* | ↓ | ↓ | ↓ | ↓ |
| *GABRA3* | ↓ | ↓ | ↓ |  |
| *GABRA4* | ↓ | ↓ | ↓ | ↓ |
| *GABRA5* | ↓ | ↓ | ↓ | ↓ |
| *GABRA6* | ↓ |  | ↓ | ↓ |
| *GABRB1* | ↓ |  | ↓ |  |
| *GABRB2* | ↓ | ↓ | ↓ | ↓ |
| *GABRB3* | ↓ | ↓ | ↓ | ↓ |
| *GABRG1* | ↓ | ↓ | ↓ | ↓ |
| *GABRG2* | ↓ | ↓ | ↓ | ↓ |
| *GABRG3* | ↓ |  | ↓ | ↓ |
| *GABRD* | ↓ | ↓ | ↓ | ↓ |
| *GABBR1* | ↓ | ↓ | ↓ | ↓ |
| *GABBR2* | ↓ |  | ↓ | ↓ |
| *CHRNA2* | ↓ | ↓ | ↓ | ↓ |
| *CHRNA4* | ↓ | ↓ | ↓ |  |
| *CHRNA7* | ↓ | ↓ | ↓ |  |
| *CHRNB2* | ↓ | ↓ | ↓ | ↓ |
| *CHRNB3* | ↓ |  | ↓ | ↓ |
| *CHRM1* | ↓ | ↓ | ↓ | ↓ |
| *CHRM2* | ↓ | ↓ | ↓ | ↓ |
| *CHRM3* | ↓ | ↓ | ↓ |  |
| *CHRM4* | ↓ | ↓ | ↓ | ↓ |
| *CHRM5* | ↓ | ↓ |  |  |
| *ADRA1A* | ↓ |  | ↓ |  |
| *ADRA1B* | ↓ | ↓ | ↓ | ↓ |
| *ADRA2A* | ↓ | ↓ | ↓ | ↓ |
| *ADRA2C* | ↓ | ↓ | ↓ | ↓ |
| *ADRB1* | ↓ | ↓ | ↓ | ↓ |
| *ADRB3* | ↓ |  | ↓ | ↓ |
| *DRD1* | ↓ | ↓ | ↓ | ↓ |
| *DRD5* | ↓ | ↓ | ↓ | ↓ |
| *HTR1A* | ↓ | ↓ | ↓ | ↓ |
| *HTR1B* | ↓ | ↓ | ↓ | ↓ |
| *HTR1E* | ↓ | ↓ | ↓ | ↓ |
| *HTR1F* | ↓ |  | ↓ | ↓ |
| *HTR2A* | ↓ | ↓ | ↓ | ↓ |
| *HTR2C* | ↓ | ↓ | ↓ | ↓ |
| *HTR4* | ↓ |  | ↓ | ↓ |
| *HTR5A* | ↓ | ↓ | ↓ | ↓ |
| *HTR6* | ↓ |  | ↓ | ↓ |
| *HTR7* | ↓ |  | ↓ |  |
| *HTR3A* | ↓ |  | ↓ |  |
| *HTR3B* | ↓ | ↓ | ↓ | ↓ |
| *CACNA1B* | ↓ | ↓ | ↓ | ↓ |
| *CACNA2D1* | ↓ | ↓ | ↓ | ↓ |
| *CACNA2D2* | ↓ | ↓ | ↓ | ↓ |
| *CACNB1* | ↓ | ↓ | ↓ | ↓ |
| *CACNB2* | ↓ | ↓ | ↓ | ↓ |
| *CACNB3* | ↓ | ↓ | ↓ | ↓ |
| *CACNB4* | ↓ | ↓ | ↓ | ↓ |
| *CACNG3* | ↓ | ↓ | ↓ | ↓ |
| *CACNG8* | ↓ | ↓ | ↓ | ↓ |
| *CACNG7* | ↓ | ↓ | ↓ |  |
| *CACNG2* | ↓ | ↓ | ↓ | ↓ |
| *KCNA2* | ↓ | ↓ | ↓ | ↓ |
| *KCNC1* | ↓ | ↓ | ↓ | ↓ |
| *KCNJ10* | ↓ | ↓ |  |  |
| *KCNJ9* | ↓ | ↓ | ↓ | ↓ |
| *KCNQ2* | ↓ |  | ↓ | ↓ |
| *KCNN2* |  |  | ↓ | ↓ |
| *KCNJ4* | ↓ | ↓ | ↓ | ↓ |
| *SCN2A* | ↓ | ↓ | ↓ | ↓ |
| *SCN1A* | ↓ |  | ↓ |  |
| *SCN3B* | ↓ | ↓ | ↓ | ↓ |
| *SCN1B* | ↓ | ↓ | ↓ | ↓ |
| *SCN2B* | ↓ | ↓ | ↓ | ↓ |
| *SCN4B* | ↓ |  | ↓ |  |
| **Neurotransmitter system related** | | | | |
| *SLC1A2* | ↓ | ↓ | ↓ | ↓ |
| *SLC1A1* | ↓ | ↓ | ↓ |  |
| *SLC1A6* | ↓ | ↓ | ↓ | ↓ |
| *SLC1A7* | ↓ |  | ↓ |  |
| *SLC38A1* | ↓ |  | ↓ |  |
| *SLC38A8* | ↓ | ↓ | ↓ | ↓ |
| *GAD1* | ↓ | ↓ | ↓ |  |
| *GAD2* | ↓ | ↓ | ↓ | ↓ |
| *GLS* | ↓ | ↓ | ↓ | ↓ |
| *GOT1* | ↓ | ↓ | ↓ | ↓ |
| *MDH1* | ↓ | ↓ | ↓ | ↓ |
| *GLUD1* | ↓ | ↓ | ↓ | ↓ |
| *GLUD2* | ↓ | ↓ | ↓ |  |
| *PC* | ↓ | ↓ | ↓ | ↓ |
| *PDHX* | ↓ | ↓ | ↓ |  |
| *PDHA1* | ↓ |  | ↓ |  |
| *ABAT* | ↓ |  | ↓ |  |
| *ALDH5A1* | ↓ | ↓ | ↓ |  |
| *SLC6A1* | ↓ | ↓ | ↓ |  |
| *SLC6A13* | ↓ | ↓ | ↓ | ↓ |
| *ACHE* | ↓ |  | ↓ |  |
| *DBH* | ↓ |  | ↓ |  |
| *PNMT* | ↓ | ↓ | ↓ |  |
| *TPH2* | ↓ |  | ↓ | ↓ |

*Abbreviations:* ↓*, downregulated genes.*

**Table S12. Differentially expressed genes (DEGs) enriched in GO Biological Process, KEGG Pathway and Reactome Pathway terms in GBM with high 10-NMRGs risk score.**

| **Term ID** | **GO Biological Process** | **Upregulated  genes** | **Downregulated  genes** |
| --- | --- | --- | --- |
| **Immunomodulation-related** | | | |
| GO:0042110 | T cell activation | *CD7, CD28, DPP4, ITK, PIK3CD, PIK3CG, PPP3CA, PTPRC, CLEC7A, TREML2* | *IL4* |
| GO:0050853 | B cell receptor signalling pathway | *BLK, CD79A, CTLA4, NCKAP1L, MNDA, PIK3CD, PRKCB, PTPRC, SYK, TEC* |  |
| GO:0007159 | leukocyte cell-cell adhesion | *ICAM1, ITGA4, ITGB1, ITGB2, SELE, SELL, SELP, SYK, CD177, FERMT3* |  |
| GO:0002250 | adaptive immune response | *CTSL, CTSS, GPR183, IGHG1, IGHM, IGKC, ITK, PIK3CD, PIK3CG, SYK, TXK, TNFRSF11A* |  |
| GO:0043123 | positive regulation of I-kappaB  kinase/NF-kappaB signalling | *BIRC3, CD4, CD40, LTBR, LTF, PRKCB, S100A12, TLR6, NOD1, UBD, NOD2, CLEC7A, TMEM106A* |  |
| GO:0050901 | leukocyte tethering or rolling | *GCNT1, ITGA4, ITGB1, ITGB7, SELE, SELL, SELP* |  |
| GO:0030217 | T cell differentiation | *CD4, LEP, PIK3CD, PTPRC, PTPN22* | *LFNG* |
| GO:0001819 | positive regulation of cytokine  production | *ADRA2A, CD28, EREG, IL1A, IL10, ITK, PIK3CD, PIK3CG, PTGER4, TXK, BATF, PLA2R1* | *CLEC9A* |
| GO:0032755 | positive regulation of interleukin-6  production | *EREG, IL1A, IL1B, IL6, IL6R, LEP, MMP8, POU2AF1, MAPK13, SYK, TLR2, TWIST1, TLR6, TLR8, NOD2, CLEC7A, TSLP, TMEM106A, LILRA5* | *NOS2, IFIH1* |
| GO:0032760 | positive regulation of tumour  necrosis factor production | *AZU1, CD14, TNFRSF8, FCGR3A, IL1A, IL6, MMP8, ORM1, ORM2, PTPRC, SYK, THBS1, TLR2, TWIST1, NOD2, CLEC7A, TMEM106A, LILRA5, CCR2* | *OAS1, OAS2, OAS3, IFIH1* |
| GO:0019221 | cytokine-mediated signalling  pathway | *CSF1R, CTSG, EREG, FLT3, HCK, IL1A, IL1B, IL2RB, IL6, IL6R, CCL2, TNFSF11, IRAK3, CRLF2, IL31RA, CCR2* |  |
| GO:0051092 | positive regulation of NF-kappaB  transcription factor activity | *ADAM8, CAV1, CD36, CD40, IL1B, IL6, LTF, NTRK1, PRKCB, S100A8, S100A9, S100A12, TLR2, TNFSF11, TNFRSF11A, IL18RAP, IL18R1, SPHK1, TLR6, NOD1, IRAK3, RIPK4, NLRC4, NOD2, CLEC7A* |  |
| GO:0031295 | T cell costimulation | *CAV1, CD28, MAP3K8, DPP4, VAV1, TNFSF14* |  |
| GO:0030593 | neutrophil chemotaxis | *CXCL3, NCKAP1L, CXCL8, CXCR2, ITGB2, PIK3CD, PIK3CG, S100A8, S100A9, S100A12, CXCL6, XCL1, SYK* | *EDN3* |
| GO:0032743 | positive regulation of interleukin-2  production | *RUNX1, CD28, IL1A, IL1B, IRF4, PTPRC, CLEC7A, CCR2* |  |
| GO:0045087 | innate immune response | *CYBB, DMBT1, IGHM, KRT16, LCN2, NCF2, PIK3CD, PIK3CG, PTX3, RNASE3, S100A12, SYK, TLR2, VNN1, CLEC5A, NOD2, COLEC12, RNASE7, APOBEC3A, IRGM* | *IFI6, IFI27, MX1, IFIH1* |
| **EMT / cell-ECM adhesion / matrix remodelling / angiogenesis-related** | | | |
| GO:0007229 | integrin-mediated signalling pathway | *COL3A1, DAB2, FBLN1, FN1, HCK, ITGA5, ITGB1, ITGB2, ITGB3, ITGB7, SYK, TEC, SEMA7A, NRP1, ADAMTS1, ITGA11, FERMT3* | *ISG15* |
| GO:0034113 | heterotypic cell-cell adhesion | *CD1D, ITGA4, ITGA5, ITGAD, ITGB1, ITGB2, ITGB3, ITGB7, LILRB2, CD200R1* |  |
| GO:0071260 | cellular response to mechanical  stimulus | *CASP5, TNFRSF8, CD40, CNN2, IL1B, LTBR, PTGER4, TLR5, TNFSF14, TNFRSF10A, ANKRD1, TLR8* | *AQP1* |
| GO:0030335 | positive regulation of cell migration | *ADRA2A, CAV1, CCR1, COL1A1, CLDN3, CSF1R, DAB2, HBEGF, F7, GRB7, HAS2, HGF, IL1B, ITGB1, LAMB1, LAMC2, MMP7, MMP14, ROR2, PDGFRA, PIK3CD, PLAU, PPP3CA, CCL7, CCL11, CCL24, SNAI1, THBS1, TWIST1, SPHK1, CCL26, GPNMB, RHOD, CASS4, CEMIP, CLEC7A, FERMT3, MYADM, TWIST2, LRRC15, SH3RF2, FAM83H* | *EGFR, IL4, VIL1,  DAAM2, LGR6* |
| GO:0030199 | collagen fibril organization | *BMP1, COL1A1, COL1A2, COL3A1, COL5A1, COL5A2, CYP1B1, LOXL2, DDR2, TLL1, PXDN, ADAMTS2, ADAMTS14* |  |
| GO:0034446 | substrate adhesion-dependent cell  spreading | *FN1, ITGA4, ITGB3, ITGB7, LAMB1, LAMC1, NRP1, FERMT3* |  |
| GO:0007155 | cell adhesion | *CD72, CDH11, CCR1, CCR3, CCR8, COL5A1, CYP1B1, DSC2, EMP2, FPR2, CXCR3, HAS1, HCK, IBSP, ICAM1, ISLR, ITGA5, ITGA9, ITGAM, ITGB1, ITGB2, ITGB3, ITGB7, LAMB1, LAMC1, LOXL2, DDR2, CCL2, CCL11, CXCL12, SELL, SELP, SPP1, PXDN, AOC3, ITGBL1, THEMIS2, CHST4, CD96, EMILIN1, NID2, ITGA11, CD177* | *HABP2* |
| GO:0030574 | collagen catabolic process | *CTSB, CTSK, CTSL, CTSS, MMP1, MMP3, MMP7, MMP8, MMP9, MMP10, MMP12, MMP13, MMP14, FURIN* |  |
| GO:0045109 | intermediate filament organization | *BFSP1, DES, NEFL* |  |
| GO:0070372 | regulation of ERK1 and ERK2  cascade | *FN1, IL1B, ROS1, SYK, ARHGEF5* | *EGFR* |
| GO:0070374 | positive regulation of ERK1 and ERK2  cascade | *C5AR1, CALCR, CD4, CD36, CHI3L1, CCR1, CSF1R, GPR183, FPR2, ICAM1, NTRK1, P2RY6, PDGFRA, PTPRC, SHC1, SLAMF1, SEMA7A, MARCO, NRP1, PTPN22, NOD2, BMPER* | *EGFR, FGFR3, MT3* |
| GO:0022617 | extracellular matrix disassembly | *ADAM8, CTSG, CTSK, CTSS, ELANE, LAMC1, LCP1, MMP1, MMP3, MMP7, MMP8, MMP9, MMP10, MMP12, MMP13, MMP14, MMP19, FURIN, TPSAB1, ADAMTS4* |  |
| GO:0014065 | phosphatidylinositol 3-kinase signalling | *FCGR3A, IRS1, PIK3CD, PIK3CG, PIK3R5* |  |
| GO:0014068 | positive regulation of phosphatidylinositol 3-kinase signalling | *CSF3, FLT3, FN1, HCLS1, HGF, LEP, NKX3-1, OSM,  PDGFRA, SELP, PIK3AP1* |  |
| GO:0045766 | positive regulation of angiogenesis | *RUNX1, CD40, CHI3L1, CCR3, CYP1B1, ECM1, EMP2, GATA6, HK2, HMOX1, IL1A, IL1B, CXCL8, ITGB1, SERPINE1, PIK3CD, PRKCB, PTGIS, SFRP2, THBS1, ADAM12, HMGA2, FGF18, SPHK1, ANGPTL4, SMOC2, ZC3H12A, LRG1, PIK3R6* | *AQP1* |
| GO:0010575 | positive regulation of vascular endothelial growth factor production | *C3, CYP1B1, IL1A, IL1B, IL6, PTGS2, SULF1* |  |
| **Term ID** | **KEGG Pathway** | **Upregulated genes** | **Downregulated genes** |
| **Immunomodulation-related** | | | |
| hsa04630 | JAK-STAT signalling pathway | *TSLP, IL2RA, IL2RB, IL2RG, IL4R, IL7R, IL21R, CRLF2, CSF2, IL5RA, CSF2RB, IL6, IL11, OSM, LIF, CLCF1, IL6R, IL31RA, OSMR, IL10, IL24, IL10RA, IL22RA2, CSF3, LEP, CSF3R, PDGFRA, JAK3, STAT4, STAT6, SOCS3, PIM1, PIK3CD* | *IL4, IL12A, EGFR,  SOCS2* |
| hsa04613 | Neutrophil extracellular trap formation | *FCGR3A, FCGR3B, SYK, SIGLEC9, CYBB, NCF2, RAC2, TLR8, ELANE, MPO, PADI4, IGH, FCGR2A, ITGAM, ITGB2, CLEC7A, PLCB2, PRKCB, FPR1, FPR2, PIK3CD, C3, CR1, CR1L, C5AR1, TLR2, MAPK13, ITGB3, SELP, AZU1, CTSG, CAMP, AQP9* |  |
| hsa04060 | Cytokine-cytokine receptor interaction | *CCL8, CCL23, CCL13, CCL7, CCL2, CCL11, CCL24, CCL26, CCL18, CCL20, CXCL1, CXCL2, CXCL3, CXCL5, CXCL6, CXCL8, PPBP, PF4V1, CXCL13, CXCL12, CXCL14, XCL1, TSLP, CSF2, CSF3, LEP, IL6, IL11, CLCF1, LIF, OSM, IL10, IL24, IL1A, IL1B, IL1RN, IL36RN, IL36B, LTB, TNFSF14, TNFSF15, TNFSF11, CD70, TNFSF9, INHBA, BMP5, BMP8B, CCR8, CCR7, CCR4, CCR5, CCR3, CCR2, CCR1, CCR6, CXCR1, CXCR2, CXCR3, XCR1, IL2RA, IL2RB, IL2RG, IL4R, IL7R, IL21R, CRLF2, CSF2RB, IL5RA, CSF3R, IL6R, IL31RA, OSMR, IL10RA, IL1R1, IL1R2, IL1RL2, IL18R1, IL18RAP, CD4, CSF1R, TNFRSF1B, LTBR, TNFRSF6B, TNFRSF10A, TNFRSF10C, TNFRSF10D, TNFRSF11A, CD27, TNFRSF8, CD40, TNFRSF9, TNFRSF18, TGFBR2* | *IL4, IL12A, MSTN* |
| hsa04659 | Th17 cell differentiation | *IL1B, IL1R1, MAPK13, IRF4, TGFBR2, IL21R, IL2RG, JAK3, IL6, IL6R, HLA-DOA, HLA-DQA1, HLA-DQB1, CD4, PPP3CA, IL4R, STAT6, RUNX1, IL2RA, IL2RB* | *IL4* |
| hsa04610 | Complement and coagulation  cascades | *F7, F13A1, THBD, PROS1, SERPINE1, SERPINB2, PLAU, PLAUR, BDKRB1, BDKRB2, SERPINA1, SERPIND1, A2M, C1QA, C1QB, C1QC, C1R, C1S, C3, CFB, CFD, C7, VSIG4, CR1, CR1L, CR2, ITGAM, ITGB2, C5AR1, C4BPA, C4BPB, CFI, CFH, CD55* | *MASP1* |
| hsa04064 | NF-kappa B signalling pathway | *IGH, SYK, PRKCB, IL1B, IL1R1, BIRC3, CD14, CD40, TNFSF11, TNFRSF11A, LTB, TNFSF14, LTBR, BCL2A1, NFKB2, CXCL8, TNFAIP3, PTGS2, CXCL1, CXCL2, CXCL3, PLAU, CCL13, CXCL12, ICAM1* | *GADD45G* |
| hsa04062 | Chemokine signalling pathway | *CXCL1, CXCL2, CXCL3, CXCL5, CXCL6, PPBP, CXCL8, CXCL12, CXCL13, PF4V1, CXCL14, XCL1, CCL2, CCL7, CCL8, CCL11, CCL13, CCL23, CCL18, CCL20, CCL24, CCL26, CXCR2, CXCR1, CXCR3, XCR1, CCR8, CCR6, CCR4, CCR7, CCR2, CCR5, CCR1, CCR3, JAK3, ADCY7, HCK, SHC1, PIK3CD, PIK3CG, PIK3R5, PIK3R6, ITK, VAV1, RAC2, WAS, DOCK2, PLCB2, PRKCB* | *GNG7* |
| hsa04750 | Inflammatory mediator regulation of TRP channels | *BDKRB1, BDKRB2, P2RY2, PLCB2, ITPR3, TRPA1, IL1B, IL1R1, MAPK13, NTRK1, PIK3CD, TRPM8, PTGER2, PTGER4, ADCY7, TRPV4, PRKCB* | *ASIC4* |
| hsa04670 | Leukocyte transendothelial migration | *ITGAM, ITGB2, ITGA4, ITGB1, CLDN3, CLDN7, CLDN14, CLDN2, CLDN23, PIK3CD, CYBB, NCF2, MMP9, MAPK13, ICAM1, PRKCB, MYL9, CXCL12, RASSF5, ITK, TXK, VAV1, RAC2* |  |
| hsa04668 | TNF signalling pathway | *BIRC3, MAPK13, CEBPB, MAP3K8, CCL2, CCL20, CXCL1, CXCL2, CXCL3, CXCL5, CXCL6, CSF2, IL18R1, IL1B, IL6, LIF, BCL3, SOCS3, TNFAIP3, JUNB, MMP3, MMP9, MMP14, VEGFC, NOD2, ICAM1, SELE, PTGS2, TNFRSF1B, PIK3CD* |  |
| hsa04662 | B cell receptor signalling pathway | *IGH, CD79A, SYK, VAV1, RAC2, PPP3CA, PRKCB, CR2, PIK3CD, FCGR2B, LILRB2, LILRB1, LILRB5, LILRB3, LILRA6, LILRA5, CD72, PIK3AP1* |  |
| hsa04658 | Th1 and Th2 cell differentiation | *STAT4, HLA-DOA, HLA-DQA1, HLA-DQB1, CD4, PPP3CA, MAPK13, IL2RA, IL2RB, IL2RG, JAK3, IL4R, STAT6* | *IL12A, IL4* |
| hsa04660 | T cell receptor signalling pathway | *CD4, PTPRC, ITK, TEC, VAV1, GRAP2, MAPK13, PPP3CA, CD28, ICOS, PIK3CD, MAP3K8, PDCD1, CTLA4, IL10, CSF2* | *IL4* |
| **EMT / cell-ECM adhesion / matrix remodelling-related** | | | |
| hsa04512 | ECM-receptor interaction | *COL1A1, COL1A2, COL6A1, COL6A2, COL6A3, COL6A5, LAMA3, LAMA4, LAMB1, LAMC1, LAMC2, THBS1, FN1, SPP1, IBSP, HSPG2, ITGA3, ITGA4, ITGA5, ITGA9, ITGA11, ITGB1, ITGB3, ITGB4, ITGB7, SDC1, CD36, GP9* | *RELN, FREM1* |
| hsa04510 | Focal adhesion | *COL1A1, COL1A2, COL6A1, COL6A2, COL6A3, COL6A5, LAMA3, LAMA4, LAMB1, LAMC1, LAMC2, THBS1, FN1, SPP1, IBSP, ITGA3, ITGA4, ITGA5, ITGA9, ITGA11, ITGB1, ITGB3, ITGB4, ITGB7, VEGFC, HGF, PDGFRA, MET, MYL9, PRKCB, PIK3CD, VAV1, RAC2, CAV1, SHC1, BIRC3* | *RELN, EGFR* |
| hsa04915 | Estrogen signalling pathway | *ESR2, HSPA6, KRT13, KRT14, KRT15, KRT16, KRT17, KRT18, KRT19, KRT32, KRT23, MMP9, HBEGF, ADCY7, SHC1, PIK3CD, PLCB2, ITPR3* | *EGFR* |
| hsa04014 | Ras signalling pathway | *FGF3, FGF7, FGF18, IGF2, VEGFC, HGF, NTRK1, PDGFRA, CSF1R, FLT3, MET, SHC1, RASGRP4, HTR7, GRIN2A, RASSF5, RAC2, PIK3CD, PRKCB* | *EGFR, FGFR3, GNG7* |
| hsa05205 | Proteoglycans in cancer | *HCLS1, VAV1, ANK1, PIK3CD, ITPR3, TWIST1, TWIST2, CAV1, TLR2, MET, THBS1, MMP9, LUM, SDC1, PLAU, PLAUR, COL1A1, COL1A2, ITGB1, ITGB3, FN1, HGF, ITGA5, PRKCB, HBEGF, IGF2, GPC3, WNT2, WNT4, WNT7B, WNT9A, HSPG2, CTSL, MAPK13* | *EGFR, HPSE2, WNT7A* |
| hsa04810 | Regulation of actin cytoskeleton | *CXCL12, FGF3, FGF7, FGF18, INSRR, PDGFRA, FN1, ITGA3, ITGA4, ITGA5, ITGA9, ITGA11, ITGAM, ITGAD, ITGB1, ITGB2, ITGB3, ITGB4, ITGB7, BDKRB1, BDKRB2, C7, PIK3CD, VAV1, RAC2, MYL9, MYH9, NCKAP1L, ARPC1B, SCIN* | *EGFR, FGFR3* |
| hsa04010 | MAPK signalling pathway | *CACNA1C, CACNA1H, PRKCB, PPP3CA, RASGRP4, EREG, AREG, FGF3, FGF7, FGF18, IGF2, VEGFC, HGF, NTRK1, PDGFRA, CSF1R, FLT3, MET, IL1A, IL1B, IL1R1, TGFBR2, CD14, RAC2, MAP3K8, MAPK13, PTPN7, DUSP1, DUSP2, HSPA6, NFKB2* | *EGFR, FGFR3,  GADD45G* |
| hsa04015 | Rap1 signalling pathway | *GRIN2A, FPR1, ADCY7, PLCB2, FGF3, FGF7, FGF18, VEGFC, HGF, PDGFRA, CSF1R, MET, PRKCB, THBS1, RAC2, ITGB3, VAV1, RASSF5, ITGAM, ITGB2, ITGB1, MAPK13, PIK3CD* | *EGFR, FGFR3* |
| hsa04520 | Adherens junction | *RAC2, WAS, MET, SNAI1, TGFBR2* | *EGFR* |
| **Term ID** | **Reactome Pathway** | **Upregulated genes** | **Downregulated genes** |
| **Immunomodulation-related** | | | |
| R-HSA-173623 | Classical antibody-mediated complement  activation | *C1QA, C1QB, C1QC, C1R, C1S, IGHG1, IGHG3, IGHG4, IGHV1-2, IGHV1-46, IGHV3-23, IGHV3-30, IGHV3-33, IGHV3-7, IGKC, IGKV1-16, IGKV1-33, IGKV1-5, IGKV2-28, IGKV2D-28, IGKV3-15, IGKV3-20, IGKV4-1, IGLC1, IGLC2, IGLC3, IGLC7, IGLV1-51, IGLV2-14, IGLV3-19, IGLV3-21, IGLV3-25, IGLV6-57* |  |
| R-HSA-166663 | Initial triggering of complement | *C1QA, C1QB, C1QC, C1R, C1S, C3, CFB, CFD, FCN1, IGHG1, IGHG3, IGHG4, IGHV1-2, IGHV1-46, IGHV3-23, IGHV3-30, IGHV3-33, IGHV3-7, IGKC, IGKV1-16, IGKV1-33, IGKV1-5, IGKV2-28, IGKV2D-28, IGKV3-15, IGKV3-20, IGKV4-1, IGLC1, IGLC2, IGLC3, IGLC7, IGLV1-51, IGLV2-14, IGLV3-19, IGLV3-21, IGLV3-25, IGLV6-57* | *MASP1* |
| R-HSA-977606 | Regulation of Complement cascade | *C1QA, C1QB, C1QC, C1R, C1S, C3, C4BPA, C4BPB, C5AR1, C5AR2, C7, CD55, CFB, CFH, CFI, CPN2, CR1, CR2, ELANE, IGHG1, IGHG3, IGHG4, IGHV1-2, IGHV1-46, IGHV3-23, IGHV3-30, IGHV3-33, IGHV3-7, IGKC, IGKV1-16, IGKV1-33, IGKV1-5, IGKV2-28, IGKV2D-28, IGKV3-15, IGKV3-20, IGKV4-1, IGLC1, IGLC2, IGLC3, IGLC7, IGLV1-51, IGLV2-14, IGLV3-19, IGLV3-21, IGLV3-25, IGLV6-57, PROS1* |  |
| R-HSA-166786 | Creation of C4 and C2 activators | *C1QA, C1QB, C1QC, C1R, C1S, FCN1, IGHG1, IGHG3, IGHG4, IGHV1-2, IGHV1-46, IGHV3-23, IGHV3-30, IGHV3-33, IGHV3-7, IGKC, IGKV1-16, IGKV1-33, IGKV1-5, IGKV2-28, IGKV2D-28, IGKV3-15, IGKV3-20, IGKV4-1, IGLC1, IGLC2, IGLC3, IGLC7, IGLV1-51, IGLV2-14, IGLV3-19, IGLV3-21, IGLV3-25, IGLV6-57* | *MASP1* |
| R-HSA-166658 | Complement cascade | *C1QA, C1QB, C1QC, C1R, C1S, C3, C4BPA, C4BPB, C5AR1, C5AR2, C7, CD55, CFB, CFD, CFH, CFI, CPN2, CR1, CR2, ELANE, FCN1, IGHG1, IGHG3, IGHG4, IGHV1-2, IGHV1-46, IGHV3-23, IGHV3-30, IGHV3-33, IGHV3-7, IGKC, IGKV1-16, IGKV1-33, IGKV1-5, IGKV2-28, IGKV2D-28, IGKV3-15, IGKV3-20, IGKV4-1, IGLC1, IGLC2, IGLC3, IGLC7, IGLV1-51, IGLV2-14, IGLV3-19, IGLV3-21, IGLV3-25, IGLV6-57, PROS1* | *MASP1* |
| R-HSA-5690714 | CD22 mediated BCR regulation | *CD79A, IGHM, IGHV1-2, IGHV1-46, IGHV3-23, IGHV3-30, IGHV3-33, IGHV3-7, IGKC, IGKV1-16, IGKV1-33, IGKV1-5, IGKV2-28, IGKV2D-28, IGKV3-15, IGKV3-20, IGKV4-1, IGLC1, IGLC2, IGLC3, IGLC7, IGLV1-51, IGLV2-14, IGLV3-19, IGLV3-21, IGLV3-25, IGLV6-57* |  |
| R-HSA-983695 | Antigen activates B Cell Receptor  (BCR) leading to generation of second messengers | *BLK, CD79A, IGHM, IGHV1-2, IGHV1-46, IGHV3-23, IGHV3-30, IGHV3-33, IGHV3-7, IGKC, IGKV1-16, IGKV1-33, IGKV1-5, IGKV2-28, IGKV2D-28, IGKV3-15, IGKV3-20, IGKV4-1, IGLC1, IGLC2, IGLC3, IGLC7, IGLV1-51, IGLV2-14, IGLV3-19, IGLV3-21, IGLV3-25, IGLV6-57, ITPR3, PIK3AP1, PIK3CD, SYK, VAV1* |  |
| R-HSA-983705 | Signalling by the B Cell Receptor  (BCR) | *BLK, CD79A, IGHM, IGHV1-2, IGHV1-46, IGHV3-23, IGHV3-30, IGHV3-33, IGHV3-7, IGKC, IGKV1-16, IGKV1-33, IGKV1-5, IGKV2-28, IGKV2D-28, IGKV3-15, IGKV3-20, IGKV4-1, IGLC1, IGLC2, IGLC3, IGLC7, IGLV1-51, IGLV2-14, IGLV3-19, IGLV3-21, IGLV3-25, IGLV6-57, ITPR3, PIK3AP1, PIK3CD, PPP3CA, PRKCB, SYK, VAV1* |  |
| R-HSA-5668541 | TNFR2 non-canonical NF-kB pathway | *BIRC3, CD27, CD40, CD70, LTB, LTBR, NFKB2, TNFRSF11A, TNFRSF18, TNFRSF1B, TNFRSF6B, TNFRSF8, TNFRSF9, TNFSF11, TNFSF14, TNFSF15, TNFSF9* |  |
| R-HSA-451927 | Interleukin-2 family signalling | *CSF2, CSF2RB, IL21R, IL2RA, IL2RB, IL2RG, IL5RA, JAK3, PIK3CD, SHC1, STAT4, SYK* |  |
| R-HSA-512988 | Interleukin-3, Interleukin-5 and GM-CSF signalling | *CSF2, CSF2RB, HCK, IL2RA, IL2RB, IL2RG, IL5RA, JAK3, PIK3CD, SHC1, SYK, TEC, VAV1* |  |
| R-HSA-6785807 | Interleukin-4 and Interleukin-13  signalling | *ALOX5, BATF, CCL11, CCL2, CD36, CEBPD, COL1A2, CXCL8, F13A1, FN1, HGF, HMOX1, ICAM1, IGHG1, IGHG4, IL10, IL1A, IL1B, IL2RG, IL4R, IL6, IL6R, IRF4, ITGAM, ITGB1, ITGB2, JAK3, JUNB, LCN2, LIF, MMP1, MMP3, MMP9, OSM, PIM1, PTGS2, SOCS3, STAT6, TIMP1, TNFRSF1B, TWIST1* | *IL12A, IL4, NOS2* |
| R-HSA-5676594 | TNF receptor superfamily (TNFSF) members mediating non-canonical NF-kB pathway | *BIRC3, CD40, LTB, LTBR, TNFRSF11A, TNFSF11, TNFSF14* |  |
| R-HSA-1266695 | Interleukin-7 signalling | *CRLF2, HGF, IL2RG, IL7R, IRS1, JAK3, TSLP* | *SOCS2* |
| **EMT / cell-ECM adhesion / matrix remodelling-related** | | | |
| R-HSA-1474244 | Extracellular matrix organization | *A2M, ADAM12, ADAM8, ADAMTS1, ADAMTS14, ADAMTS16, ADAMTS2, ADAMTS4, ASPN, BGN, BMP1, CAPN11, CAPN12, CAPN6, COL10A1, COL12A1, COL13A1, COL14A1, COL15A1, COL17A1, COL18A1, COL19A1, COL1A1, COL1A2, COL22A1, COL24A1, COL3A1, COL5A1, COL5A2, COL6A1, COL6A2, COL6A3, COL6A5, COL7A1, COL8A1, COL8A2, CTSB, CTSG, CTSK, CTSL, CTSS, DDR2, ELANE, FBLN1, FBLN2, FBLN5, FBN1, FBN2, FN1, FURIN, HSPG2, IBSP, ICAM1, ICAM4, ITGA11, ITGA3, ITGA4, ITGA5, ITGA9, ITGAD, ITGAM, ITGB1, ITGB2, ITGB3, ITGB4, ITGB7, LAMA3, LAMA4, LAMB1, LAMC1, LAMC2, LOX, LOXL1, LOXL2, LTBP2, LUM, MATN4, MFAP2, MFAP5, MMP1, MMP10, MMP12, MMP13, MMP14, MMP19, MMP3, MMP7, MMP8, MMP9, NID2, P3H1, P4HA2, P4HA3, PCOLCE, PXDN, SDC1, SERPINE1, SPP1, THBS1, TIMP1, TLL1, TPSAB1* | *ADAMTS18, BCAN,  COL25A1, COL28A1* |
| R-HSA-1474290 | Collagen formation | *ADAMTS14, ADAMTS2, BMP1, COL10A1, COL12A1, COL13A1, COL14A1, COL15A1, COL17A1, COL18A1, COL19A1, COL1A1, COL1A2, COL22A1, COL24A1, COL3A1, COL5A1, COL5A2, COL6A1, COL6A2, COL6A3, COL6A5, COL7A1, COL8A1, COL8A2, CTSB, CTSL, CTSS, ITGB4, LAMA3, LAMC2, LOX, LOXL1, LOXL2, MMP13, MMP3, MMP7, MMP9, P3H1, P4HA2, P4HA3, PCOLCE, PXDN, TLL1* | *COL25A1, COL28A1* |
| R-HSA-202733 | Cell surface interactions at the vascular wall | *CAV1, CD177, CD84, CEACAM3, DOK2, FCER1G, FN1, GRB7, IGHA1, IGHA2, IGHM, IGHV1-2, IGHV1-46, IGHV3-23, IGHV3-30, IGHV3-33, IGHV3-7, IGKC, IGKV1-16, IGKV1-33, IGKV1-5, IGKV2-28, IGKV2D-28, IGKV3-15, IGKV3-20, IGKV4-1, IGLC1, IGLC2, IGLC3, IGLC7, IGLV1-51, IGLV2-14, IGLV3-19, IGLV3-21, IGLV3-25, IGLV6-57, ITGA3, ITGA4, ITGA5, ITGAM, ITGB1, ITGB2, ITGB3, MMP1, PF4V1, PROS1, SDC1, SELE, SELL, SELP, SHC1, SIRPG, SLC16A3, SLC7A8, THBD, TNFRSF10A, TNFRSF10D, TREM1* |  |
| R-HSA-2219530 | Constitutive Signalling by Aberrant  PI3K in Cancer | *AREG, CD28, EREG, ESR2, FGF18, FGF3, FGF7, FLT3, HBEGF, HGF, ICOS, IRS1, MET, PDGFRA, PIK3AP1, PIK3CD, RAC2, VAV1* | *EGFR, FGFR3* |
| R-HSA-2022090 | Assembly of collagen fibrils and other  multimeric structures | *BMP1, COL10A1, COL15A1, COL18A1, COL1A1, COL1A2, COL24A1, COL3A1, COL5A1, COL5A2, COL6A1, COL6A2, COL6A3, COL6A5, COL7A1, COL8A1, COL8A2, CTSB, CTSL, CTSS, ITGB4, LAMA3, LAMC2, LOX, LOXL1, LOXL2, MMP13, MMP3, MMP7, MMP9, PCOLCE, PXDN, TLL1* |  |
| R-HSA-2219528 | PI3K/AKT Signalling in Cancer | *AREG, CD28, EREG, ESR2, FGF18, FGF3, FGF7, FLT3, HBEGF, HGF, ICOS, IRS1, MET, PDGFRA, PIK3AP1, PIK3CD, RAC2, VAV1* | *EGFR, FGFR3* |
| R-HSA-6811558 | PI5P, PP2A and IER3 Regulate  PI3K/AKT Signalling | *AREG, CD28, EREG, ESR2, FGF18, FGF3, FGF7, FLT3, HBEGF, HGF, ICOS, IER3, IRS1, MET, PDGFRA, PIK3AP1, PIK3CD, RAC2, VAV1* | *EGFR, FGFR3* |
| R-HSA-3000157 | Laminin interactions | *COL18A1, HSPG2, ITGA3, ITGB1, ITGB4, LAMA3, LAMA4, LAMB1, LAMC1, LAMC2, NID2* |  |
| R-HSA-199418 | Negative regulation of the PI3K/AKT  network | *AREG, CD28, EREG, ESR2, FGF18, FGF3, FGF7, FLT3, HBEGF, HGF, ICOS, IER3, IRS1, MET, PDGFRA, PIK3AP1, PIK3CD, RAC2, VAV1* | *EGFR, FGFR3* |
| R-HSA-9013149 | RAC1 GTPase cycle | *ARHGAP30, ARHGAP4, ARHGAP9, ARHGEF5, BAIAP2L1, CAV1, CYBB, DOCK2, DOCK5, FMNL1, ITGB1, NCF2, NCKAP1L, NHS, VAV1, WAS* |  |
| R-HSA-8874081 | MET activates PTK2 signalling | *HGF, ITGA3, ITGB1, LAMA3, LAMA4, LAMB1, LAMC1, LAMC2, MET* |  |
| R-HSA-216083 | Integrin cell surface interactions | *COL13A1, COL18A1, FBN1, FN1, HSPG2, IBSP, ICAM1, ICAM4, ITGA11, ITGA3, ITGA4, ITGA5, ITGA9, ITGAD, ITGAM, ITGB1, ITGB2, ITGB3, ITGB7, LUM, SPP1, THBS1* |  |
| R-HSA-1474228 | Degradation of the extracellular  matrix | *A2M, ADAM8, ADAMTS1, ADAMTS16, ADAMTS4, BMP1, CAPN11, CAPN12, CAPN6, COL12A1, COL13A1, COL14A1, COL15A1, COL17A1, COL18A1, COL19A1, CTSB, CTSG, CTSK, CTSL, CTSS, ELANE, FURIN, HSPG2, LAMA3, LAMB1, LAMC1, LAMC2, MMP1, MMP10, MMP12, MMP13, MMP14, MMP19, MMP3, MMP7, MMP8, MMP9, SPP1, TIMP1, TLL1, TPSAB1* | *ADAMTS18, BCAN,  COL25A1* |
| R-HSA-1257604 | PIP3 activates AKT signalling | *AREG, CD28, EGR1, EREG, ESR2, FGF18, FGF3, FGF7, FLT3, HBEGF, HGF, ICOS, IER3, IRS1, MET, PDGFRA, PIK3AP1, PIK3CD, RAC2, SNAI1, VAV1* | *EGFR, FGFR3* |
